# Landscape of circular RNAs in acute myeloid leukemia and their clinical significance

**DOI:** 10.1038/s41698-026-01357-6

**Published:** 2026-03-05

**Authors:** Thi-Hau Nguyen, Manh-Hung Nguyen, Ha-Nam Nguyen, Tom Erkers, Päivi Östling, Anna Bohlin, Albin Österroos, Rozbeh Jafari, Lukas M. Orre, Janne Lehtiö, Sören Lehmann, Olli Kallioniemi, Yudi Pawitan, Trung Nghia Vu

**Affiliations:** 1 University of Engineering and Technology, Vietnam National University in Hanoi (UET-VNU), Hanoi, Vietnam; 2 International School, Vietnam National University in Hanoi, Hanoi, Vietnam; 3 Science for Life Laboratory and Department of Oncology-Pathology, Karolinska Institutet, Stockholm, Sweden; 4 Department of Medicine Huddinge, Karolinska Institutet, Unit for Hematology, Karolinska University Hospital Huddinge, Stockholm, Sweden; 5 Department of Medical Sciences, Hematology, Uppsala University Hospital, Uppsala, Sweden; 6 Institute for Molecular Medicine Finland (FIMM), HiLife, University of Helsinki, Helsinki, Finland; 7 Department of Medical Epidemiology and Biostatistics, Karolinska Institutet, Stockholm, Sweden

**Keywords:** Biomarkers, Cancer, Computational biology and bioinformatics, Genetics, Oncology

## Abstract

Circular RNAs (circRNAs) have emerged as important regulators in cancer biology, but their roles in acute myeloid leukemia (AML) remain poorly characterized due to limited sample sizes and technical challenges in RNA sequencing. Here, we analyze RNA-sequencing data from 315 Swedish AML patients to create the most comprehensive circRNA profile in AML to date. We identify 5,711 high-confidence circRNAs across 315 AML samples, including 402 differentially expressed between AML and healthy controls, with host genes enriched in hematopoietic pathways. We further discover two circRNAs including hsa_circ_0024048 (p = 2.16×10⁻⁶, FDR = 0.012) and hsa_circ_0084678 (p = 1.33×10⁻⁵, FDR = 0.075) whose high expression is associated with significantly improved overall survival, a relationship not observed in their respective host genes. Furthermore, these circRNAs are associated with sensitivities of several drugs, as validated in external datasets (p < 0.05). We identify 451 circRNAs with ELN2022 risk group–specific expression patterns, highlighting circRNA heterogeneity. Subtype analysis further reveals that hsa_circ_0080850 is specifically associated with worse survival (p = 2.13×10⁻^5^ and lower remission rates (38.9% vs 74.7%) within the ELN2022 Favorable subgroup. To conclude, this study establishes the most comprehensive circRNA landscape in AML to date and demonstrates their potential as biomarkers and therapeutic targets, suggesting further investigation into circRNA-driven precision medicine in AML.

## Introduction

Acute myeloid leukemia (AML) is a hematologic malignancy marked by clonal expansion and impaired differentiation of myeloid progenitors, leading to bone marrow failure and systemic complications^1^. Despite advances in understanding its molecular mechanisms, AML remains a clinical challenge with high relapse rates and variable prognosis due to its heterogeneity. While much focus has been placed on genetic mutations and chromosomal aberrations, several recent studies^2–4^ highlight the pivotal role of noncoding RNAs (ncRNAs) in AML pathogenesis.

Among ncRNAs, microRNAs^5^ and lncRNAs^6,7^ have been widely studied in AML. In contrast, circular RNAs (circRNAs)^8^ are a unique class of RNA molecules characterized by a covalently closed loop structure^8^, which confers higher stability and distinct regulatory potential^9,10^. CircRNAs participate in diverse biological functions, including microRNA sponging, modulation of RNA-binding proteins, and regulation of parental gene expression, thereby influencing key cellular processes such as proliferation, differentiation, and apoptosis^9^. These features suggest that circRNAs may offer novel insights into AML biology and could serve as stable biomarkers or therapeutic targets^11–13^, motivating our focus on this ncRNA class in this study.

Emerging studies have revealed widespread deregulation of circRNAs in AML, often correlating with distinct clinical and molecular subtypes and influencing disease progression and therapy resistance^11^. CircRNAs also have demonstrated their relevance as potential biomarkers and therapeutic targets in AML^12,14^. Specific circRNAs, such as circBCL11B and circMYBL2, have been implicated in leukemic transformation, highlighting their functional roles in oncogenesis^11,15^. In the studies of pediatric AML and cytogenetically normal AML, aberrant circRNA expression has been associated with prognosis and drug response^12,13^. However, current studies on circRNAs in AML are mostly based on a small number of samples^11,14^ or an AML subtype^12,13^, which cannot capture the high heterogeneity of AML. A significant technical challenge lies in the RNA-sequencing (RNA-seq) data generation protocols used in large AML cohorts such as Beat-AML^16,17^ and TCGA-AML^18^, which predominantly employ poly-A selection methods for library preparation, excluding circRNAs from RNA-seq analyses.

To address these limitations, this study will utilize a large AML dataset from Sweden, called the ClinSeq-AML cohort^19^ (*n* = 315), to comprehensively characterize the circRNA landscape in AML. The analysis will explore the relationships between circRNA expression, clinical outcomes, drug sensitivity, and AML subtypes, providing valuable insights into their functional and prognostic significance.

## Results

### CircRNA landscape in AML

A total of 48,033 circRNAs were identified in the ClinSeq-AML cohort using three detection tools, including Circall^20^, CIRI2^21^, and find_circ2^22^, with each circRNA having at least one read supporting its back-splicing junction (BSJ). We filtered the circRNAs identified by Circall, retaining only those with more than four BSJ-supporting reads in at least 1% of the patients (*n* = 3), resulting in 5725 circRNAs from 2647 genes. After excluding potential false positives overlapping with a true negative set (Supplementary Table S1) from the Beat-AML cohort (see details in the section on Circular RNA detection tools in the Methods section), 5711 circRNAs remained. The majority (74.4%) of these circRNAs were listed in the circBase database. Next, we focused our analysis on this circRNA set.

**Fig. 1:**
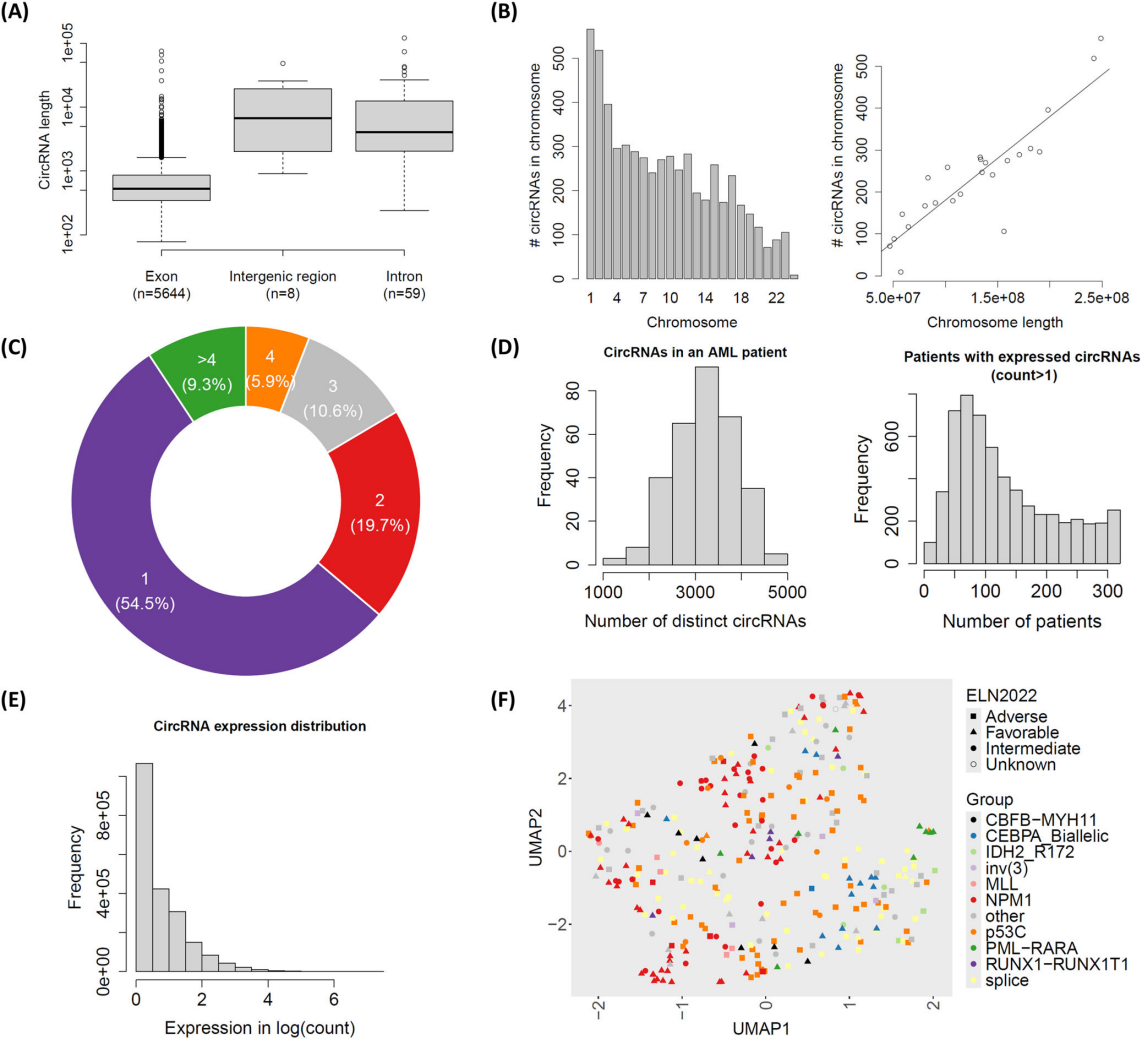
Characteristics of circular RNAs detected from the ClinSeq-AML cohort. **A** Distribution of circRNA length across three types: exonic, intergenic, and intronic circRNAs; **B** Distribution of detected circRNAs by chromosomes (left) and the linear correlation between the number of circRNAs and the length of chromosomes; **C** The proportions of host genes categorized by their number of circRNAs; **D** Distribution of the number of distinct circRNAs in AML patients (left) and the number of patients in individual expressed circRNAs (read count > 1) (right); **E** Distribution of circRNA expression (in logarithmic scale) across all AML patients; **F** UMAP plot of all AML patients using circRNA expression. Each point indicates a single AML patient. The colors and shapes are annotated by ELN2022 and molecular subtype, respectively.

As shown in Fig. 1A, the vast majority of the detected circRNAs (5644, or 98.8%) were exonic, while only 67 (1.2%) were non-exonic. The length of circRNAs ranged from 78 to 120,819 bases, with exonic circRNAs having a substantially smaller median length (525 bases) compared to intronic (4059 bases) and intergenic circRNAs (6728 bases). The number of identified circRNAs varied across chromosomes and was strongly correlated with chromosome length (Fig. 1B). Among the 2,647 host genes of circRNAs, more than 54% had a single circRNA, and 90% had fewer than five circRNAs (Fig. 1C).

The median number of distinct circRNAs per AML patient was 3246, ranging from 1309 to 4928 (Fig. 1D–left panel). These circRNAs were present in a median of 107 patients (Fig. 1D–right panel), indicating their widespread occurrence among AML cases. However, most circRNAs were expressed at low levels (Fig. 1E), and their prevalence decreased substantially when higher expression thresholds were applied. For example, when considering only circRNAs with read counts greater than 4, the median number of patients in which they were detected decreased to 18 (Supplementary Fig. S2). To further explore the circRNA expression patterns among AML patients expressed circRNA groups, we generated a UMAP plot in Fig. 1F. While the ELN2022 intermediate group did not show clear separation from the favorable or adverse groups, certain molecular subtypes, such as PML-RARA (highlighted in green at the right), revealed some group structures in the UMAP plot.

We performed Fcirc^23^ on the ClinSeq-AML cohort and identified 16 unique fusion circular RNAs (f-circRNAs) from 18 out of 315 AML patients with supporting f-circRNA read counts greater than one. Of these, one patient carried an f-circRNA derived from the PML-RARA fusion, and two patients carried f-circRNAs derived from the RUNX1-RUNX1T1 fusion. The other f-circRNAs were mainly derived from gene fusion of ANKRD28 or KMT2A. The list of f-circRNAs was provided in Supplementary Table S2.

### Differentially expressed circRNAs between AML patients and healthy individuals

**Fig. 2:**
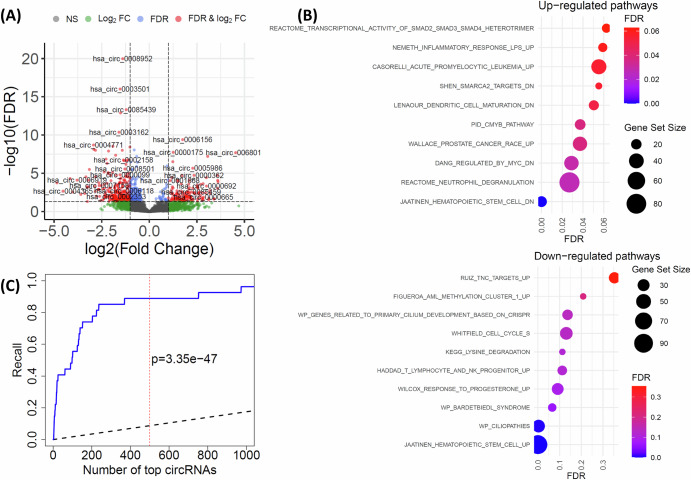
Differentially expressed (DE) circRNAs between AML and healthy samples. **A** Volcano plot of the DE circRNAs. Each point indicates one circRNA. The circRNAs are categorized into: NS (not significant), Log2FC (circRNAs only satisfy the log2 of fold-change (FC) greater than 1), FDR (circRNAs only satisfy FDR < 0.05), and FDR & Log2FC (DE circRNAs satisfy both FDR < 0.05 and the log2 of fold-change greater than 1); **B** Gene set enrichment analysis of the host genes of the DE circRNAs; **C** Recall of DE circRNAs by the Clinseq-AML cohort. The x-axis presents the number of top DE circRNAs identified by the Clinseq-AML cohort. The y-axis indicates the fraction of 27 significant DE circRNAs identified in the Lux-AML study that overlap with the top DE circRNAs from Clinseq-AML. The solid blue line is the observed recall by Clinseq-AML and the dashed black line indicates the expected recall under the null. The vertical dashed red line and the p-value indicate the results at top 500.

We identified 402 differentially expressed (DE) circRNAs between *n* = 315 AML patients in the Clinseq-AML cohort and n = 16 HSPC healthy samples, Fig. 2A. To explore their biological relevance, we extracted the host genes of these DE circRNAs for gene set enrichment analysis and reported the top associated pathways in Fig. 2B. The results revealed the host genes were enriched in hematopoietic cell-related pathways such as JAATINEN_HEMATOPOIETIC_STEM_CELL_DN, JAATINEN_HEMATOPOIETIC_STEM_CELL_UP and REACTOME_NEUTROPHIL_DEGRANULATION.

We further investigated the concordance of the DE circRNAs identified in this study and those reported in the Lux-AML study^11^, which analyzed a smaller AML dataset of 61 AML patients, Fig. 2C. To do this, we ranked the DE circRNAs from the ClinSeq-AML cohort based on their statistical significance and computed the recall as the fraction of the 27 significant DE circRNAs identified in the Lux-AML study that overlapped with the top DE circRNAs from ClinSeq-AML. As shown in Fig. 2C, the observed recall (solid blue line) was significantly higher than the expected recall under the null (dashed black line), reaching 85% at top 500 (vertical red line, *p* = 3.35×10^-47^). We noted that the cohorts represented distinct populations, and the ClinSeq-AML cohort (*n* = 315) had a substantially larger sample size than the Lux-AML cohort (n = 61). The full list of DE circRNAs was provided in Supplementary Table S3.

We further performed DE analysis between the AML samples and healthy mature hematopoietic populations (MHPs) from six healthy peripheral blood samples, data from a recent study^24^. The DE analysis identified 78 circRNAs that were significantly DE between AML and both healthy HSPCs and MHPs. These shared circRNAs accounted for 45.6% of the DE circRNAs identified between AML and MHPs, but only 19.4% of those identified between AML and HSPCs, see Supplementary Fig. S3. Given the limited sample sizes of the healthy comparison groups, these discordant results should be interpreted with caution. The full list of DE circRNAs in this analysis was provided in Supplementary Table S4.

### Association of circRNAs with survival of AML patients

**Fig. 3:**
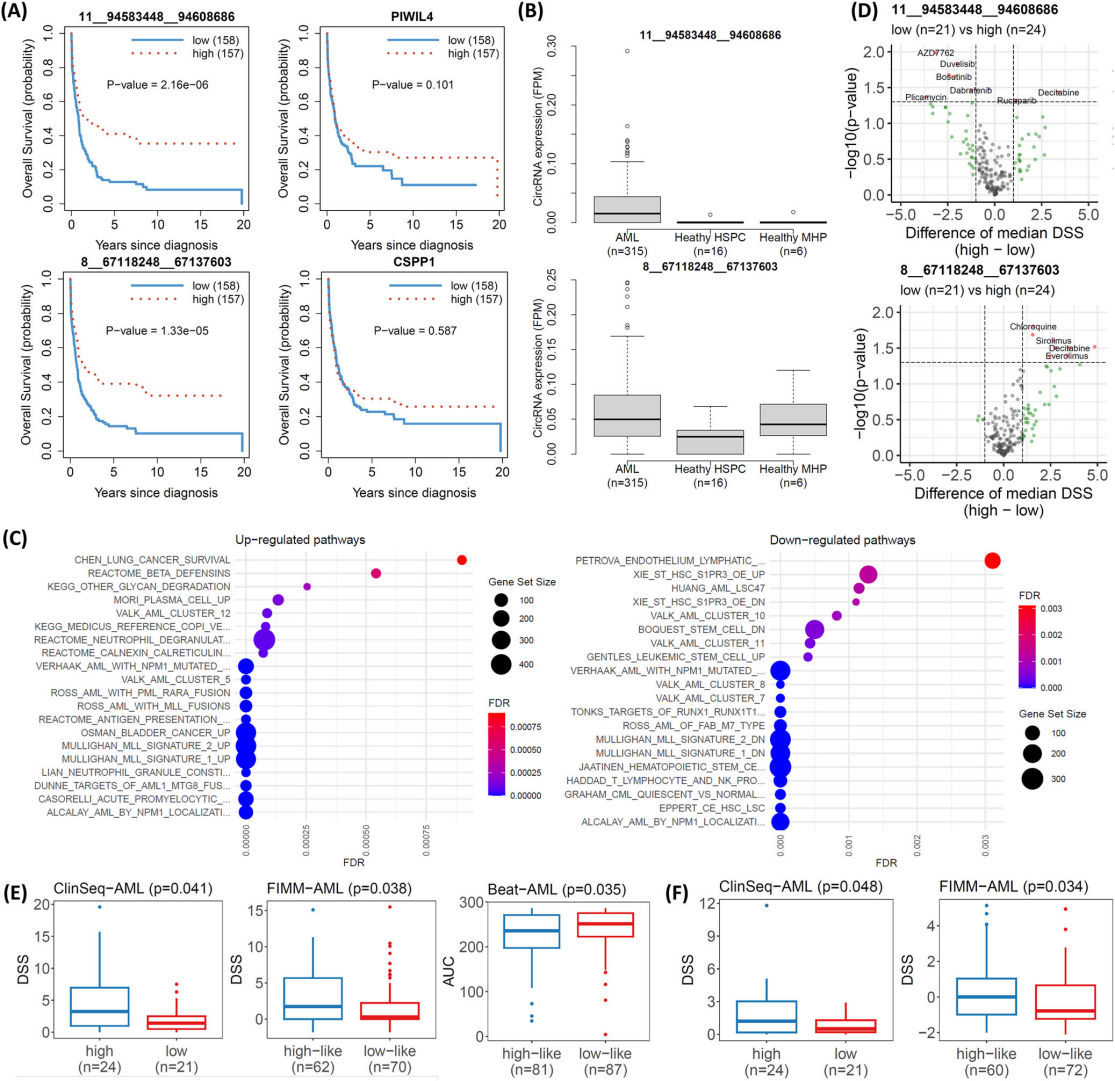
Prognosis-associated circRNAs of AML. **A** survival analysis of significant circRNAs (left) and their host genes (right); **B** differential expression of the circRNAs between AML cases and healthy people; **C** Gene set enrichment analysis of 11__94583448__94608; **D** Association of drug sensitivity (DSS) and expression of the circRNAs in the ClinSeq-AML cohort; **E** Validation of Lenalidomide for 8__67118248__67137603 using the FIMM-AML and Beat-AML cohorts; **F** Validation of Rucaparib for 11__94583448__94608686 using the FIMM-AML cohort.

Survival analysis across all AML patients identified two significant circRNAs: 11__94583448__94608 (*hsa_circ_0024048*, *p* = 2.16×10^-6^, FDR = 0.012) and 8__67118248__67137603 (*hsa_circ_0084678*, *p* = 1.33×10^-5^, FDR = 0.075), originating from the *PIWIL4* and *CSPP1* genes, respectively. Higher expression levels of these circRNAs were associated with improved survival outcomes (Fig. 3A).

In multivariable Cox regression analyses, 11__94583448__94608 was no longer statistically significant (p > 0.05) after adjustment for basic clinical and genomic characteristics, suggesting that its prognostic effect could be confounded by existing clinical variables (Supplementary Table S5). In contrast, 8__67118248__67137603 remained statistically sigsnificant after adjustment (p = 1.8 × 10⁻⁴, Supplementary Table S6), indicating that it served as an independent prognostic marker beyond the clinical and genomic factors. We further examined the associations between patient groups stratified by median circRNA expression (high vs. low) and key clinical and genomic characteristics, including risk stratification systems such as ELN2022, molecular subtypes, and FAB classification (Supplementary Figs. 4 & 5). The results showed that 11__94583448__94608 was strongly associated with key mutations (NPM1 and TP53), ELN2022 risk groups, and molecular subtypes (FDR < 0.05). In contrast, 8__67118248__67137603 showed no significant associations with these clinical or genomic variables. These findings were concordant with the results of the multivariable Cox regression analyses.

Notably, these associations were specific to the circRNAs, as the corresponding host genes did not show statistically significant survival associations (p-value > 0.05, Fig. 3A). To assess whether the observed survival associations extended to individual splice variants of the host genes, we performed an isoform-level survival analysis using annotated transcript splice variants. Two host genes PIWIL4 (of 11__94583448__94608) and CSPP1 (of 8__67118248__67137603) contained 5 and 76 expressed transcripts, respectively. Consistent with the gene-level results, none of the PIWIL4 transcripts showed a significant association with patient survival (FDR < 0.05; Supplementary Table S7). For CSPP1, one transcript (ENST00000262210) showed a statistically significant association with survival (Supplementary Table S8). However, this transcript was expressed in only 13 patients across the whole dataset (*n* = 315) and showed no correlation with the abundance of circRNA 8__67118248__67137603 (Spearman’s *ρ* = −0.02). Collectively, these results indicated that the prognostic associations were specific to circRNAs rather than to linear splice variants of their host genes.

The expression of these circRNAs in AML patients was significantly higher than in healthy HSPC individuals (Fig. 3B). However, this pattern differed when compared with healthy mature hematopoietic populations. 11__94583448__94608 is predominantly expressed in AML patients and was nearly undetectable in both healthy groups. In contrast, although circRNA 8__67118248__67137603 showed significantly higher expression in AML samples compared with healthy HSPCs, no statistically significant difference was observed when compared with healthy MHPs.

Next, gene set enrichment analysis (GSEA) using gene expression data was applied to compare low- and high-expression patient groups stratified by circRNA levels. Figure 3C shows that the top gene sets for both up-regulated and down-regulated pathways associated with 11__94583448__94608 were highly relevant to AML pathology. A similar trend was observed for the down-regulated gene sets linked to 8__67118248__67137603. However, the most significantly up-regulated pathways for this circRNA were associated with ribosome function and eukaryotic translation (Supplementary Fig. S6).

We further examined the association between patient groups (*n* = 45) and drug sensitivity using the drug sensitivity score (DSS)^25^ of 528 cancer drugs. Of note, higher DSS indicated a better response of the patient to the drug. A total of 15 drugs were significantly associated with the patient groups stratified by 11__94583448__94608 and 8__67118248__67137603, meeting the criteria of p-value < 0.05 and a median difference > 1 (see Fig. 3D, and Supplementary Table S9). For 11__94583448__94608, most identified drugs, including AZD7762, Duvelisib, Romidepsin, Bosutinib, Dabrafenib, and Plicamycin, exhibited better responses in patients with low expression levels. Conversely, two drugs, Rucaparib and Decitabine, were more effective in patients with high expression of 11__94583448__94608. In contrast, all eight significant drugs associated with 8__67118248__67137603 including Chloroquine, Bleomycin, Sirolimus, Nelarabine, Temsirolimus, Decitabine, Everolimus, and Lenalidomide showed improved responses in patients with high expression levels. Further details of the drug response analyses were provided in Supplementary Table S9.

We validated the findings using drug data from the Beat-AML and FIMM-AML cohorts. In the Beat-AML cohort, drug response was measured by the area under the curve (AUC), where a lower AUC indicates a better drug response. In contrast, drug response in the ClinSeq-AML and FIMM-AML cohorts was measured using drug sensitivity score (DSS), where a higher DSS reflected a better response. The Beat-AML cohort had AUC data for only two significant drugs: Lenalidomide and Bosutinib. The FIMM-AML cohort provided DSS data for all the significant drugs identified above. Utilizing a recent approach^12^ (further description in the section of Validation of drug sensitivity analysis in the Methods section), we stratified patients in the Beat-AML and FIMM-AML cohorts into low-like and high-like groups. We then analyzed the association between these groups and drug sensitivities. For 8__67118248__67137603, the result for Lenalidomide was validated across both the Beat-AML and FIMM-AML datasets (Fig. 3E). Patients in the high-expression group exhibited higher ***s***DSS in the ClinSeq-AML cohort (*p* = 0.041) and the FIMM-AML cohort (*p* = 0.038), as well as lower AUC in the Beat-AML cohort (*p* = 0.035), indicating better responsiveness to Lenalidomide. Additionally, the FIMM-AML cohort validated the association between 11__94583448__94608 and Rucaparib, with the high-expression group showing significantly higher DSS (*p* = 0.034, Fig. 3F).

We further performed drug-sensitivity analyses based on high versus low expression of the circRNA host genes. Because gene expression and drug-sensitivity data were available across all three cohorts, we used both the Beat-AML and FIMM-AML cohorts for validation. In the ClinSeq-AML cohort, we identified 8 and 9 drugs significantly associated with PIWIL4 and CSPP1 expression, respectively (Supplementary Table S10). Only one drug, Nelarabine, was shared between the circRNA (8__67118248__67137603) and its host gene (CSPP1). However, this association was not validated in either the Beat-AML or FIMM-AML cohorts for both the circRNA and the host gene (Supplementary Table S10).

### ELN2022 subgroup-specific circRNAs

**Fig. 4:**
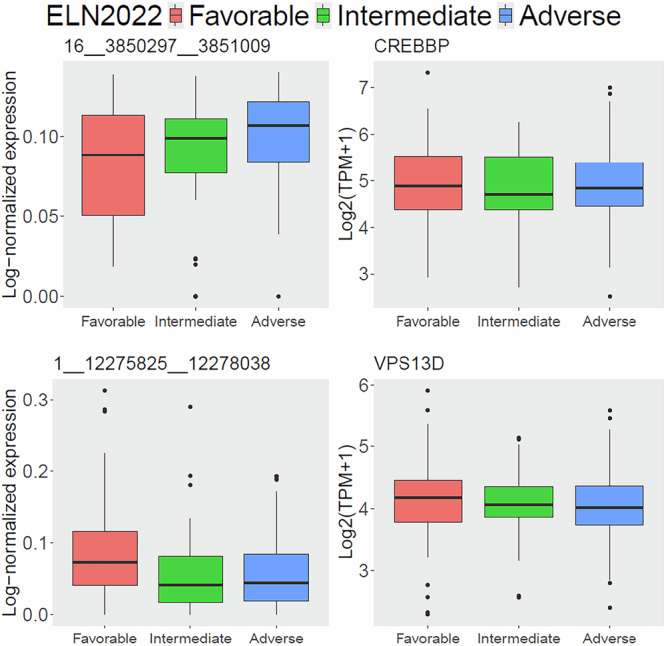
The top ELN2022 subgroup-specific circRNAs. Only Adverse group (top) and Favorable group (bottom) show subgroup-specific circRNAs. The left panels show boxplots of circRNA expression (in FPM) for each ELN2022 group, while the right panels display the corresponding host genes of the circRNAs.

We applied a two-statistic approach^26,27^ to analyze circRNA expression in the ClinSeq-AML cohort and identify subgroup-specific circRNAs for three groups based on the ELN2022 stratification: Adverse, Intermediate, and Favorable. Briefly, we considered a ELN2022 subgroup specific circRNA if 1) its expression was significantly up-regulated in one group, while its expression in the remaining groups showed no statistical difference, and 2) its host gene was not DE in the group. Further details of the two-statistic approach for identifying subgroup-specific circRNAs were provided in the section on Subgroup-specific circRNA discovery in the Methods section. We identified a total of 451 subgroup-specific circRNAs: 358 (79.4%) for the Adverse group, 93 (20.6%) for the Favorable group, and none for the Intermediate group. Examples of the top subgroup-specific circRNAs and their host genes for the Adverse (top) and Favorable (bottom) were shown in Fig. 4. A complete list of all subgroup-specific circRNAs was provided in Supplementary Table S11.

We further investigated subgroup-specific analyses of splice transcripts from the host genes for the top subgroup-specific circRNAs, Supplementary Table S12. One VPS13D transcript (ENST00000489961) exhibited a similar expression pattern, with higher expression in the Favorable subgroup and no statistically significant difference between the Intermediate and Adverse groups (Supplementary Fig. S7), suggesting a potential relation between the transcript and the circRNA for further investigation.

### Prognosis of circRNAs in ELN2022 subgroups

**Fig. 5:**
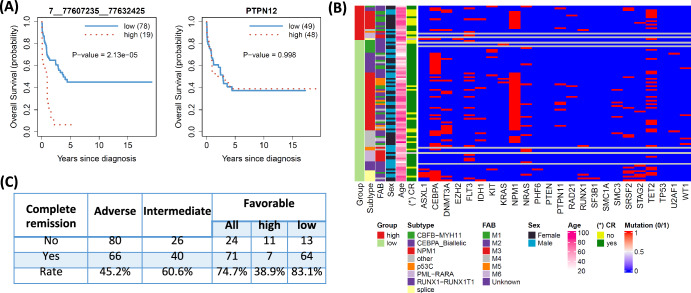
Prognostic circRNAs in ELN2022 subgroups. Kaplan-Meier curves for the significant circRNA identified in survival analysis for the ELN2022 Favorable group (left) and its host gene (right); **C** The association between patient groups based on circRNA expression in (**B**) and various clinical factors, including curated mutations (binary 1/0 if present/not present). Only Complete Remission (CR), marked with asterisks, are statistically significant (FDR < 0.05); **D** the information of CR by ELN2022 groups and the subgroups of the ELN2022 Favorable stratified by the circRNA. expression.

Next, we performed survival analysis to explore the clinical prognostic value of circRNAs for each ELN2022 subgroup. We found one significant circRNA (FDR < 0.2) for the Adverse group: 15__49876278__49879459 (*p* = 6.64×10⁻^6^). For the Intermediate group, two circRNAs were identified as significant: 19__47264603__47264946 (*p* = 3.88 × 10⁻^6^) and 8__73673107__73688813 (*p* = 7.17 × 10⁻^6^). For the Favorable group, we found one significant circRNA, 7__77607235__77632425 (*p* = 2.13 × 10⁻^5^). For the circRNAs from the Adverse and Intermediate groups, AML patients in the high expression group showed better survival (Supplementary Fig. S8). In contrast, for the significant circRNA in the Favorable group, high expression of 7__77607235__77632425 (or *hsa_circ_0080850*) was associated with worse survival (Fig. 5A, left panel). This association remained statistically significant after the adjustment in multivariable Cox regression analysis (Supplementary Table S13). Notably, this association was not significant for its host gene, PTPN12 (Fig. 5A, right panel), as well as its splice transcripts (Supplementary Table S14), suggesting that the result is specific to the circRNA. Moreover, this circRNA showed no statistical significance in survival analysis for the entire AML cohort (n = 315, Supplementary Fig. S9), indicating its specificity to the Favorable group. Gene set enrichment analysis revealed that the two patient groups (high vs low circRNA expression) are associated with multiple AML-related pathways (Supplementary Fig. S10).

We further investigated this circRNA by exploring the association between patient groups and various clinical characteristics of AML patients, as well as 24 curated AML-related mutations (Fig. 5B). Only complete remission (CR) status (*p* = 3.35 × 10⁻^4^, FDR < 0.05) showed strong association with the patient groups. In the ELN2022-favorable group, patients in the high-expression group were generally older (median age 74 for the high group vs. 61.5 for the low group, Supplementary Fig. S11) and were more likely to have no CR (odds ratio = 7.51) after induction treatment (Fig. 5C). Furthermore, the ELN2022-favorable group in this cohort had the highest CR rate (74.7%), compared to 45.2% in the ELN2022-Adverse group and 60.6% in the ELN2022 Intermediate group. However, if the expression of 7__77607235__77632425 in the ELN2022-favorable group patients was high, the CR rate dropped significantly to 38.9% (Fig. 5C).

### Prognosis of circRNAs in large AML molecular subtypes

**Fig. 6:**
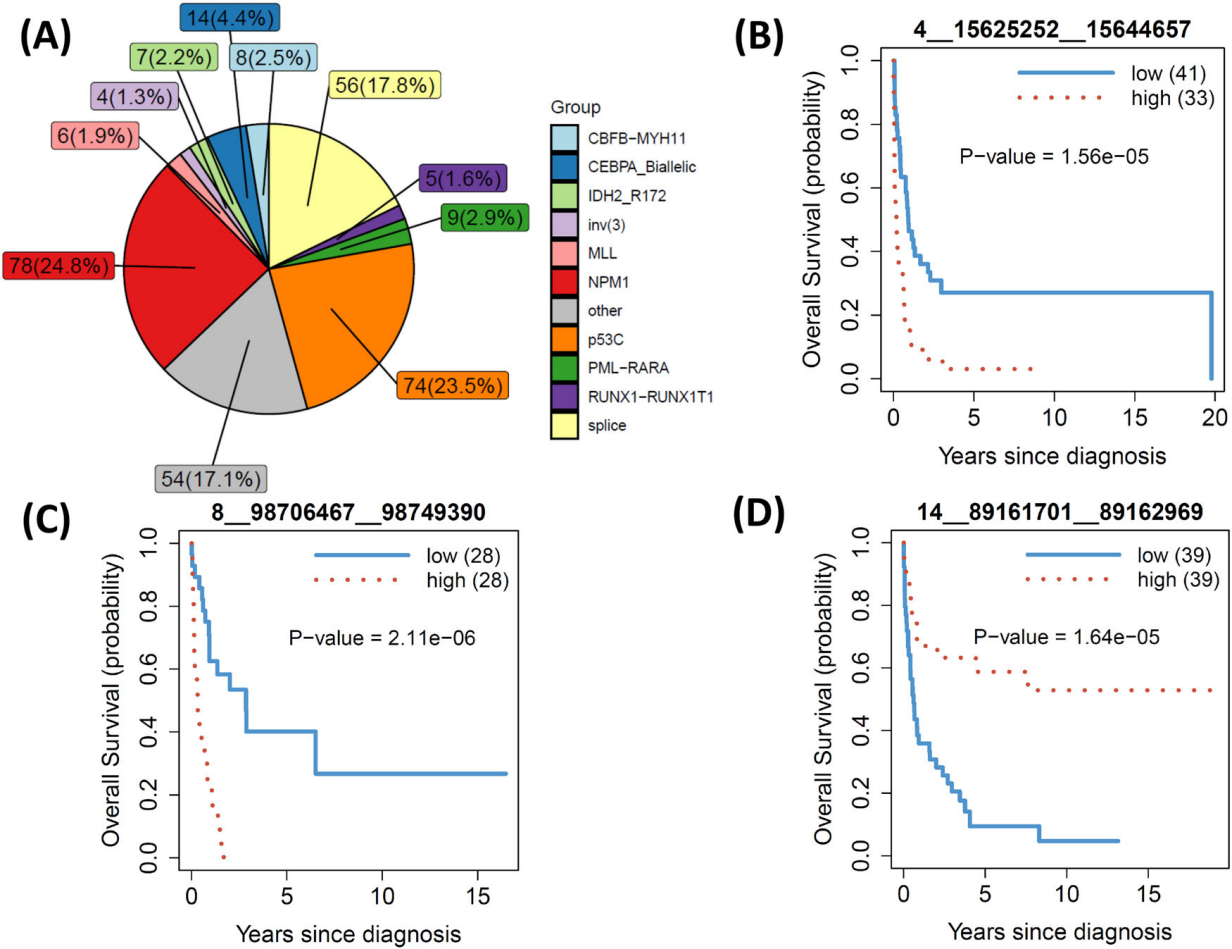
Prognostic circRNAs of three large molecular subtypes. **A** the numbers and proportions of the 11 molecular subtypes in the ClinSeq-AML cohort; **B–D** the Kaplan Meier curves of the significant circRNAs for p53C, Splice, and NPM1 subtypes, respectively.

We next assessed the prognostic relevance of circRNAs within major molecular AML subtypes: NPM1 (*n* = 78), p53C (*n* = 74), and Splice (*n* = 56) (see Fig. 6A), which reflected distinct underlying genomic mechanisms, based on classification of 11 molecular subtypes from a previous study^1^. This analysis complemented the ELN2022-based stratification by evaluating circRNA associations in biologically defined subgroups that cut across clinical risk categories.

We identified three significant circRNAs (FDR < 0.1), one for each subtype (Fig. 6B–D). The associations with their corresponding host genes were not significant for the p53C and Splice subtypes but were weakly significant for the NPM1 subtype (Supplementary Fig. S12). High expression of the circRNAs identified from the p53C (4__15625252__15644657, host gene FBXL5, p = 1.56 × 10⁻⁵, FDR = 0.09) and Splice (8__98706467__98749390 or *hsa_circ_0085045*, host gene STK3, p = 2.11 × 10⁻⁶, FDR = 0.01) subtypes were associated with poorer patient survival, Fig. 6B, C. In contrast, for the NPM1 group, we identified 14__89161701__89162969 or *hsa_circ_0000558* (p = 1.64 × 10⁻⁵, host gene FOXN3, FDR = 0.09), which was associated with better survival for patients with high expression of the circRNA, Fig. 6D. When comparing the results with the analysis of the same circRNAs using all 315 samples, we observed significant enrichment of the association for two molecular subtypes, Splice and NPM1. Notably, there was no significant association between low and high expression of 4__15625252__15644657 in the p53C subtype (Supplementary Fig. S13).

The analysis of clinical features and the status of the 24 curated mutations showed that all circRNAs were significantly associated with CR, with p-values of 0.004, 0.01, and 0.02 for the NPM1, p53C, and Splice subtypes, respectively, Supplementary Fig. S14. For the NPM1 subtype, 14__89161701__89162969 was also associated with the FLT3 mutation, where the high-expression group tended to lack the FLT3 mutation, in contrast to the low-expression group (*p* = 0.02, OR = 3.24, CI: 1.17-9.45). This finding aligned with the known fact that FLT3 mutations were associated with worse survival in NPM1 patients. For the Splice molecular subtype, 8__98706467__98749390 was associated with age (*p* = 0.02) and U2AF1 mutation (*p* = 0.03). The high-expression group in the Splice subtype tended to have older patients (median age 74.5 in the high group vs. 67.5 in the low group), and all patients with U2AF1 mutations (*n* = 6, 10.7%) belong to this group. It is noteworthy that U2AF1 mutation was a relatively rare event, found in only 14 patients (4%) across all 315 AML patients in the ClinSeq-AML cohort.

## Discussions

This study provided a comprehensive landscape of circRNAs in AML, using data from the large ClinSeq-AML cohort. By identifying over 5700 high-confidence circRNAs across 315 AML patients, we have expanded the understanding of circRNA diversity and their association with ELN2022 stratifications, AML molecular subtypes, clinical features, and drug responses.

Key findings included the identification of DE circRNAs between AML patients and healthy HSPCs and MHPs, as well as prognostic circRNAs associated with patient survival. Notably, circRNAs such as 11__94583448__94608 and 8__67118248__67137603 exhibited specific survival benefits for patients with higher expression levels, independent of their host gene expression. These findings underlined the potential of circRNAs as biomarkers that could complement traditional genomic and transcriptomic approaches in AML prognosis. Furthermore, the association of 11__94583448__94608 and 8__67118248__67137603 with responses to drugs like Rucaparib and Lenalidomide, validated in independent cohorts (Beat-AML and FIMM-AML), underscored the potential of circRNAs in guiding precision medicine strategies. These insights not only highlighted the biological relevance of circRNAs but also paved the way for their integration into clinical practice as biomarkers for therapy selection and monitoring.

The subgroup-specific analysis of circRNAs also revealed unique profiles associated with ELN2022 stratification. Specifically, we identified 358 and 93 subgroup-specific circRNAs in the Adverse and Favorable groups, respectively, suggesting that these circRNAs may help distinguish between prognostic categories in AML. Furthermore, we identified the prognostic circRNA 7__77607235__77632425, specific to the ELN2022 Favorable group, was associated with significantly reduced CR rates and older age, suggesting its potential role in identifying patients within this group who may require alternative therapeutic strategies. Similarly, circRNAs linked to molecular subtypes such as p53C and splice variants further demonstrated prognostic significance, reflecting the unique molecular pathways driving AML pathogenesis in these subtypes.

Despite these advances, several limitations of this study should be acknowledged. First, reliance on RNA-seq datasets prepared with rRNA-depletion protocols limits direct comparisons with larger cohorts such as Beat-AML and TCGA-AML, which predominantly use poly (A) selection methods. In addition, experimental validation of circRNAs using approaches such as RNase R treatment followed by junction-spanning RT-qPCR is not performed. Moreover, the associations between circRNA expression and drug sensitivity metrics (DSS/AUC) reported in this study are correlative and do not establish causality.

Future studies should validate key prognostic circRNAs in AML cell lines and primary patient samples and investigate their functional roles in leukemogenesis and treatment response. For example, collecting additional RNA-seq data from AML cohorts using rRNA-depleted protocols would also enable broader validation and circRNA detection. Functional validation, such as perturbing circRNA expression in AML cell lines followed by drug response assays, or evaluating in prospective clinical cohorts with well-annotated treatment outcomes (e.g., for agents such as lenalidomide or rucaparib), will be required to confirm their predictive and mechanistic relevance.

Another limitation relates to the use of healthy HSPCs as controls for differential expression (DE) analysis. Because AML samples may contain a greater proportion of differentiated cells than HSPCs, such a comparison may capture differences driven by cellular differentiation rather than disease-specific effects. Although there is partial concordance between the DE results obtained using healthy HSPCs and healthy mature hematopoietic populations, and gene set enrichment analysis highlights AML-relevant pathways rather than cellular differentiation-related pathways, the DE results should nevertheless be interpreted with caution. Furthermore, the relatively small sample sizes of the healthy comparison groups limit the statistical power to detect DE circRNAs; this limitation could be addressed in future studies by including larger numbers of healthy samples.

Finally, while the identified circRNAs demonstrate prognostic and therapeutic relevance, functional studies are required to elucidate their roles in AML biology, particularly their interactions with miRNAs, RNA-binding proteins, and other molecular pathways. These investigations, while crucial, fall outside the scope of this study. Future research is needed to integrate circRNA profiles with multiomics data to further unravel their regulatory networks and functional implications in AML.

In conclusion, this study presents a detailed landscape of circRNAs in AML, demonstrating their potential as biomarkers and therapeutic targets. These findings provide new insights into the potential role of circRNAs in AML and may inform future studies exploring their mechanisms and their potential utility in personalized approaches to diagnosis and treatment.

## Methods

### Datasets

The circRNA detection methods described in the section on Circular RNA detection tools in the Methods section were applied to several AML cohorts to identify circRNAs. RNA-seq data from two AML cohorts, ClinSeq-AML^19^ and Lux et al. study (Lux-AML cohort)^11^, were generated using rRNA-depleted protocols, which can be used for circRNA detection. In contrast, the Beat-AML cohort^16,17^ contained polyA-selected RNA sequencing data, which was not expected to detect circRNAs and thus served as a negative control for the circRNA detection methods.

Gene expression read counts from the RNA-seq samples in the ClinSeq-AML and Lux-AML cohorts were generated using Salmon^28^ version 1.4.0 with Ensembl Homo sapiens (hg38) annotation (version GRCh38.106). Normalized gene expression, in counts per million (CPM), was calculated using the cpm() function of edgeR, employing the TMM method for library size normalization^29^.

#### - ClinSeq-AML cohort

This dataset included 315 patients diagnosed with AML in Sweden between February 1997 and August 2014^19^. Transcriptomic RNA sequencing (RNA-seq) was conducted using the Illumina HiSeq-2500 platform with paired-end 101-bp reads, and ribosomal RNA (rRNA) depletion was performed during library preparation using the Ribo-Zero gold kit. The drug-sensitivity data included information on the response of 45 patients to 528 drugs, measured using the drug-sensitivity score (DSS)^25^. Further details of the sequencing data and cohort can be found in the original study^19^. This cohort was used as the discovery dataset in this study.

#### - Lux-AML cohort and healthy control data

This cohort included RNA-seq samples from 61 AML patients and 16 healthy CD34+ hematopoietic stem and progenitor cells (HSPCs)^11^. RNA sequencing libraries were prepared using an rRNA-depletion protocol (the TruSeq Stranded Total RNA Kit with Ribo-Zero Human of Illumina), followed by sequencing on the Illumina HiSeq 2000 platform with a 100-cycle paired-end protocol. This cohort was used to identify circRNAs in AML patients and to validate the results. In addition to the 16 healthy HSPC samples, we included six RNA-seq samples from healthy mature hematopoietic populations (MHPs) derived from donor peripheral blood for analysis^24^. These samples were generated using a similar rRNA-depletion protocol^24^. Because HSPCs might lack differentiated hematopoietic cell populations, MHPs provided a complementary control set, enabling comparison and validation of results from healthy HSPC samples.

#### - FIMM-AML cohort

This cohort^30^ includes multiomics profiling and ex vivo drug response data from 252 samples of 186 AML patients in Finland. The drug sensitivity data covered 515 drugs across 183 patient samples, with responses measured by drug sensitivity score (DSS), as used in the ClinSeq-AML cohort. The read count gene expression matrix (hg38) for 163 AML samples was available in the supplementary data of the cohort study^30^. This cohort was used to validate the results of the drug sensitivity analysis.

#### - Beat-AML cohort

The Beat-AML project^16,17^ included comprehensive genomic and transcriptomic analyses, clinical annotations, and drug response profiles of AML patients. RNA sequencing was performed using the Agilent SureSelect Strand-Specific RNA Library Preparation Kit on the Agilent Bravo robot. Sequencing was carried out on the Illumina HiSeq 2500 platform with a 100-cycle paired-end protocol. The read count gene expression matrix (hg38) for this dataset can be downloaded from https://portal.gdc.cancer.gov. This cohort included samples from 442 AML patients collected at initial diagnosis, along with 34,802 records of drug sensitivity data from 338 patients across 165 drugs. Drug response was measured using both IC50 and area under the curve (AUC) metrics. Additional details about the dataset were available in the original studies^16,17^. This cohort was used to identify negative controls for circRNA detection and to validate the results of drug sensitivity analysis.

### Circular RNA detection tools

Three circRNA detection methods, including Circall^20^, CIRI2^21^, and find_circ2^22^ were used to identify circRNAs from RNA-seq data, as they were widely adopted and top-performing tools in several benchmarking analyses^20,31–33^. All tools were applied with default parameter settings to the fastq files of RNA-seq samples, using the Ensembl Homo sapiens (hg38) annotation version GRCh38.106. For downstream analysis, we retained circRNAs identified by all three tools. The circRNA expression across the three tools was highly concordant (see Supplementary Fig. S1). The results of Circall, which provided both BSJ read counts and normalized expression data for circRNAs, were used for further analysis. The tool used the junction fragment per million (FPM), adjusting for read depth normalization of the supporting reads. Additionally, we adopted the normID format for naming circRNAs, as recommended by Circall^20^ and CircNetVis^34^. The normID was a concatenation of the chromosome name (chr), the start position, and the end position, formatted as chr__start__end, based on the one-based coordinate system of the genome reference.

### True negative circRNAs

In this study, we constructed the true negative (TN) set using circRNAs identified from RNA-seq samples of the Beat-AML cohort. Since circRNAs lacked poly-A tails, and the RNA-seq data of the Beat-AML cohort were generated using a poly-A library preparation protocol, they were expected not to contain circRNAs. Therefore, the circRNAs identified in this cohort with high confidence were likely true negatives. Specifically, we collected circRNAs with BSJ read support greater than 4 in more than 25% of patients. In total, we identified 339 potential TN circRNAs across the three detection tools, which were listed in Supplementary Table S1.

### Subgroup-specific circRNA discovery

For subgroup-specific circRNA identification, we applied the two-statistic approach from previous studies^26,27^. This method involved two statistics: a robust t-test (T1) and a chi-square test (T2). T1 was used to determine whether a given group significantly differed from all other groups, while T2 assessed whether the expression levels were similar among the remaining groups. A circRNA was considered subgroup-specific if T1 was large, indicating a significant difference from other groups, and T2 was small, suggesting no significant differences among the other groups. Further details of the method can be found in the original studies^26,27^. In this study, we considered an ELN2022 subgroup-specific circRNA if 1) FDR < 0.01 for T1 and *p* > 0.05 for T2, and 2) its host gene was not DE in that group in comparison to the remaining patients (*p* > 0.05).

### Validation of drug sensitivity analysis

We applied the approach proposed in a recent study^12^ to identify low-like and high-like patient groups in the Beat-AML and FIMM-AML cohorts. Briefly, we performed DE analysis using the gene expression data from two groups (low vs. high) stratified by the expression of a prognostic circRNA in the ClinSeq-AML cohort. We then selected a set of *k* DE genes that meet the following criteria: FDR < 0.05, absolute log2 fold-change > 1, and mean expression higher than the median (of mean expression) across all genes. This gene set was used to classify patients into low-like and high-like groups in the validation cohorts as follows. For each DE gene in the gene set, patients were assigned to the low-like or high-like group based on its expression (log2 count-per-million) in the validation cohort. Each patient thus received *k* group labels (one for each DE gene), and the final group assignment was determined by a majority vote (i.e., > 50% of votes). One of the advantages of this approach was that it could diminish the batch effects of gene expression between different cohorts.

### Data analyses

DE analysis was conducted on the circRNA count matrix using DESeq2^35^. We also applied ComBat-seq^36^ for batch effect adjustment between ClinSeq-AML, Lux-AML, and healthy MHP datasets. Gene set enrichment analysis (GSEA) was performed using GSEA software^37^. Welch’s t-test was applied to the normalized expression and drug sensitivity data for comparison between the two sample groups. The association between patient groups and clinical categorical features was assessed using the chi-squared test. To compare the survival between the low and high groups, stratified by the median expression of the prognostic circRNAs, we used the Kaplan-Meier estimator and log-rank test. Multivariable survival analysis was performed using Cox proportional hazards regression model with adjustments for age, sex, key mutations including FLT3, NPM1, TP53, key chromosome rearrangement including CBFB-MYH11, PML-RARA, RUNX1-RUNX1T1, MLL rearrangement, GATA2-MECOM (inv 3), and ELN2022. P-values were obtained from these tests, and the false discovery rate (FDR)^38^ was calculated using the Benjamini & Hochberg (BH) method. All data analyses and visualizations were performed using R software version 4.3.0.

## Supplementary information

Supplementary_document_final

## Data Availability

The all public datasets were available from their original studies: dbGaP accession ID phs001657.v1.p1 for the BeatAML cohort^16,17^ and GEO accession ID GSE158596 for the Lux-AML cohort^11^. For the FIMM-AML cohort^30^, the data are provided by its study and are available at Zenodo repository (https://zenodo.org/records/7274740). The demographic information and somatic mutations of the ClinSeq-AML cohort^19^ are publicly available in the ClinSeq-AML repository at Zenodo 10.5281/zenodo.292986. All circRNAs and result data of the ClinSeq-AML cohort are provided in the supplementary documents. For further details about the ClinSeq-AML drug data, please contact the corresponding author.
