# Supplementary material for: Landscape of circular RNAs in acute myeloid leukemia and their clinical significance
Source: NPJ Precis Oncol. 2026 Mar 5;10:116. doi: 10.1038/s41698-026-01357-6 (PMC12996449; doi:10.1038/s41698-026-01357-6)
Supplement: Supplementary file 1 — Supplementary_document_final [file 41698_2026_1357_MOESM1_ESM.pdf]

Supplementary documents for

Landscape of Circular RNAs in Acute Myeloid Leukemia and their clinical significance

Nguyen et al.

Supplementary Figures

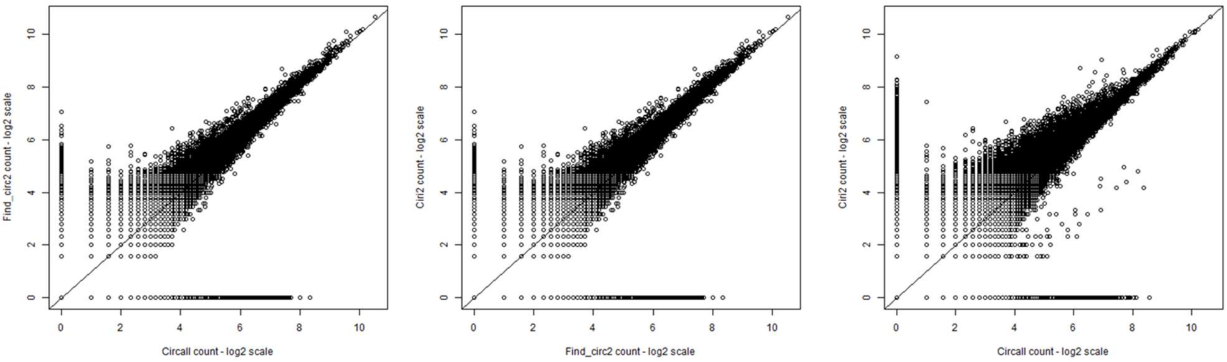

Figure S1. Concordance of circRNA expression between Circall, Find_circ2 and CIRI2. Each point presents expression of a single circRNA.

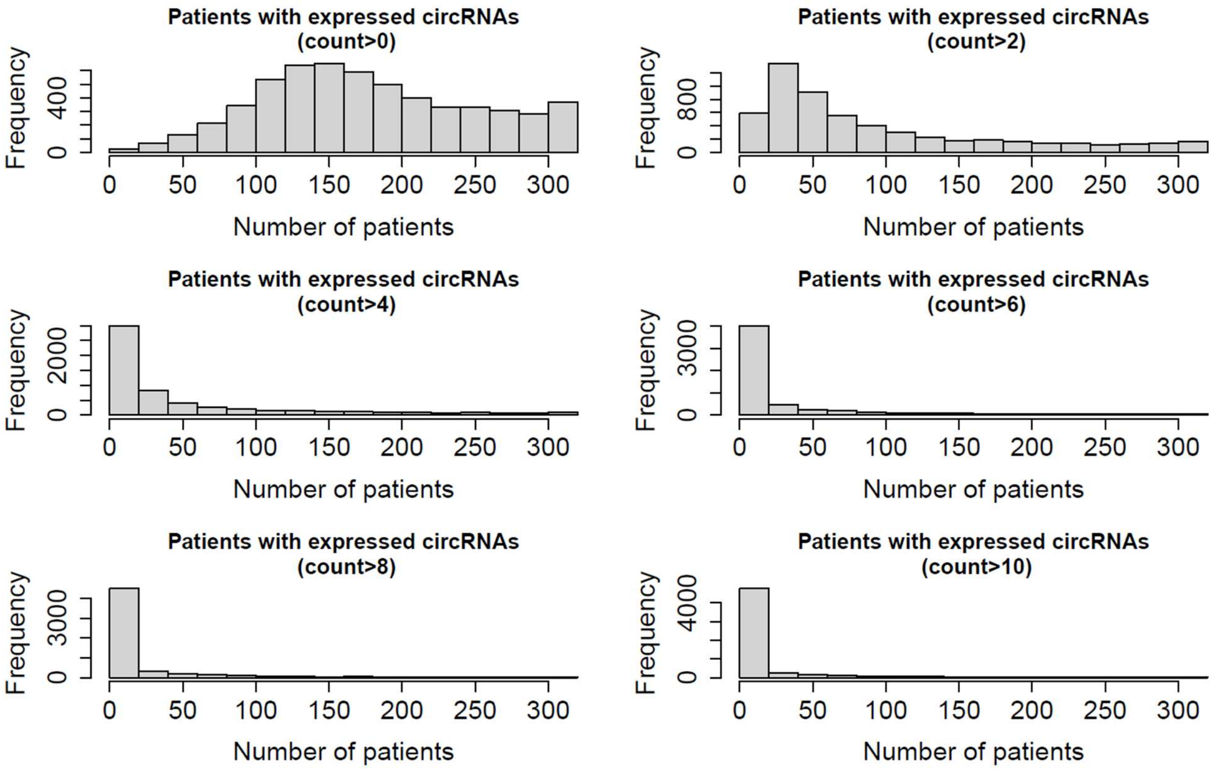

Figure S2. Distribution of the number of patients for expressed circRNAs with different expression thresholds for circRNAs.

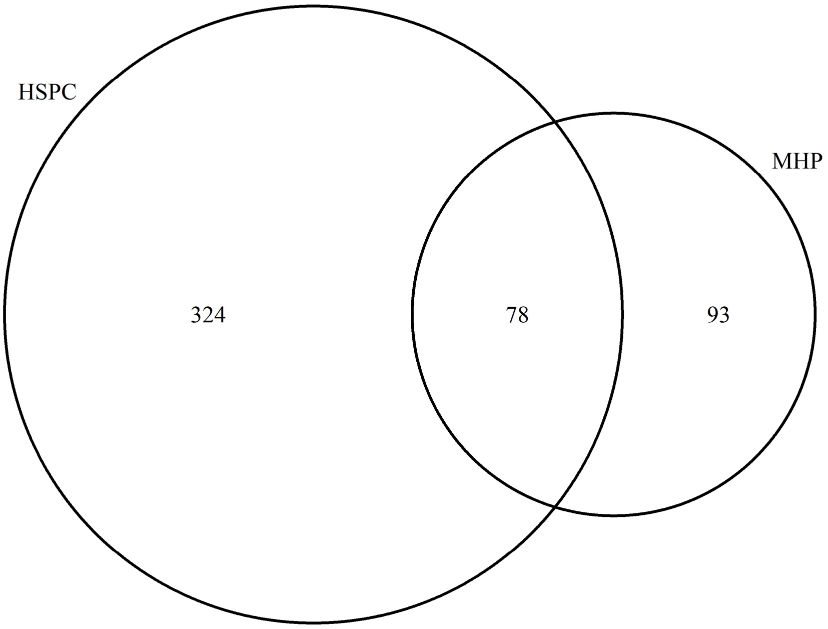

Figure S3. Comparison between DE circRNAs identified in two healthy control datasets. Two healthy datasets include 16 samples of healthy CD34+ hematopoietic stem and progenitor cells (HSPCs) and 6 samples of healthy mature hematopoietic populations (MHPs) derived from donor peripheral blood.

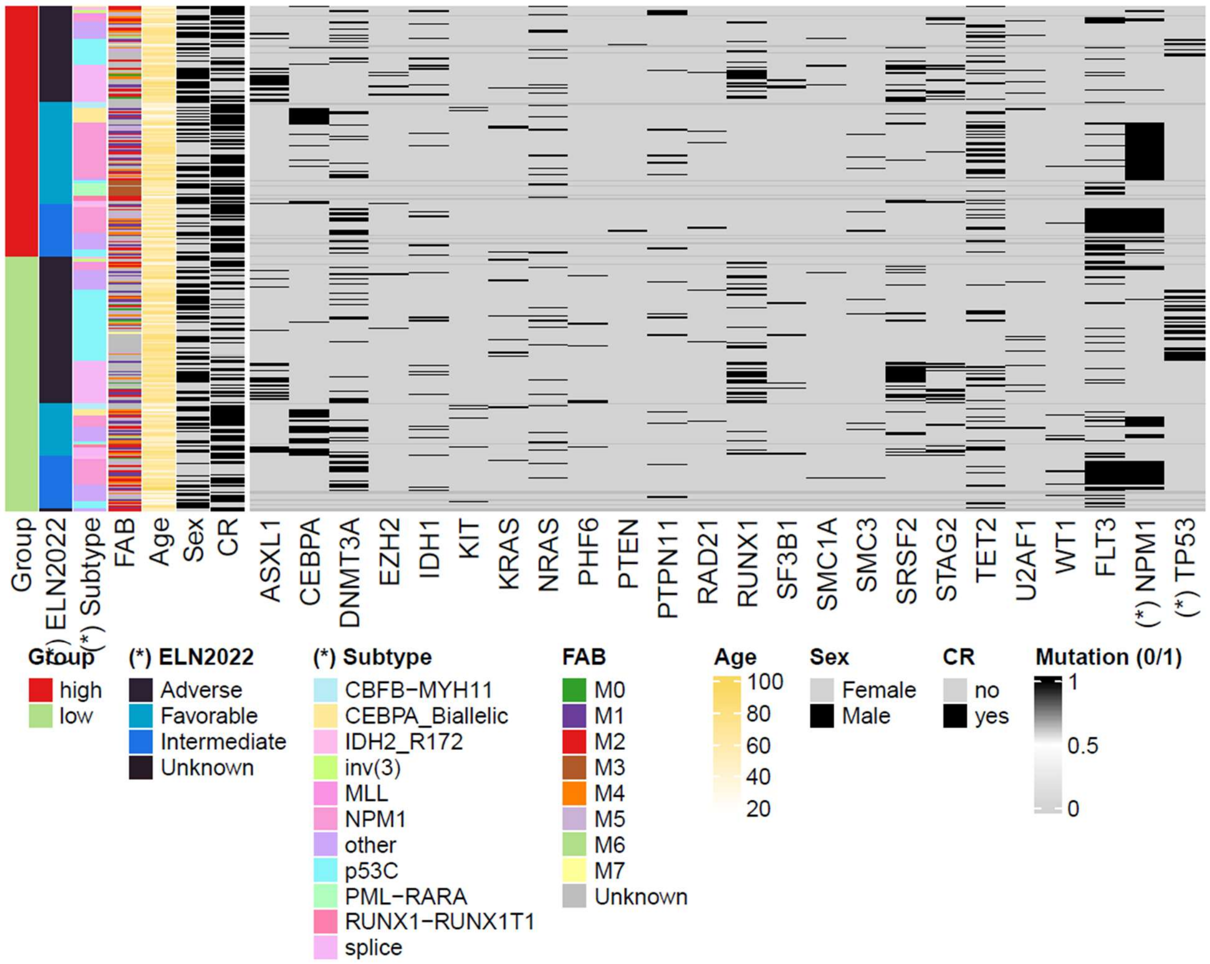

Figure S4. Heatmap of 11__94583448__94608 group and key clinical and genomic characteristics.

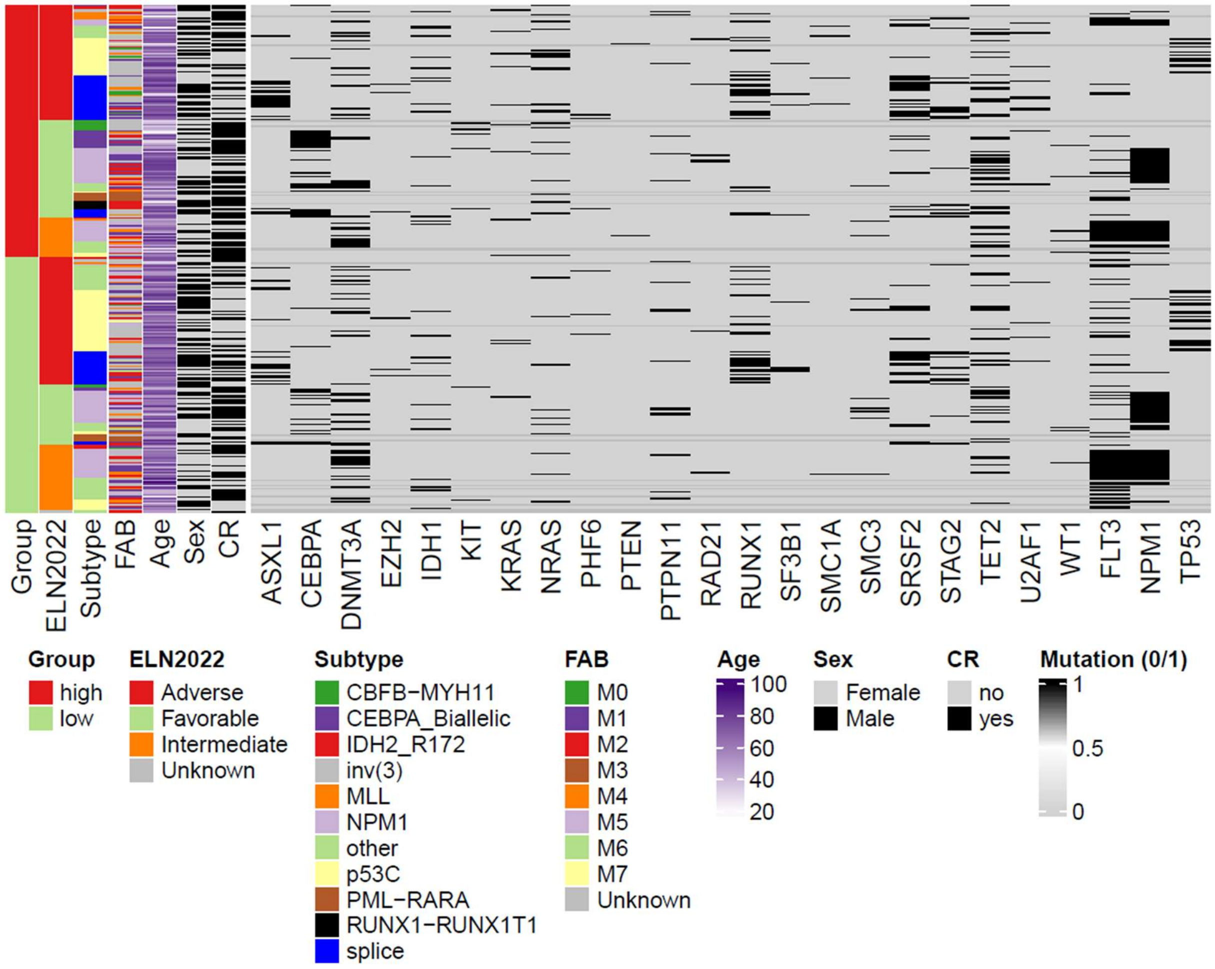

Figure S5. Heatmap of 8__67118248__67137603 group and key clinical and genomic characteristics.

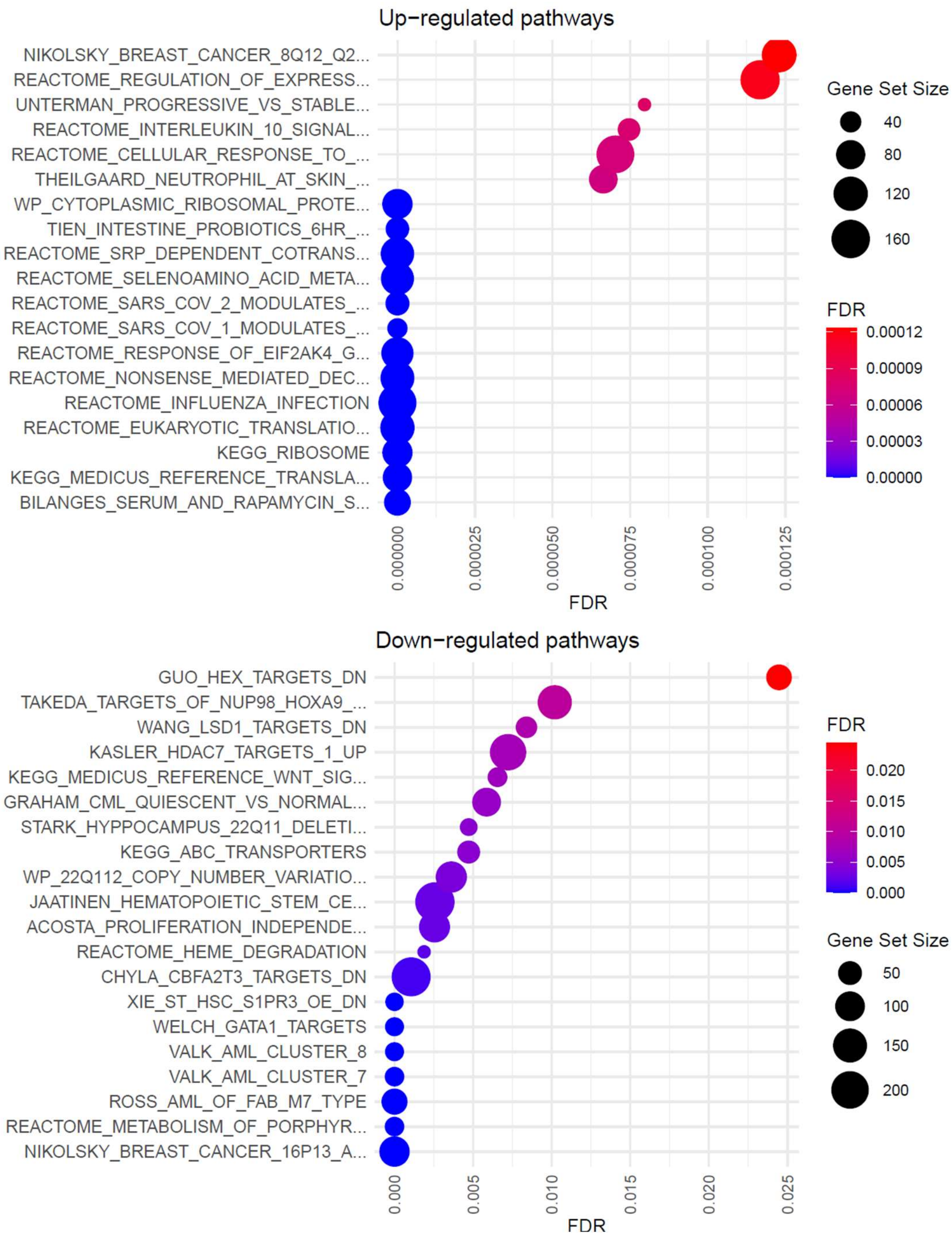

Figure S6. Gene set enrichment analysis for 8__67118248__67137603

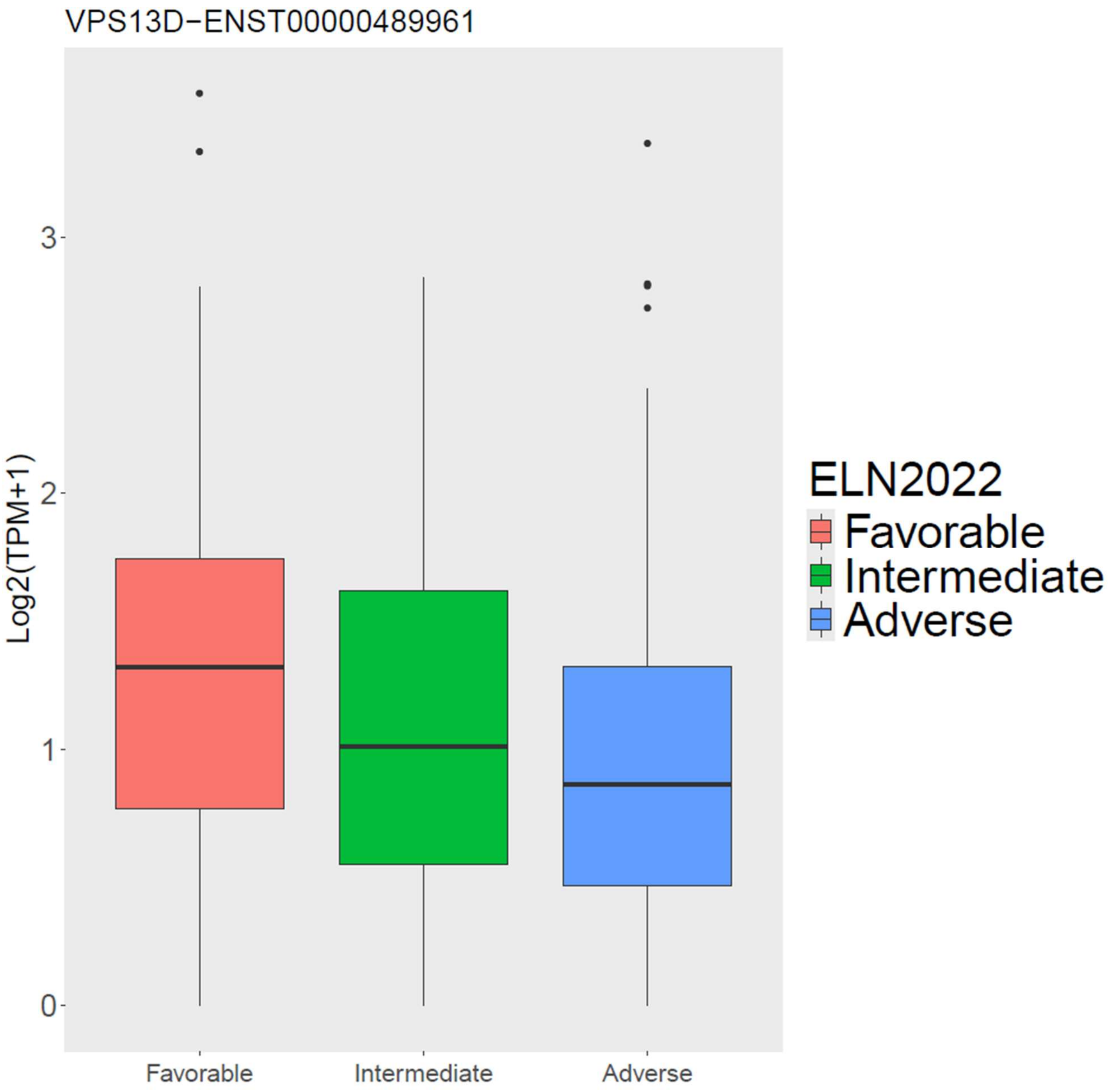

Figure S7. Distribution of VPS13D transcript (ENST00000489961) from the top ELN2022-favorable specific circRNA (1__12275825__12278038).

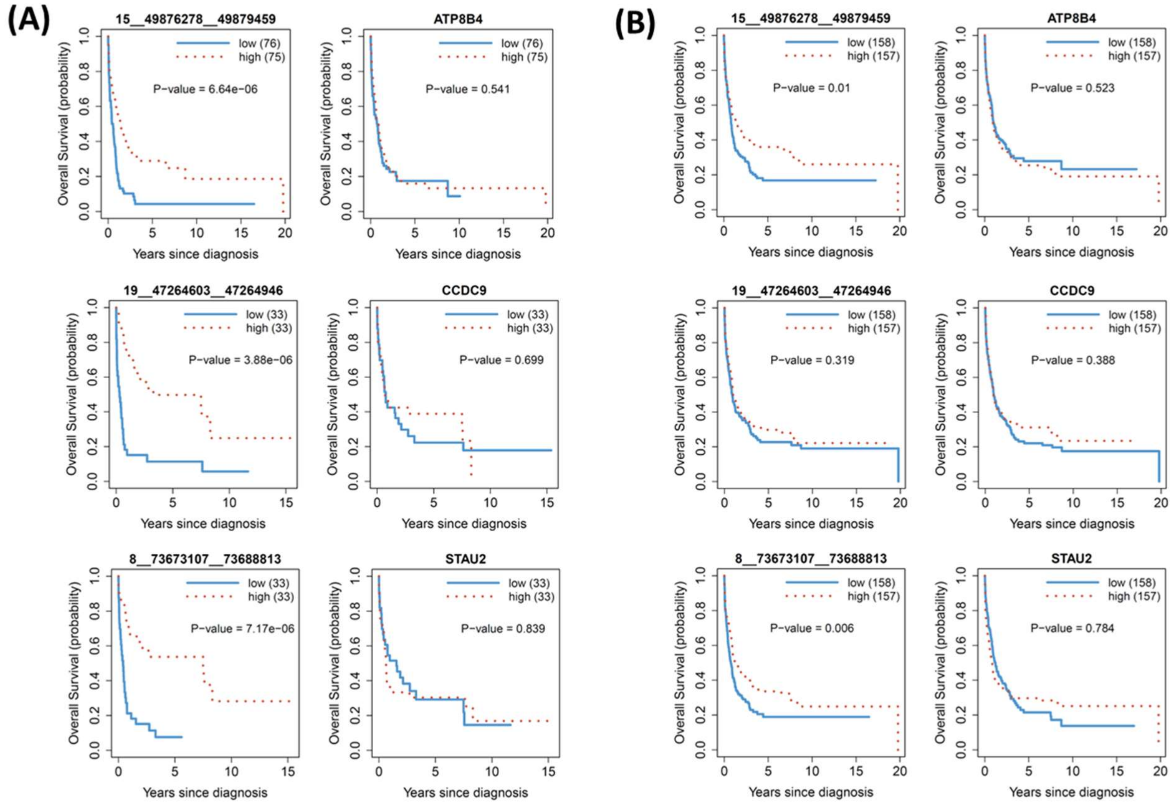

Figure S8. Results of survival analysis for the ELN2022 Adverse and Intermediate groups. A) The Kaplan Meier curves of the significant circRNAs (left) and their host genes (right) using the patients in the ELN2022 groups including Adverse (top row) and Intermediate (the other rows). B) The similar survival analysis of the same significant circRNAs in panel A but using all n=315 patients from the dataset.

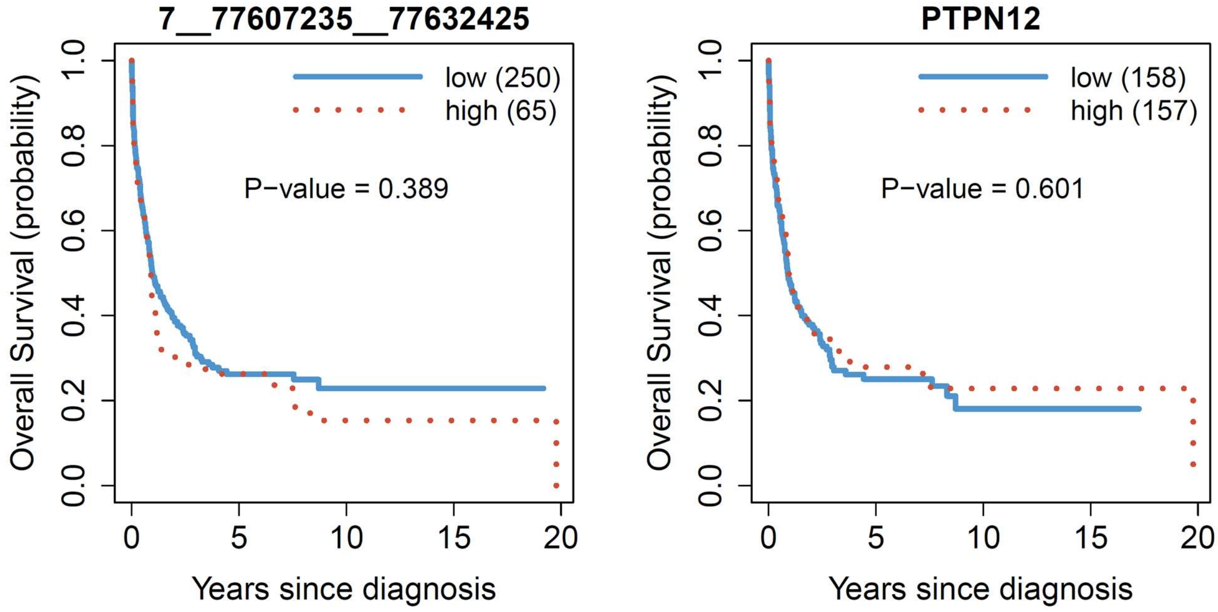

Figure S9. The Kaplan Meier curves of the significant circRNAs by survival analysis for the ELN2022 Favorable group (left) and their host genes (right) for n=315 patients of the whole dataset.

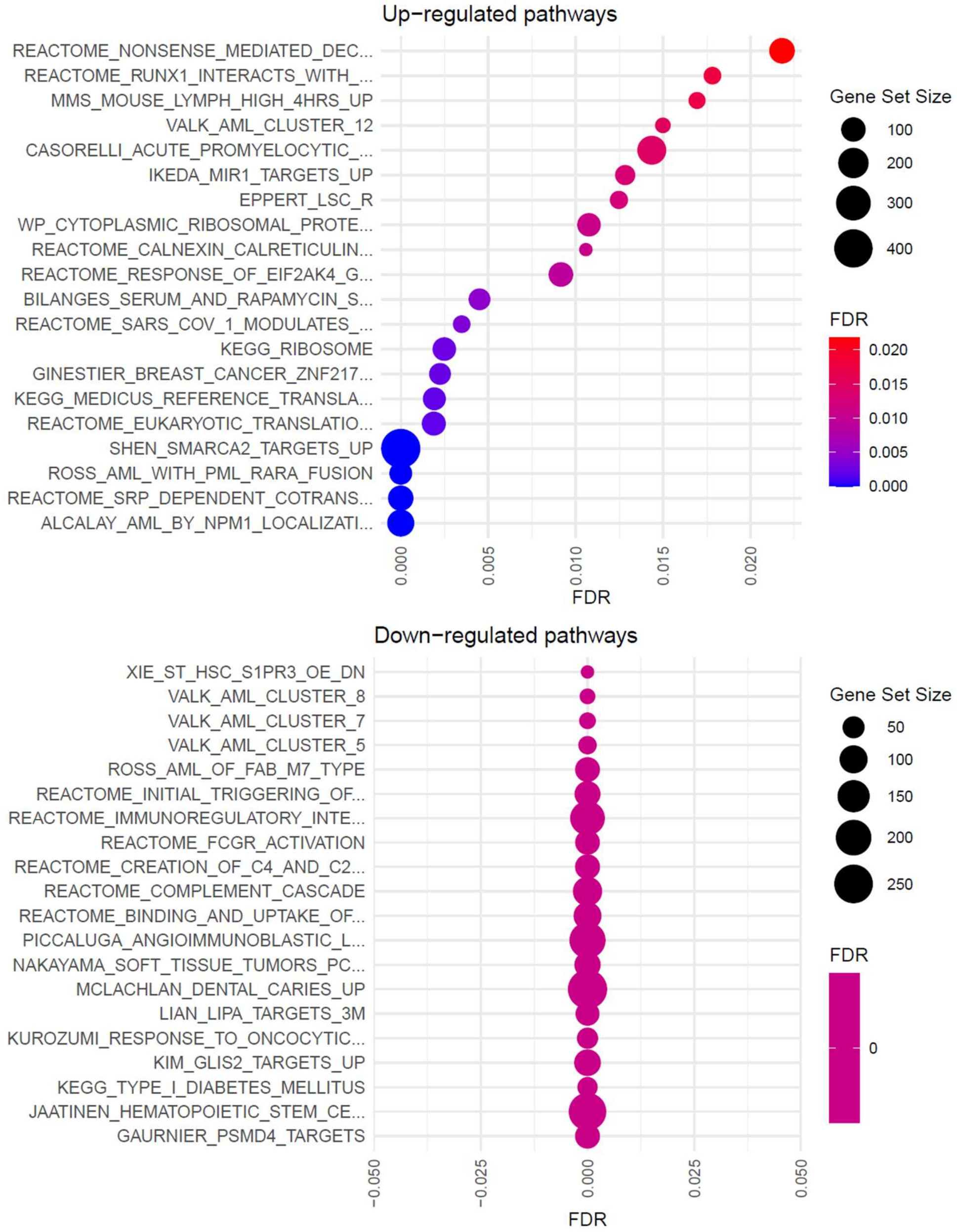

Figure S10. Gene set enrichment analysis for 7__77607235__77632425.

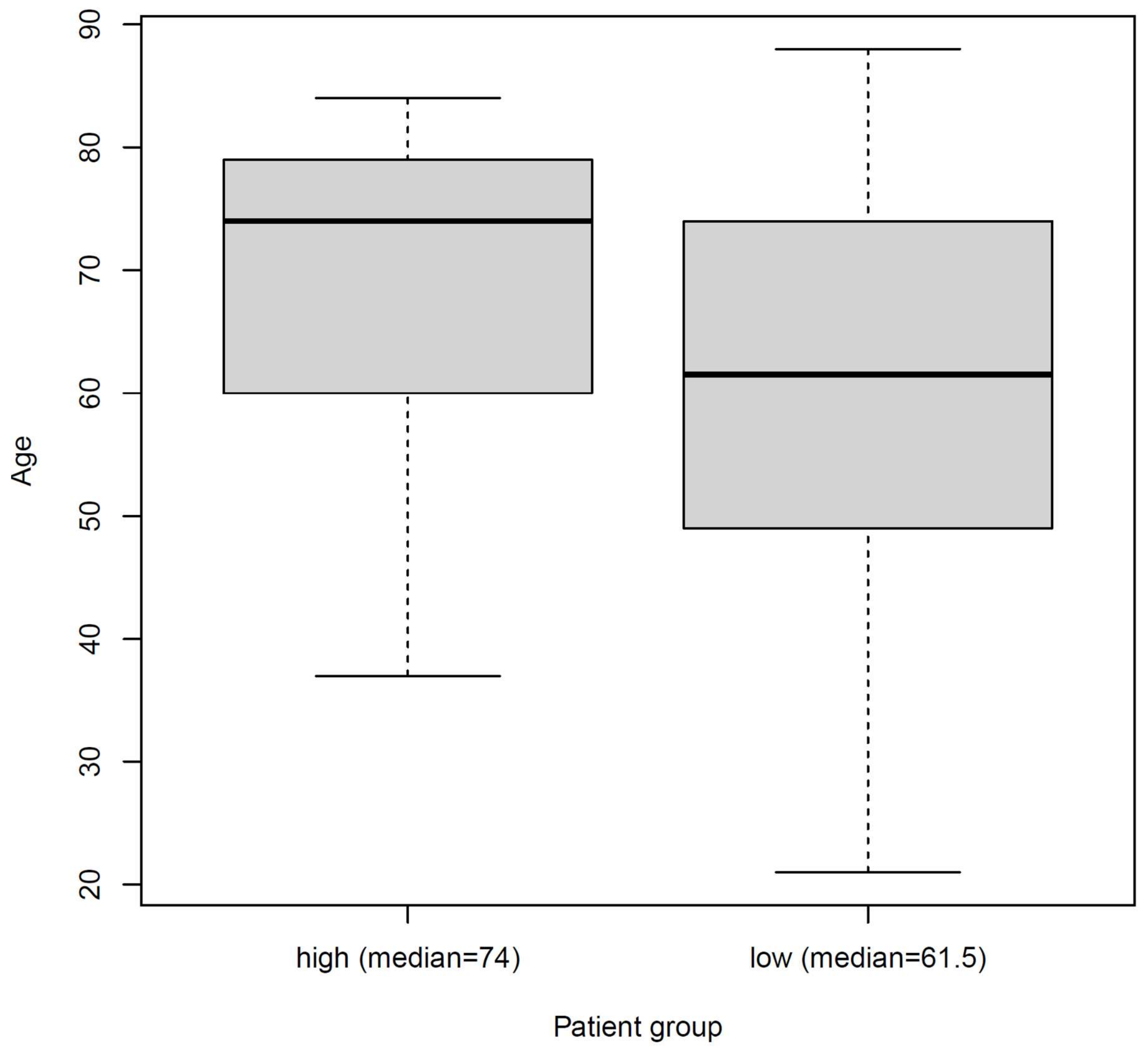

Figure S11. Distribution of age in patient groups by median expression of 7__77607235__77632425.

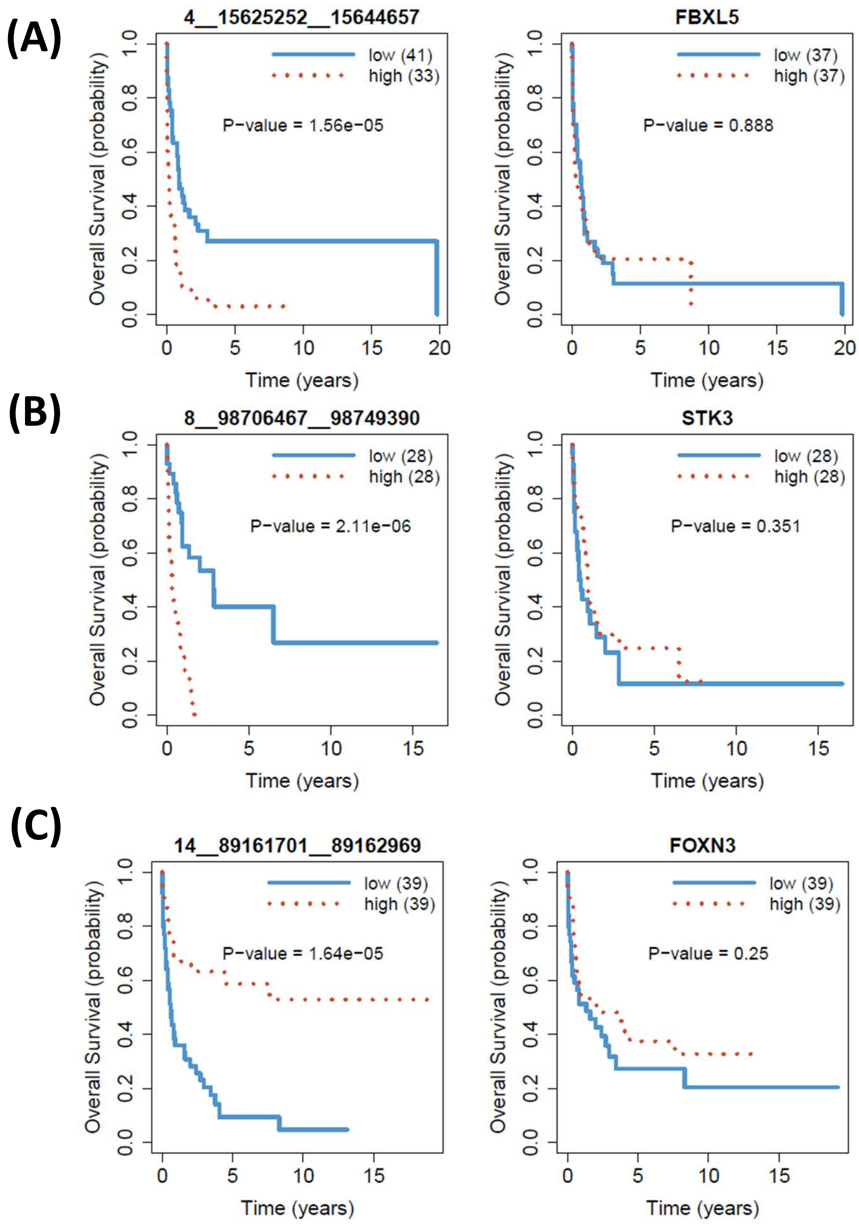

Figure S12. Panels A, B and C are the Kaplan Meier curves of the significant circRNAs (left) and their host genes (right) for p53C, Splice, and NPM1 subtypes, respectively

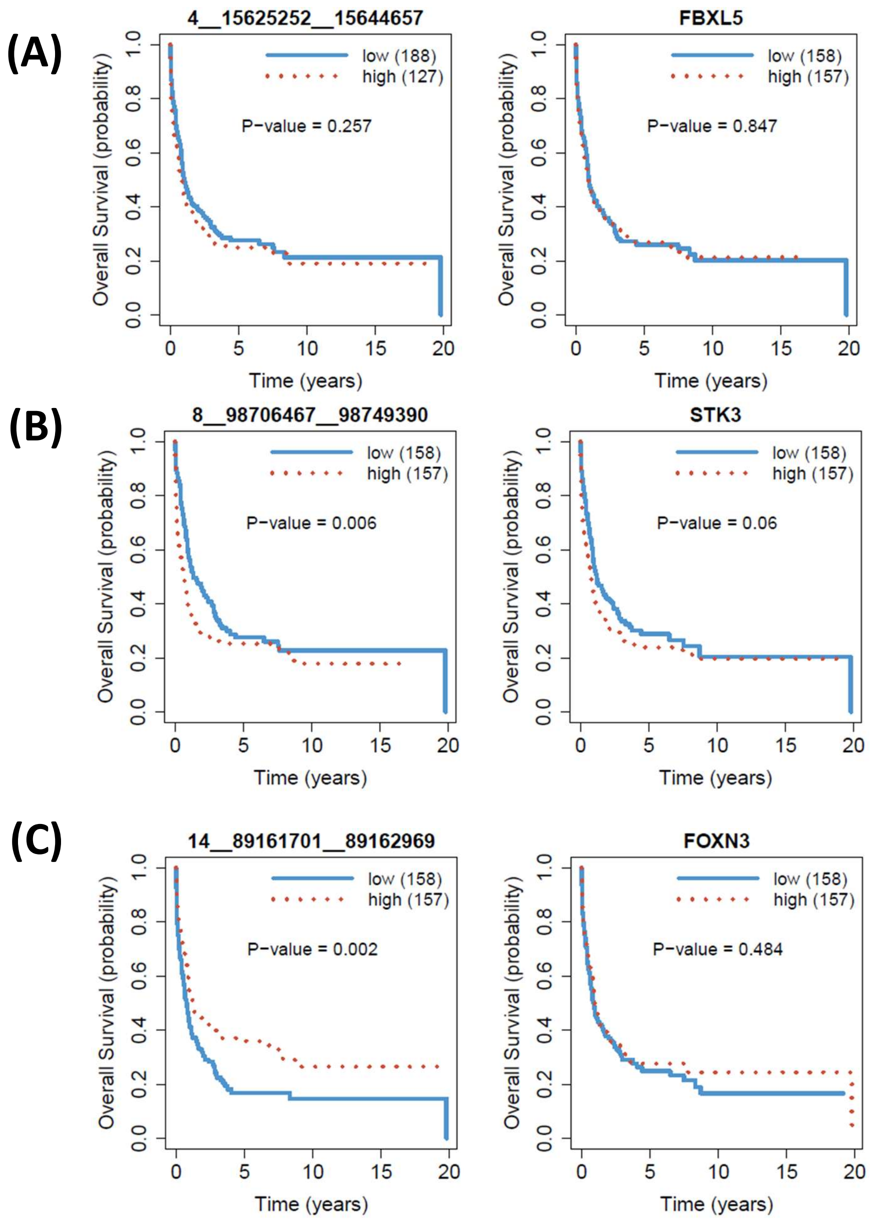

Figure S13. Panels A, B and C are the Kaplan Meier curves of the significant circRNAs (left) and their host genes (right) but applying for all 315 samples of the cohort for p53C, Splice, and NPM1, respectively.

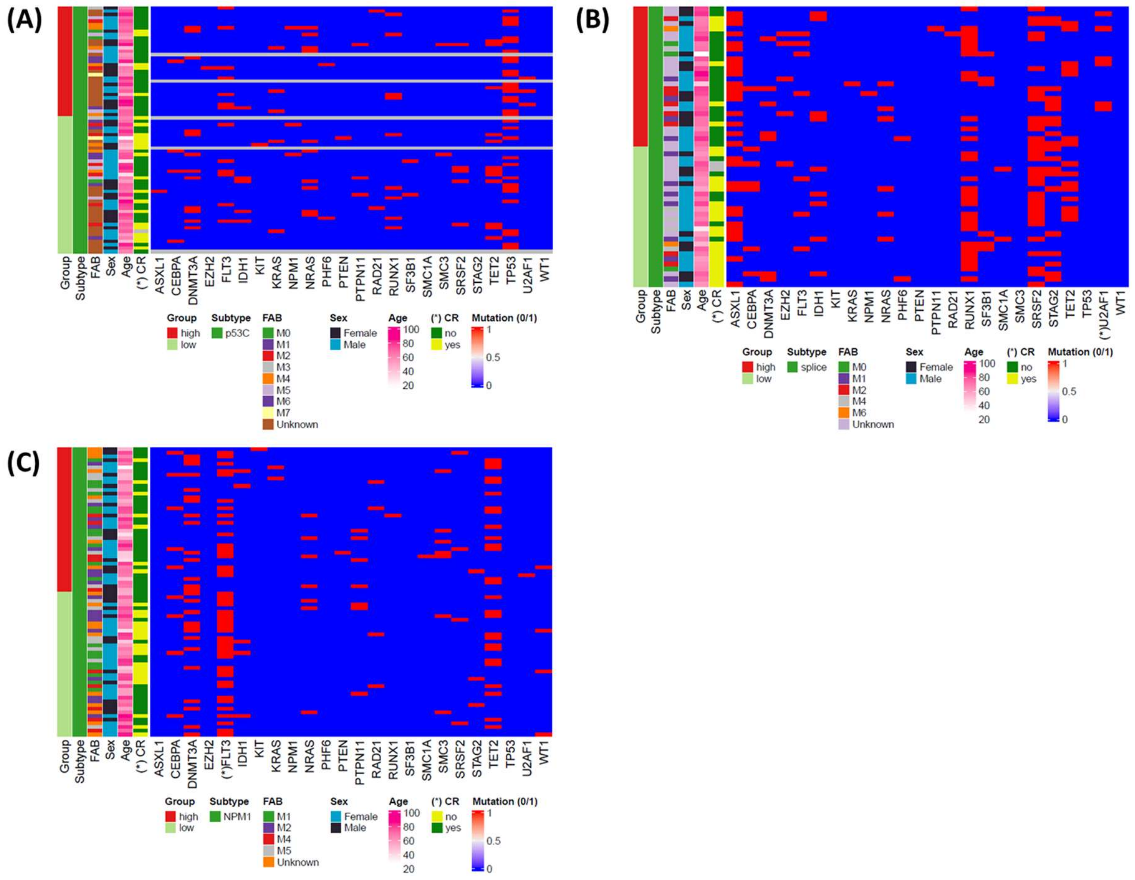

Figure S14. Panels A, B and C are the association between patient groups based on significant circRNAs and various clinical factors, including curated mutations (binary 1/0 if present/not present) for p53C, Splice, and NPM1, respectively.

Supplementary Tables

Supplementary Table S1. The list of 339 true negative circRNAs

Index	CircRNA_ID
1	11__116775503__116779059
2	11__131467__133778
3	11__131467__134796
4	12__103947311__103947430
5	13__45377061__45377215
6	13__45378611__45379274
7	13__45379195__45379335
8	13__45379926__45380001
9	13__45383091__45383235
10	13__50943321__50943394
11	14__105770293__105770337
12	14__35110525__35110629
13	15__64499293__64500166
14	16__18418392__18500567
15	16__18424274__18501083
16	16__18426630__18506992
17	16__18429010__18509267
18	16__18430815__18511656
19	16__18431807__18513433
20	16__18433348__18514483
21	16__18434654__18516010
22	16__2759569__2759661
23	16__72056530__72057466
24	16__72057392__72058355
25	16__90162597__90163958
26	17__44351550__44352248
27	17__45513185__45519518
28	17__51161160__51168343
29	17__83084938__83085323
30	18__12346779__12348383
31	19__201756__205076
32	19__202041__205227
33	19__23358430__23362725
34	19__49854970__49858114
35	19__52873992__52888253
36	1__117402186__117420649
37	1__120438544__120443050
38	1__120808545__120813993
39	1__146118370__146122391
40	1__146966464__146971394
41	1__146968448__146972960
42	1__146985705__146989746
43	1__146986352__146990517
44	1__146987234__146991285
45	1__146988841__146992884

46	1__146989574__146990517
47	1__146989574__146993707
48	1__148118620__148124067
49	1__149057335__149059808
50	1__149077208__149082174
51	1__16579193__16583696
52	1__16580745__16585698
53	1__16581420__16587261
54	1__169704568__169704753
55	1__207532180__207547356
56	1__207542182__207558623
57	1__207545308__207561137
58	1__268667__357586
59	20__1578338__1605059
60	20__1588560__1611713
61	21__8406184__8450243
62	22__29388424__29423188
63	2__95882469__95925424
64	2__97198463__97202393
65	3__169976946__169985865
66	3__169976946__169988359
67	3__58130741__58143613
68	5__179616899__179616958
69	5__179617051__179617110
70	5__181320646__181323959
71	5__21491321__21497196
72	5__70219918__70222015
73	5__70219918__70225795
74	5__71202253__71204350
75	6__117693042__117693167
76	7__10982372__10990847
77	7__142052784__142078470
78	7__142055558__142080945
79	7__142056000__142082210
80	7__142056830__142082571
81	7__142060311__142086328
82	7__142063499__142092047
83	7__142068647__142095713
84	7__142068647__142096415
85	7__142070994__142097649
86	7__26193251__26193373
87	7__38262171__38265678
88	7__38265349__38265678
89	7__39779271__39782799
90	7__73041238__73067927
91	7__73093662__73097567
92	7__77011880__77018997
93	7__77013912__77040089

94	X__131755598__131777744
95	X__15721497__15764775
96	X__15721497__15774397
97	X__3843341__3867806
98	X__3881765__3906232
99	10__68341645__68342031
100	10__71820282__71821965
101	14__39180775__39180921
102	16__20717787__20718493
103	16__368328__368560
104	17__36089986__36195912
105	19__54587201__54633318
106	1__1495485__1529331
107	22__28844127__28846512
108	22__36298919__36300234
109	22__38986298__39025349
110	2__231711914__231712516
111	6__29945451__30009367
112	6__31269966__31354665
113	6__31271073__31355592
114	6__32519370__32580856
115	6__32521905__32581838
116	7__149494204__149621172
117	7__26195853__26196427
118	9__19378368__19378506
119	9__98011280__98012472
120	9__98011289__98012472
121	9__98011316__98012472
122	KI270711.1__20007__25239
123	MT__10783__10923
124	MT__13297__13448
125	MT__13436__13570
126	MT__13846__13989
127	MT__15382__15539
128	MT__8398__8624
129	MT__8468__8624
130	MT__8468__8787
131	MT__8927__9088
132	MT__8927__9189
133	10__100156188__100164049
134	10__102109425__102109457
135	10__125823636__125823722
136	10__13125946__94463319
137	10__17229903__17229938
138	10__17234702__103278090
139	10__17235241__17235315
140	10__27526828__29415412
141	10__45727417__50068008

142	10__45975454__49942300
143	10__45975454__49943358
144	10__78037218__78037304
145	10__93653183__96533190
146	11__111801856__119046793
147	11__1753029__1753126
148	11__319744__319797
149	11__32589012__62389657
150	11__47357067__47357171
151	11__47357079__47357171
152	11__47357096__47357171
153	11__47358187__47358265
154	11__58554841__76691080
155	11__62525113__62525190
156	11__65499766__65499981
157	11__65500559__65500650
158	11__65854885__65855020
159	11__69812153__93790793
160	12__103947311__103947397
161	12__104757639__104757788
162	12__112429463__123323829
163	12__11513300__123615316
164	12__118145459__118145575
165	12__45388238__45388303
166	12__53665247__53665298
167	12__54293252__54293311
168	12__54547080__54547237
169	12__6596006__51377881
170	12__6955732__6956221
171	12__7143163__51979584
172	13__106544140__106557131
173	13__106557048__106557131
174	13__34385990__108303360
175	13__40754019__52175776
176	14__106269016__106557248
177	14__20956274__20956351
178	15__64156056__64156130
179	16__2762939__2763052
180	16__29613103__30309033
181	16__666838__31261797
182	17__20745059__59953111
183	17__44352873__44353002
184	17__45477900__64822393
185	17__58199040__58252506
186	17__64500215__64500328
187	17__7580188__7580273
188	19__11830617__11977164
189	19__12792307__12792416

190	19__18925157__18925188
191	19__34391510__34392259
192	19__4504298__35907257
193	19__48874690__48874773
194	19__54222943__54280299
195	19__54574731__54631787
196	19__54633016__54697945
197	19__6381122__6381401
198	1__109661165__109690564
199	1__150226608__150226694
200	1__151889218__174448125
201	1__155612418__155747896
202	1__156466384__156466520
203	1__16484823__16631109
204	1__1669664__1734835
205	1__171557416__171557460
206	1__20745543__237655742
207	1__20950003__32890794
208	1__235229231__235229290
209	1__23695798__215002685
210	1__244855456__244855963
211	1__244855558__244855963
212	1__25485274__161853323
213	1__25813881__109906644
214	1__30502602__235833667
215	1__31381622__231225516
216	1__33031544__58631445
217	1__35188002__35188058
218	1__4509000__154213946
219	1__70250388__70250447
220	1__77963640__77963690
221	1__82661143__223762251
222	1__99436519__174231355
223	20__43460916__43461038
224	20__43462503__43462577
225	20__58910352__58910401
226	21__33552131__33552193
227	21__43870864__44323049
228	22__20718693__21483090
229	22__22900976__22905025
230	22__36292118__36292234
231	22__36294092__36294154
232	22__37688855__37754984
233	22__42516011__42577098
234	22__50256085__50266611
235	2__107458986__126690311
236	2__119234594__219387163
237	2__140509754__237973225

238	2__145748828__218245541
239	2__159453613__219775189
240	2__197408476__197409798
241	2__231455401__231455463
242	2__231711914__231711977
243	2__231712448__231712516
244	2__231712454__231712516
245	2__25244540__66821244
246	2__26311172__55544363
247	2__47809600__132512957
248	2__54972978__54973133
249	2__55267577__218696526
250	2__68187257__224827411
251	2__68642083__108676028
252	2__74493653__74493785
253	2__97200334__97208006
254	3__10065394__11884098
255	3__121642952__121644898
256	3__128067041__128067076
257	3__143056317__151083239
258	3__185918368__185918427
259	3__38141922__38142025
260	3__38141922__38142061
261	3__48468308__48468394
262	3__50293632__116451746
263	3__50535724__136862311
264	3__75215283__75215363
265	3__9787006__191512541
266	4__73741972__73742032
267	5__133097161__133097278
268	5__134764000__134764092
269	5__32615594__69191840
270	6__15524526__135932054
271	6__18237486__18249695
272	6__29889328__29943543
273	6__29890246__29944616
274	6__30688151__30688219
275	6__31269493__31354526
276	6__31271999__31356957
277	6__31354479__31382078
278	6__32520164__32580856
279	6__32521905__32554971
280	6__32530125__32558186
281	6__32555483__32580856
282	6__32661967__32757883
283	6__33085779__33128098
284	6__33414131__33414273
285	6__7309913__7309981

286	7__142050235__142074173
287	7__143383156__143383322
288	7__20589612__39686781
289	7__37713868__80663167
290	7__38242428__38260230
291	7__5528004__5528039
292	7__5721033__115318270
293	7__75661932__148754150
294	8__18067105__18067133
295	8__19576001__22618495
296	8__89758205__89765415
297	9__128693963__128694001
298	9__128721296__128721358
299	9__129889822__129889913
300	9__129889884__129889913
301	9__19120880__95937869
302	9__36215329__36216369
303	9__93018136__132985466
304	9__98005014__98005064
305	MT__10091__10164
306	MT__10149__10256
307	MT__10843__10903
308	MT__10846__10903
309	MT__10850__10954
310	MT__10948__11040
311	MT__10948__11044
312	MT__10948__11058
313	MT__10966__11058
314	MT__12375__12563
315	MT__13449__13583
316	MT__13846__13998
317	MT__13846__14066
318	MT__13855__13998
319	MT__13978__14124
320	MT__13999__14124
321	MT__14314__14376
322	MT__14329__14412
323	MT__14335__14412
324	MT__14363__14401
325	MT__15368__15490
326	MT__15503__15646
327	MT__1678__1764
328	MT__2822__2985
329	MT__3501__3629
330	MT__3513__3552
331	MT__3598__3677
332	MT__3790__3819
333	MT__4870__4913

334	MT__5309__5368
335	MT__5369__5462
336	MT__6554__6602
337	MT__8398__8467
338	MT__8685__8775
339	MT__8921__8997

Supplementary Table S2. Fusion circRNAs identified by FCIR from the ClinSeq-AML cohort

#FcircRNA_NO	Fusion Name	Backsplice_start	Backsplice_end	Fusion5'_BreakPoint_Pos	Fusion3'_BreakPoint_Pos	Support_FcircRNA_Reads_Count	FcircRNA_Strand_Count(+, -)	patientID
No_1	ETV6--ARNT	12:11675735:+	1:150897438:-	12:11704560:+	1:150868875:-	2	1,1	ALBB13032
No_2	ANKRD28--BCR	3:15738746:-	22:23261848:+	3:15738531:-	22:23194182:+	2	2,0	ALBB13147
No_1	RUNX1--RUNX1T1	21:34859582:-	8:92009009:-	21:34783919:-	8:92017363:-	2	2,0	ALG199931
No_1	ETV6--MN1	12:11793899:+	22:27793130:-	12:11839137:+	22:27796761:-	2	1,1	ALG199944
No_1	ARHGEF12--KMT2A	11:120406114:+	11:118475514:+	11:120407825:+	11:118475519:+	2	1,1	ALG200929
No_1	DGKB--MIPOL1	7:14163134:-	14:37505846:+	7:14255870:-	14:37352431:+	2	2,0	ALG201020
No_1	ANKRD28--BCR	3:15738746:-	22:23261848:+	3:15738567:-	22:23187002:+	2	2,0	ALG200023
No_1	ANKRD28--BCR	3:15738746:-	22:23261847:+	3:15738545:-	22:23240705:+	4	4,0	ALG200319
No_1	KMT2A--MLLT10	11:118484182:+	10:21651768:+	11:118484314:+	10:21651670:+	2	1,1	ALG20032
No_1	INTS4--GAB2	11:77886722:-	11:78230895:-	11:77886458:-	11:78417766:-	2	2,0	ALBB14082
No_1	AFF3--RUNX1	2:100056239:-	21:35412915:-	2:99909164:-	21:35272000:-	2	1,1	ALBB14107
No_1	MAGI3--AKT3	1:113561202:+	1:243660073:-	1:113561199:+	1:243843284:-	2	1,1	ALG20143
No_1	PML--RARA	15:74024126:+	17:40344970:+	15:74024856:+	17:40331396:+	3	2,1	ALBB13106
No_1	RUNX1T1--RUNX1	8:92064056:-	21:34848304:-	8:92017363:-	21:34859473:-	9	5,4	ALBB13153
No_3	RNF216--TIA1	7:5763864:-	2:70224553:-	7:5763864:-	2:70230854:-	2	2,0	ALG199849
No_1	ANKRD28--NUP98	3:15794082:-	11:3805275:-	3:15738746:-	11:3750238:-	2	1,1	ALG199846
No_2	RPSAP52--RAD51B	12:65368908:-	14:68456921:+	12:65758577:-	14:67882996:+	2	2,0	ALG20019
No_1	NUP98--ANKRD28	11:3783314:-	3:15738588:-	11:3715658:-	3:15738746:-	3	1,2	ALG200823

Supplementary Table S3. The list of DE circRNAs between ClinSeq-AML samples and healthy HSPC samples. The last column (hostGene_corr) presents the correlation between expression of circRNA and its host gene.

baseMean	log2FoldChange	lfcSE	stat	pvalue	padj	circbaseID	geneSymbol	circID	hostGene_corr
10.72484524	-1.3967103	0.13693297	-10.199956	1.98E-24	1.07E-20	hsa_circ_0008952	ZDHHC21	9__14639896__14680162	0.344522916
5.217176291	-1.5345926	0.16679567	-9.2004343	3.57E-20	9.59E-17	hsa_circ_0003501	GABPB1	15__50300789__50309798	0.362971783
10.52554114	-1.210783	0.14314485	-8.4584457	2.71E-17	4.86E-14	hsa_circ_0085439	TBC1D31	8__123077111__123105464	0.571837294
14.08583197	-1.5096915	0.18166195	-8.310444	9.53E-17	1.28E-13	hsa_circ_0001524	FNIP1	5__131677703__131709272	0.429728639
4.934605188	-1.595958	0.21108682	-7.560671	4.01E-14	4.31E-11	hsa_circ_0003162	BBS9	7__33146242__33177591	0.42857051
33.80620751	1.75147785	0.24185891	7.24173374	4.43E-13	3.97E-10	hsa_circ_0006156	FNDC3B	3__172247533__172251541	0.52531684
18.39726989	-2.9294278	0.41869862	-6.9965068	2.62E-12	2.02E-09	hsa_circ_0004771	NRIP1	21__15014344__15043574	0.55899772
3.256153864	-1.9119139	0.27620889	-6.9219853	4.45E-12	2.99E-09	hsa_circ_0005406	PHACTR4	1__28459085__28466535	-0.096373621
17.17086625	-1.0165816	0.14792383	-6.8723317	6.32E-12	3.77E-09	hsa_circ_0001726	ZNF394	7__99493040__99494631	0.250495104
3.283859416	-2.9164993	0.43153022	-6.7585053	1.39E-11	7.50E-09	hsa_circ_0001137	DNMT3B	20__32788854__32792770	0.558471718
3.282461247	-1.4194241	0.21247986	-6.6802758	2.38E-11	9.87E-09	NA	METTL6	3__15411245__15415942	0.138536795
2.063625142	-2.8266622	0.42294888	-6.6832241	2.34E-11	9.87E-09	NA	CCDC171	9__15563978__15594172	0.415397374
1.635834704	-2.1411732	0.32276451	-6.6338557	3.27E-11	1.26E-08	NA	IQGAP2	5__76697987__76702590	0.239510803
23.55512959	1.2584049	0.19155841	6.56930111	5.06E-11	1.81E-08	hsa_circ_0000175	ELK4	1__205616478__205623891	0.371261177
7.535466922	4.53687093	0.69312439	6.54553644	5.93E-11	1.99E-08	hsa_circ_0068013	FNDC3B	3__172362633__172381093	0.546752894
2.390141769	-1.7662547	0.27531321	-6.4154377	1.40E-10	4.44E-08	NA	TBC1D31	8__123077111__123144855	0.418838353
47.00755224	3.06908686	0.48359396	6.34641277	2.20E-10	6.26E-08	hsa_circ_0001947	AFF2	X__148661908__148662768	0.829851775
12.97992822	5.32408812	0.84172451	6.32521456	2.53E-10	6.80E-08	hsa_circ_0033144	BCL11B	14__99257471__99257839	0.688018603
4.231597549	-7.9800215	1.27531287	-6.2573049	3.92E-10	1.00E-07	NA	PRKG2	4__81104370__81144330	0.357975538
1.684738628	-2.2711478	0.36641244	-6.1983371	5.71E-10	1.39E-07	NA	TBC1D31	8__123077111__123142456	0.344281882
9.748546473	-1.1300078	0.1834989	-6.1581175	7.36E-10	1.72E-07	NA	RERE	1__8655973__8656441	0.326491176
6.480642421	-1.3561585	0.22086755	-6.1401441	8.24E-10	1.85E-07	hsa_circ_0002158	RERE	1__8541214__8557523	0.341473092
8.668369452	-1.3304758	0.21807795	-6.1009182	1.05E-09	2.27E-07	hsa_circ_0006677	DNAI4	1__66891154__66905375	0.748475269
6.242292035	-1.2026199	0.19772952	-6.0821464	1.19E-09	2.45E-07	hsa_circ_0085440	TBC1D31	8__123077111__123109620	0.526857366
22.57219738	1.23505709	0.20449498	6.03954726	1.55E-09	3.08E-07	hsa_circ_0000799	BPTF	17__67945409__67975958	0.430696874
5.571494952	-2.2196073	0.37343943	-5.9436875	2.79E-09	5.00E-07	NA	PALS2	7__24620052__24668660	0.809745207
4.801756275	-1.2230908	0.20773737	-5.8876784	3.92E-09	6.79E-07	NA	ZNF525	19__53380722__53384056	0.469298322

7.860181494	2.28184489	0.40199549	5.67629472	1.38E-08	2.18E-06	hsa_circ_0005986	PRDM2	1__13731000__13742157	0.248243163
4.093709834	-1.282901	0.2278673	-5.6300355	1.80E-08	2.77E-06	hsa_circ_0008501	RERE	1__8541214__8614686	0.370599893
8.162803829	-3.1463278	0.56208355	-5.5976158	2.17E-08	3.16E-06	hsa_circ_0067735	MED12L	3__151116338__151127984	0.782687512
2.918361602	-2.3677844	0.43395163	-5.4563326	4.86E-08	6.70E-06	hsa_circ_0071174	LRBA	4__150735258__150808398	0.467008485
1.252017374	-1.9311184	0.35588008	-5.4263179	5.75E-08	7.73E-06	hsa_circ_0003586	NF1	17__31223444__31227606	0.22760682
19.97642706	-2.1552084	0.39820883	-5.4122568	6.22E-08	8.16E-06	hsa_circ_0031583	ARHGAP5	14__32090502__32094386	0.88883826
4.436130525	-1.0498598	0.19568602	-5.3650218	8.09E-08	1.04E-05	hsa_circ_0005552	EHBP1	2__62979188__62996766	0.28130558
3.070075741	-2.1532244	0.40260945	-5.3481716	8.88E-08	1.11E-05	hsa_circ_0016601	DNAH14	1__224952670__225007544	0.654964036
18.61131869	-1.1757363	0.22074678	-5.3261762	1.00E-07	1.23E-05	hsa_circ_0000419	RAB3IP	12__69800209__69801721	0.565032132
8.746370843	-1.5363822	0.29119978	-5.2760417	1.32E-07	1.58E-05	hsa_circ_0000099	AMY2B	1__103565435__103575540	0.483218285
5.939957103	2.34687812	0.44808443	5.23758014	1.63E-07	1.87E-05	hsa_circ_0000362	CBL	11__119273868__119274953	0.185718415
1.56260829	-2.0285601	0.38763038	-5.233233	1.67E-07	1.87E-05	hsa_circ_0054214	MAP4K3	2__39336920__39337581	0.260524043
14.54011119	-1.7266149	0.32987564	-5.2341388	1.66E-07	1.87E-05	hsa_circ_0001686	PALS2	7__24623666__24668660	0.89283197
4.411194786	3.58594031	0.68608082	5.22670242	1.73E-07	1.89E-05	hsa_circ_0067991	FNDC3B	3__172251260__172307501	0.473245863
9.90736528	1.71989149	0.33021619	5.20838025	1.90E-07	2.05E-05	hsa_circ_0001756	HIPK2	7__139715932__139717015	0.468291702
3.477831175	-1.2376705	0.23974626	-5.1624186	2.44E-07	2.57E-05	NA	PIBF1	13__72835243__72931267	0.360672162
7.590732166	-1.2016891	0.2329505	-5.1585598	2.49E-07	2.57E-05	hsa_circ_0000233	CCDC7	10__32543300__32584304	0.586862975
5.718835	-1.1312511	0.2204053	-5.1325948	2.86E-07	2.90E-05	hsa_circ_0003054	ATXN10	22__45689712__45718493	0.500239675
2.112277424	-3.3303185	0.64968383	-5.1260603	2.96E-07	2.95E-05	hsa_circ_0003214	LAPTM4B	8__97805353__97825153	0.64954317
0.601116644	-2.8218201	0.55127836	-5.1186846	3.08E-07	2.95E-05	NA	CCDC6	10__59832525__59852702	0.159197442
9.773678006	-1.0356654	0.20220103	-5.1219591	3.02E-07	2.95E-05	hsa_circ_0032649	MLH3	14__75046376__75049718	0.538842452
13.80059675	1.18908005	0.23472347	5.06587616	4.07E-07	3.71E-05	hsa_circ_0005600	YPEL2	17__59353215__59353526	0.243269195
3.32386775	3.00024756	0.59304197	5.0590813	4.21E-07	3.78E-05	hsa_circ_0004550	LYST	1__235793503__235801097	0.522616103
2.075517908	-1.4847011	0.29477695	-5.0366933	4.74E-07	4.18E-05	hsa_circ_0081872	SYPL1	7__106092949__106099282	0.248572207
2.33076508	-4.1599686	0.83177197	-5.001333	5.69E-07	4.93E-05	hsa_circ_0002465	CD109	6__73723251__73736508	0.556302033
11.65670155	-1.0091603	0.20188283	-4.9987426	5.77E-07	4.93E-05	hsa_circ_0001839	KDM4C	9__6880012__6893232	0.313027269
1.975131114	-1.5303807	0.30794738	-4.9696176	6.71E-07	5.55E-05	hsa_circ_0076961	FAM135A	6__70475410__70502791	0.462733284
3.797723384	-1.2980148	0.26430611	-4.9110283	9.06E-07	7.06E-05	hsa_circ_0000439	ATXN2	12__111554158__111555919	0.179323941
3.971799842	-1.3394799	0.27359948	-4.8957693	9.79E-07	7.52E-05	hsa_circ_0001641	HBS1L	6__135039573__135042126	0.229524528
1.035494331	-3.2138282	0.65892842	-4.8773556	1.08E-06	7.91E-05	NA	RAPGEF2	4__159186642__159210583	0.224250123
0.574406108	-3.795943	0.77866284	-4.8749508	1.09E-06	7.91E-05	hsa_circ_0006919	AASS	7__122113118__122133741	0.556346482

3.978904241	3.59463729	0.73918231	4.86299153	1.16E-06	8.29E-05	hsa_circ_0008435	CTDP1	18__79728907__79736521	0.262673493
8.615561786	1.46480418	0.30202716	4.84990883	1.24E-06	8.74E-05	hsa_circ_0001789	RAB11FIP1	8__37877109__37877551	0.184009287
5.943513985	2.09665005	0.43271609	4.84532489	1.26E-06	8.83E-05	NA	BAZ1A	14__34800224__34802988	0.265269592
2.314344439	-2.0118032	0.41649375	-4.8303323	1.36E-06	9.29E-05	hsa_circ_0001687	PALS2	7__24623666__24680520	0.695757423
27.29962188	1.0852502	0.22468758	4.83004095	1.37E-06	9.29E-05	hsa_circ_0001868	AGTPBP1	9__85618983__85633374	0.742933161
3.471004207	-1.058563	0.22074626	-4.7953837	1.62E-06	0.000109149	NA	ZNF638	2__71426460__71427414	0.067674954
13.15111517	1.5196069	0.31755	4.7854098	1.71E-06	0.000113296	hsa_circ_0000690	ITGAL	16__30483827__30484263	0.555152309
4.291312303	-1.2568598	0.26316387	-4.7759585	1.79E-06	0.000115888	NA	DPY19L1P1	7__32632543__32679130	0.555011517
3.965919341	3.61188626	0.76094698	4.74656758	2.07E-06	0.000130905	hsa_circ_0017078	LYST	1__235686949__235697272	0.46920804
5.377981691	-1.5238615	0.32284018	-4.720173	2.36E-06	0.00014736	NA	PALS2	7__24623663__24668660	0.784919274
1.854495205	-1.56442	0.33336093	-4.6928714	2.69E-06	0.000166531	NA	ZFAND4	10__45625951__45627105	0.306462254
4.956186767	-1.1878663	0.25387351	-4.678969	2.88E-06	0.000174508	hsa_circ_0001470	GOLPH3	5__32135572__32143880	0.090762293
0.915741694	-2.6475762	0.56588646	-4.678635	2.89E-06	0.000174508	hsa_circ_0079449	TMEM106B	7__12224226__12229819	0.128129608
2.867224441	-1.3480175	0.28836554	-4.6746829	2.94E-06	0.000175925	hsa_circ_0085441	TBC1D31	8__123077111__123120188	0.309660349
1.311490345	-4.8520217	1.03976191	-4.6664738	3.06E-06	0.000181087	NA	PLOD2	3__146121112__146124229	0.534337811
5.971207506	-1.4267386	0.30722861	-4.6438988	3.42E-06	0.00019986	hsa_circ_0001685	PALS2	7__24623666__24650712	0.804016156
1.466424633	-2.2136156	0.47757047	-4.63516	3.57E-06	0.00020625	NA	MAP7	6__136388393__136389517	0.60070582
12.92226815	-1.5695046	0.33897975	-4.6300837	3.66E-06	0.000209123	hsa_circ_0000660	MCTP2	15__94356137__94402019	0.746605472
3.401585368	2.82671585	0.6138535	4.60487049	4.13E-06	0.000228827	hsa_circ_0084582	CHD7	8__60741259__60743097	0.644446848
14.26122156	1.68200903	0.36641435	4.59045618	4.42E-06	0.00024026	hsa_circ_0008934	ASAP1	8__130152736__130180880	0.737279811
5.469065704	2.07248292	0.45571056	4.54780535	5.42E-06	0.000279819	hsa_circ_0004861	PRDM2	1__13731000__13749487	0.237841042
0.809262313	-2.3643053	0.52006567	-4.5461668	5.46E-06	0.000279819	hsa_circ_0071198	LRBA	4__150806271__150831976	0.233252946
9.142403389	2.70014002	0.59368571	4.54809672	5.41E-06	0.000279819	hsa_circ_0091669	AFF2	X__148651999__148662768	0.754274097
1.079993282	-1.6386334	0.36142988	-4.5337518	5.79E-06	0.00029399	hsa_circ_0088369	RC3H2	9__122853952__122855878	0.217908236
1.058704197	-1.7577925	0.3887915	-4.5211702	6.15E-06	0.000306241	NA	RABGAP1	9__122957011__123020459	0.20565256
14.98734643	-1.0402374	0.23022491	-4.5183528	6.23E-06	0.000307496	NA	ATP8B4	15__49916934__49934182	0.579569992
2.422115603	2.53260063	0.56480753	4.48400645	7.33E-06	0.000358148	hsa_circ_0005589	ARCN1	11__118583809__118584644	0.008744959
8.856311329	-1.6774072	0.37645612	-4.4557841	8.36E-06	0.000401367	hsa_circ_0002454	DNAJC6	1__65364635__65366196	0.731775408
0.994268047	-2.6330745	0.59080974	-4.4567215	8.32E-06	0.000401367	hsa_circ_0007158	FAM169A	5__74813840__74841679	0.633455866
1.458909591	-2.517606	0.56530119	-4.4535658	8.45E-06	0.00040195	hsa_circ_0001742	SMO	7__129205203__129206587	0.633952204
1.841067324	-1.4021371	0.31683176	-4.4254941	9.62E-06	0.00045393	hsa_circ_0003587	KATNAL1	13__30227412__30241086	0.33653967

3.02557308	3.0056912	0.68085267	4.41459852	1.01E-05	0.000473253	hsa_circ_0000692	ITGAM	16__31297514__31297954	0.672022284
1.689962867	-1.8054806	0.40915829	-4.4126701	1.02E-05	0.000473373	hsa_circ_0005348	ZC3H12C	11__110136663__110137414	0.496017259
2.433124296	-1.1441749	0.25970244	-4.405715	1.05E-05	0.000484643	hsa_circ_0005684	DENND4C	9__19286769__19305527	0.295982326
0.83199126	-2.8456922	0.64692344	-4.3988084	1.09E-05	0.000496083	hsa_circ_0071224	LRBA	4__150850724__150868305	0.28310272
4.070924138	-1.0242543	0.23366111	-4.3835037	1.17E-05	0.000523394	hsa_circ_0007822	KDM1A	1__23030469__23059167	0.343003962
2.078418959	-1.3980207	0.3202583	-4.3652912	1.27E-05	0.000559636	hsa_circ_0065249	SMARCC1	3__47622207__47678311	0.343394194
5.715371637	2.5159495	0.57733719	4.35785109	1.31E-05	0.00057429	hsa_circ_0001361	FNDC3B	3__172112452__172133546	0.523220765
8.15165289	1.20903577	0.27800293	4.34900376	1.37E-05	0.00059313	hsa_circ_0001861	GRHPR	9__37424845__37426654	0.242704194
1.729243104	-1.6404802	0.37783863	-4.3417482	1.41E-05	0.000608159	NA	IQGAP2	5__76697987__76701213	0.295883534
2.754872264	-3.2724983	0.75458658	-4.3368096	1.45E-05	0.000617043	hsa_circ_0000551	SLC8A3	14__70166639__70168484	0.75037358
0.602118929	-3.2126892	0.74172912	-4.3313511	1.48E-05	0.000627562	hsa_circ_0001103	SERPINE2	2__223998115__224001922	0.444627977
3.572836558	2.54176155	0.5906598	4.30325807	1.68E-05	0.000707142	hsa_circ_0000190	CNIH4	1__224365879__224371423	0.371594591
3.173007894	-1.1435278	0.26670351	-4.2876366	1.81E-05	0.000747063	hsa_circ_0007693	ERI3	1__44308310__44339322	0.242081391
5.41709229	2.10693498	0.49137883	4.28780173	1.80E-05	0.000747063	hsa_circ_0007609	DNAJC5	20__63928335__63931022	0.421310724
1.693072357	-1.8142489	0.42476772	-4.2711554	1.94E-05	0.000792289	hsa_circ_0005171	TMEM38B	9__105705597__105722621	0.3898386
9.721654788	1.25050282	0.29348666	4.2608507	2.04E-05	0.000823482	hsa_circ_0000754	SSH2	17__29684563__29703062	0.491301885
3.877451871	2.28772304	0.53925308	4.2423922	2.21E-05	0.000868134	NA	CBL	11__119284969__119285566	0.292455215
3.199284322	-2.0459773	0.48204414	-4.2443775	2.19E-05	0.000868134	hsa_circ_0054598	RTN4	2__55025086__55028220	0.275337137
1.522320107	-2.0853056	0.49139396	-4.2436533	2.20E-05	0.000868134	hsa_circ_0004119	RAB23	6__57193842__57210445	0.535793639
3.152846578	2.5598451	0.60364609	4.2406389	2.23E-05	0.000868603	hsa_circ_0008927	ATG7	3__11358418__11380052	0.329567272
2.513096977	-1.816916	0.42957168	-4.2295991	2.34E-05	0.000887749	hsa_circ_0006751	HACD1	10__17603560__17604047	0.518905186
4.986413292	3.58843896	0.84816714	4.23081581	2.33E-05	0.000887749	hsa_circ_0000277	PDE3B	11__14771937__14789242	0.292139376
0.942540167	-2.3570004	0.55730027	-4.2293187	2.34E-05	0.000887749	NA	C17orf67	17__56814869__56825321	0.240531449
6.075011221	-1.4737862	0.3492284	-4.2201214	2.44E-05	0.00091191	NA	GUCY1A1	4__155696756__155722192	0.698932939
10.1678839	-1.6122216	0.38469845	-4.190871	2.78E-05	0.001019665	hsa_circ_0031584	ARHGAP5	14__32090502__32117287	0.867785469
3.274092331	-1.1475033	0.27615108	-4.1553463	3.25E-05	0.001164499	NA	SENP6	6__75659262__75678927	0.208255309
3.204315786	-2.4464978	0.59061482	-4.1422898	3.44E-05	0.001205874	hsa_circ_0004405	FAM169A	5__74834426__74841679	0.717462315
5.674655834	2.0710816	0.50010111	4.14132577	3.45E-05	0.001205874	hsa_circ_0008205	TMEM71	8__132722040__132757294	0.43190281
0.617187022	-2.660855	0.64355689	-4.1346072	3.56E-05	0.001233686	NA	TTLL7	1__83911165__83947282	0.377531608
0.608689123	-2.6623959	0.64700067	-4.1149817	3.87E-05	0.00132638	hsa_circ_0007661	RABGAP1	9__123070350__123076762	0.235440509
35.26741821	1.17711537	0.2865842	4.10739803	4.00E-05	0.001362	NA	LINC00869	1__149655699__149669310	0.787490354

1.264909154	-1.6252253	0.39731732	-4.0904969	4.30E-05	0.00144685	hsa_circ_0008790	CLNS1A	11__77622500__77625818	0.166932881
2.393129817	-2.1912829	0.53739102	-4.0776321	4.55E-05	0.001519758	hsa_circ_0000199	AKT3	1__243545510__243573048	0.636158193
2.929063316	3.17574344	0.78906078	4.02471333	5.70E-05	0.001870651	hsa_circ_0067973	FNDC3B	3__172112452__172251541	0.413419062
6.919481367	1.45229648	0.36183362	4.01371352	5.98E-05	0.001924837	hsa_circ_0005899	LYST	1__235830226__235833667	0.496774577
6.873379114	-1.767514	0.44059454	-4.0116567	6.03E-05	0.00193013	hsa_circ_0052318	ZNF418	19__57925623__57928047	0.874220026
1.924629396	-1.3289714	0.33195418	-4.0034783	6.24E-05	0.001986293	hsa_circ_0006118	UMPS	3__124737568__124744203	0.264567308
1.601658212	-1.7390745	0.43600567	-3.9886511	6.65E-05	0.002089874	hsa_circ_0001709	NIPSNAP2	7__55982210__55983868	0.149535758
1.530244071	-1.2874545	0.32320922	-3.983347	6.80E-05	0.002124667	hsa_circ_0073486	CHD1	5__98868495__98872555	0.001898168
10.10598988	1.62147258	0.40737952	3.98025057	6.88E-05	0.002140092	NA	FLT3	13__28024861__28028288	0.598620073
1.02892529	-4.5330572	1.14087419	-3.9733191	7.09E-05	0.002178182	hsa_circ_0004365	SEMA3C	7__80789306__80810701	0.502147208
5.211744201	-2.8334453	0.71623248	-3.9560414	7.62E-05	0.002302327	NA	AKT3	1__243545510__243695716	0.717970601
1.469294891	-1.4431011	0.36526524	-3.9508307	7.79E-05	0.002339893	NA	XPO1	2__61482957__61493053	0.159342231
0.790562014	-1.7873771	0.45324528	-3.9435096	8.03E-05	0.002399119	hsa_circ_0039363	CHD9	16__53254438__53268126	0.120401849
2.879298604	-1.0013989	0.25410015	-3.9409615	8.12E-05	0.002411354	hsa_circ_0007456	MAP2K4	17__12081356__12113360	0.235954982
2.170103079	-2.1295163	0.54125752	-3.9343866	8.34E-05	0.002464703	hsa_circ_0071176	LRBA	4__150735258__150831976	0.373007657
8.879422972	1.98861103	0.5058323	3.93136424	8.45E-05	0.002482261	NA	CSF2RA	X__1285778__1290509	0.67784954
3.996515595	1.74418373	0.44550291	3.91508945	9.04E-05	0.002627101	NA	MIS18BP1	14__45246743__45247377	0.270276311
1.249185101	-1.361194	0.3488446	-3.902007	9.54E-05	0.00268614	hsa_circ_0044413	ZNF652	17__49311312__49317983	0.200887253
4.042914477	-1.0223035	0.26193882	-3.902833	9.51E-05	0.00268614	hsa_circ_0002211	DDX17	22__38494630__38501280	-0.115581074
1.840027317	-2.0790521	0.5323117	-3.9057043	9.40E-05	0.00268614	NA	TIPARP	3__156677657__156678614	0.302637832
1.699088421	2.19665154	0.56257231	3.90465638	9.44E-05	0.00268614	hsa_circ_0085459	ATAD2	8__123333878__123337821	0.257500037
2.609323188	2.63115006	0.67512847	3.89725833	9.73E-05	0.002725073	NA	OXNAD1	3__16301626__16302748	0.282514117
2.509685618	-1.2781221	0.32823299	-3.8939478	9.86E-05	0.002748238	hsa_circ_0000500	PCCA	13__100257595__100273346	0.441943863
0.801660015	-1.7008036	0.43771639	-3.8856293	0.000102065	0.002808328	NA	VPS54	2__63949037__63965966	0.104290303
1.31951851	-1.7534356	0.45112605	-3.8867974	0.000101575	0.002808328	NA	MAP7D3	X__136241160__136244795	0.115372879
2.305813758	-4.0251816	1.03609482	-3.8849549	0.000102349	0.002808328	NA	CHMP1B2P	X__80288906__80310233	0.669548827
3.96257458	2.0401406	0.52615547	3.87744824	0.000105558	0.002881674	hsa_circ_0004276	SUCLG2	3__67495798__67508903	0.455726995
2.432150508	-1.3284371	0.34293628	-3.873714	0.000107189	0.002911432	hsa_circ_0005935	NARS2	11__78465876__78493195	0.406002599
10.41102344	5.0020479	1.29248655	3.87009667	0.000108792	0.002940123	hsa_circ_0001760	MGAM	7__142059856__142085961	0.632174054
2.161662084	2.05799143	0.53371745	3.85595679	0.000115278	0.003099821	NA	KMT2C	7__152238707__152250966	0.11148769
8.185469485	1.11702322	0.2899955	3.85186399	0.000117222	0.003120894	hsa_circ_0000442	MED13L	12__116230433__116237705	0.128825651

0.921942596	-1.9910082	0.51852478	-3.8397552	0.000123157	0.003262752	hsa_circ_0001177	ENSG00000180530:<br>ENSG00000231201:<br>ENSG00000235609	21__14968297__15043574	0
1.058868807	-2.2971926	0.59862891	-3.8374233	0.000124332	0.003277734	hsa_circ_0000603	TEX9	15__56388472__56394834	0.422813321
3.091730994	-1.5829645	0.41302386	-3.8326223	0.000126785	0.003293947	NA	ITPR2	12__26481131__26655852	0.518210652
2.182410252	2.77722232	0.72531064	3.82901084	0.000128659	0.003317492	NA	MTMR10	15__30974314__30976955	0.074093494
2.816343177	2.36289302	0.6173739	3.82732895	0.000129541	0.003317492	NA	ITGAL	16__30496097__30496566	0.347840817
2.064314462	-1.6462073	0.43002551	-3.828162	0.000129104	0.003317492	hsa_circ_0087243	VPS13A	9__77214329__77227485	0.305695829
2.416312114	-1.6096737	0.42089471	-3.8244095	0.000131086	0.003341136	hsa_circ_0079422	ICA1	7__8218305__8236005	0.572731089
2.346978107	-1.1468204	0.3005073	-3.8162813	0.000135478	0.003436797	hsa_circ_0003664	LARP4	12__50427762__50430570	0.216162508
1.062215749	-1.503322	0.39596898	-3.7965652	0.000146715	0.003691442	NA	UPF2	10__12028745__12029524	0.193905818
4.224974654	1.72957536	0.455644	3.7958919	0.000147114	0.003691442	NA	NCOA4	10__46010223__46011206	0.443045008
3.205902324	2.95318368	0.77815403	3.79511454	0.000147575	0.003691442	hsa_circ_0015259	SUCO	1__172551512__172570730	0.229514569
6.855034289	1.91374886	0.50470557	3.79181246	0.000149552	0.003706404	hsa_circ_0005838	AXIN1	16__346148__347106	0.2818014
2.115135597	2.32149213	0.612223	3.79190612	0.000149495	0.003706404	hsa_circ_0002094	RBM33	7__155706860__155718443	0.160850494
7.799820043	2.57562716	0.68131597	3.78037106	0.000156595	0.003863151	hsa_circ_0087960	LPAR1	9__110972073__110973558	0.77022761
6.300333479	1.46136141	0.38729837	3.7732186	0.000161155	0.003957495	hsa_circ_0002926	KDM4B	19__5047476__5082504	0.270393105
4.349298344	-1.1930481	0.31690947	-3.7646337	0.000166793	0.004077341	hsa_circ_0008647	PRELID2	5__145796442__145826200	0.598308861
1.293941908	-1.4847988	0.39467224	-3.762106	0.000168489	0.004100143	hsa_circ_0002255	MESD	15__80978766__80982182	0.017649932
3.311398441	-1.0271863	0.27353497	-3.7552286	0.000173183	0.004176592	hsa_circ_0009038	WDR7	18__56758865__56781656	0.12101208
7.533096428	1.22649194	0.32652791	3.75616262	0.000172539	0.004176592	hsa_circ_0001112	DGKD	2__233388257__233390483	0.346946355
0.99747458	-3.3925374	0.90565957	-3.7459301	0.000179727	0.004295866	NA	STK33	11__8413495__8464822	0.328231163
0.814851896	-1.7699736	0.47282931	-3.7433669	0.000181571	0.004320742	NA	FAM228B	2__24137909__24147086	0.039269794
7.70547021	3.1919969	0.8553857	3.73164632	0.000190232	0.00448715	hsa_circ_0007120	SKI	1__2302978__2304585	0.483418423
3.524529053	2.13460936	0.57310695	3.72462655	0.000195605	0.004593718	NA	LYST	1__235780865__235782087	0.556329444
4.942313915	1.42858919	0.38454661	3.71499614	0.000203207	0.004730936	hsa_circ_0005227	TANK	2__161179614__161204793	0.22035241
4.287809712	1.85719119	0.50211121	3.69876466	0.000216651	0.004979278	hsa_circ_0002925	KIAA1958	9__112574057__112575251	0.548716564
1.910894928	2.59951839	0.7031377	3.69702607	0.00021814	0.004992156	hsa_circ_0000278	PDE3B	11__14859043__14861366	0.423404227
20.15927003	1.53262205	0.41528106	3.69056574	0.000223756	0.005077464	hsa_circ_0004658	EMILIN2	18__2890561__2892486	0.706165594
1.967893457	2.41622599	0.65614126	3.68247839	0.000230977	0.005197476	NA	LYST	1__235780865__235801097	0.45032393
3.479491039	2.0306967	0.55167763	3.68094806	0.000232368	0.005206987	hsa_circ_0010028	PRDM2	1__13715541__13742157	0.23189094
5.782647976	1.20441273	0.32813624	3.67046548	0.000242109	0.005358284	hsa_circ_0008870	MAPK1	22__21805850__21807846	0.220481021

2.176955682	-1.5399418	0.42122438	-3.6558705	0.000256311	0.005649339	hsa_circ_0004662	ENSG00000112096	6__159682474__159688242	0
1.159819507	-2.3000394	0.63046374	-3.6481708	0.000264114	0.005797572	hsa_circ_0071185	LRBA	4__150761783__150808398	0.389941427
3.177607607	-3.5965919	0.98623286	-3.6467978	0.000265529	0.005804931	NA	TPTEP1	22__16637040__16638740	0.593953385
1.150653985	-1.4337006	0.3944091	-3.6350595	0.000277916	0.006051153	hsa_circ_0005745	EFL1	15__82151465__82152423	0.199915269
1.585280298	-1.4704004	0.40570193	-3.6243368	0.000289704	0.006270919	NA	ZFAND4	10__45639816__45663842	0.277134908
1.58145008	-1.6070594	0.44357597	-3.6229633	0.000291247	0.006270919	hsa_circ_0008802	SLC11A2	12__51008476__51010766	0.284794737
5.481902322	1.42745628	0.39402758	3.62273186	0.000291508	0.006270919	hsa_circ_0000652	IQGAP1	15__90441506__90443478	0.493619756
1.604197926	-1.1993654	0.33329734	-3.5984847	0.000320077	0.006830843	NA	AHCTF1	1__246913232__246918377	-0.045515597
1.367884274	-1.4360018	0.40007517	-3.5893299	0.000331529	0.007019541	hsa_circ_0012144	ERI3	1__44308310__44319744	0.243822405
4.855932735	-1.315734	0.36669223	-3.5881155	0.000333077	0.007024655	hsa_circ_0000230	ZEB1	10__31373018__31461237	0.667641852
1.110633485	-1.7930101	0.49995228	-3.5863625	0.000335323	0.007044397	hsa_circ_0006908	CLIP4	2__29133655__29145367	0.384685047
3.660460624	-1.9073379	0.53282551	-3.579667	0.000344032	0.007199245	hsa_circ_0003823	CEP70	3__138570318__138572984	0.508595338
1.158938509	-1.8578633	0.51969658	-3.5749	0.000350362	0.00727508	NA	GINM1	6__149572285__149582603	0.122331459
1.23195886	-1.3788537	0.38674574	-3.5652718	0.000363479	0.007518429	hsa_circ_0002798	STK3	8__98526742__98596169	0.16824997
4.964505842	2.69004058	0.75644734	3.55615051	0.000376329	0.007709125	hsa_circ_0017648	SFMBT2	10__7367649__7381949	0.702237224
1.183065605	-2.0000743	0.56252279	-3.5555436	0.000377198	0.007709125	hsa_circ_0007843	ARHGAP32	11__129123446__129164427	0.569818977
4.37095248	1.73314939	0.48770407	3.55369063	0.000379866	0.007709125	NA	RAB3D	19__11335447__11335783	0.3467806
5.150091955	1.30968106	0.36847037	3.55437283	0.000378882	0.007709125	hsa_circ_0008982	DIAPH1	5__141582312__141583615	0.282850887
1.142191279	-1.8892092	0.53153914	-3.5542241	0.000379096	0.007709125	hsa_circ_0004582	PALS2	7__24620052__24650712	0.569072467
2.463252976	1.88691493	0.53131641	3.55139595	0.000383193	0.007747423	hsa_circ_0007706	USP14	18__196637__199316	0.077448049
0.78134266	-2.349188	0.66211899	-3.5479847	0.000388191	0.00776093	hsa_circ_0071189	LRBA	4__150798081__150831976	0.372698217
4.97853366	-1.0380494	0.29308983	-3.5417449	0.00039749	0.007917405	hsa_circ_0004689	SWT1	1__185214507__185231708	0.547327892
1.234186	-1.3915162	0.39400262	-3.5317434	0.00041283	0.008181643	hsa_circ_0006662	GLS	2__190900564__190905167	0.250140518
2.702370735	-1.0501004	0.2973842	-3.5311237	0.000413798	0.008181643	NA	AFG2A	4__122927634__122979386	0.232718242
0.915867157	-1.9304672	0.54723463	-3.5276774	0.000419223	0.008258535	hsa_circ_0003541	ZC3H12C	11__110136663__110159490	0.380343942
3.559723289	1.58281737	0.44934811	3.52247473	0.000427538	0.008361085	NA	NHLRC2	10__113876521__113884380	0.010690559
4.727038188	-1.3181623	0.37543827	-3.510996	0.000446431	0.008698935	hsa_circ_0016600	DNAH14	1__224952670__224974153	0.738146694
1.038724614	-1.4377089	0.41101654	-3.4979344	0.000468876	0.009070567	hsa_circ_0025951	ARID2	12__45836589__45839496	0.055275252
3.404964596	-1.2967081	0.37136217	-3.4917615	0.000479847	0.009249516	hsa_circ_0003218	BMPR2	2__202464809__202467689	0.641602759
3.467048451	1.96029601	0.56403849	3.47546497	0.000509969	0.009760182	hsa_circ_0072340	FYB1	5__39201826__39202987	0.378020924
0.772534149	-1.7399506	0.50210726	-3.4652967	0.000529647	0.010057948	hsa_circ_0006297	TTBK2	15__42827928__42878684	0.138416844

1.120490626	-2.0622688	0.59527423	-3.4644013	0.000531413	0.010057948	hsa_circ_0003165	FANCL	2__58198579__58232112	0.206545571
0.742777931	-2.15272	0.62151047	-3.4636907	0.000532819	0.010057948	hsa_circ_0004873	OXCT1	5__41794003__41807438	0.217933426
0.835566358	-2.7724578	0.80119026	-3.4604238	0.000539326	0.010106253	NA	SETBP1	18__44949881__44953340	0.547137958
2.678844356	2.11358122	0.61069176	3.46096242	0.000538248	0.010106253	hsa_circ_0006787	PARP8	5__50759643__50763242	0.403514099
4.083231175	-1.246315	0.3603234	-3.4588788	0.000542429	0.010129108	NA	MCTP2	15__94356137__94385525	0.644914553
3.470866772	2.00427159	0.57987711	3.45637299	0.000547497	0.010188373	hsa_circ_0004901	APAF1	12__98677425__98686873	0.397964211
0.978616033	-2.6113949	0.75699815	-3.4496715	0.000561269	0.010367575	NA	L3MBTL4	18__6213149__6312056	0.656956703
1.502420848	1.98010807	0.57529059	3.44192672	0.000577587	0.010565514	hsa_circ_0037158	AXIN1	16__304304__314683	0.215872882
0.980097568	-1.7365347	0.50535867	-3.436242	0.000589844	0.010716823	hsa_circ_0002353	ATXN1	6__16585780__16657901	0.289857929
2.399001544	-1.1566751	0.3381852	-3.4202417	0.000625655	0.011215912	hsa_circ_0031940	TXNDC16	14__52536719__52544371	0.340899489
0.694006349	-1.7903693	0.52334045	-3.4210413	0.000623819	0.011215912	NA	MCTP2	15__94278154__94298730	0.397715628
1.640024858	2.16354136	0.63246446	3.42081094	0.000624347	0.011215912	NA	CHD7	8__60794986__60801593	0.509354844
1.987729784	1.89782959	0.55624588	3.41185374	0.000645227	0.011414579	hsa_circ_0004682	CDR2	16__22349279__22349849	0.148920086
2.008902289	1.92973117	0.56589729	3.41003783	0.000649539	0.011414916	hsa_circ_0027910	GNPTAB	12__101780152__101790057	0.327393623
4.894142123	-1.1052137	0.32418872	-3.4091678	0.000651614	0.011414916	hsa_circ_0009043	EXOC6B	2__72718103__72733118	0.579270007
1.767109791	-1.0607315	0.31100541	-3.4106528	0.000648075	0.011414916	hsa_circ_0088389	RC3H2	9__122877471__122883379	0.237899375
0.965515427	-1.5407715	0.45262858	-3.4040527	0.00066394	0.011481244	NA	FOXJ3	1__42265115__42323827	0.229160962
0.612945512	-1.9176828	0.56297221	-3.4063544	0.000658367	0.011481244	hsa_circ_0002039	OSBPL9	1__51772070__51772723	0.168438882
1.078510609	-1.3995746	0.41108306	-3.404603	0.000662603	0.011481244	hsa_circ_0056589	UBXN4	2__135748267__135770735	0.064393024
0.910246408	-1.4496147	0.42566106	-3.4055611	0.000660282	0.011481244	hsa_circ_0008755	CPLANE1	5__37243013__37247745	0.199700265
1.391800562	-2.2909551	0.67410366	-3.3985204	0.000677514	0.011678431	hsa_circ_0002669	DOCK1	10__126970702__127000307	0.645249945
2.162714791	1.86335772	0.54950022	3.39100451	0.00069637	0.011814118	hsa_circ_0023928	PICALM	11__86000643__86031611	0.289930563
1.832152824	-1.1772314	0.3469468	-3.3931178	0.000691019	0.011814118	hsa_circ_0032116	MNAT1	14__60796217__60818847	0.269893914
0.907282896	-1.8186467	0.53628497	-3.3911946	0.000695887	0.011814118	hsa_circ_0002528	NEK7	1__198232553__198264235	-0.016668666
4.646519489	-1.0273248	0.30293203	-3.3912715	0.000695691	0.011814118	hsa_circ_0003400	CRBN	3__3156219__3174261	0.282441626
6.01402786	-1.1211892	0.33037228	-3.3937147	0.000689515	0.011814118	NA	SLC39A8	4__102304317__102324998	0.454443793
1.267039258	-1.5314814	0.45189733	-3.3890029	0.000701473	0.011863269	hsa_circ_0001134	XRN2	20__21339044__21344208	0.11508934
1.330380039	-1.388538	0.41038047	-3.3835382	0.000715582	0.012063958	hsa_circ_0086421	CNTLN	9__17309058__17416189	0.271006852
0.599975202	-1.7773363	0.52544723	-3.3825211	0.000718238	0.012070881	hsa_circ_0002603	UBR3	2__169872236__169878574	0.100800121
1.710283782	2.1978738	0.65095908	3.37636245	0.000734511	0.0122677	hsa_circ_0006948	FNDC3B	3__172112452__172226947	0.417928205
1.362328147	-1.3236167	0.39233009	-3.3737323	0.000741564	0.012327385	hsa_circ_0045455	BPTF	17__67910877__67913187	0.198644632

0.778805808	-2.3210461	0.68805932	-3.3733226	0.000742669	0.012327385	NA	ZNF521	18__25322008__25327448	0.58565088
1.350083506	-1.507767	0.44729448	-3.3708598	0.00074934	0.012399846	hsa_circ_0018403	SGMS1	10__50460673__50590247	0.134092106
1.934286401	2.13322444	0.63360299	3.36681563	0.000760415	0.012544516	hsa_circ_0010029	PRDM2	1__13778418__13782831	0.22607167
4.122348381	2.04621336	0.60882495	3.36092232	0.000776827	0.012776067	hsa_circ_0001152	SAMHD1	20__36911218__36912552	0.61003436
3.345986509	1.48104901	0.44102057	3.35823113	0.00078443	0.012861781	hsa_circ_0006396	BRAP	12__111679151__111683307	0.089628234
4.405037131	2.84701647	0.8487579	3.35433281	0.000795566	0.013004726	hsa_circ_0000665	UBE2I	16__1314020__1320517	0.131240358
3.321312468	1.61215933	0.48110337	3.35096244	0.000805312	0.013084499	hsa_circ_0004162	PATL1	11__59658866__59659469	0.109110371
2.125938574	-2.2456831	0.67094237	-3.3470581	0.000816741	0.013230217	hsa_circ_0017251	AKT3	1__243613671__243695716	0.683395021
2.742054341	-1.0751343	0.3213092	-3.3461048	0.000819554	0.013235923	hsa_circ_0055019	APLF	2__68490190__68526242	0.342154853
1.728779704	-1.0794081	0.32345874	-3.3370811	0.000846632	0.013591608	hsa_circ_0000564	DICER1	14__95140659__95141748	0.189186779
1.16320424	-2.0922002	0.62727	-3.3354061	0.000851749	0.013633057	hsa_circ_0003390	INTU	4__127669036__127687867	0.336088983
2.681464963	-1.0473548	0.31489319	-3.3260637	0.000880818	0.01399078	hsa_circ_0078617	MAP3K4	6__161034259__161049979	0.201038132
1.379735632	-1.3810738	0.41774795	-3.3059978	0.000946388	0.014838707	hsa_circ_0024037	MRE11	11__94445810__94447438	0.203913519
5.035284669	1.21925789	0.36955706	3.29924122	0.000969466	0.015156356	hsa_circ_0006681	TBC1D14	4__6994184__7001251	0.242902675
3.040729268	1.50433102	0.45688538	3.29257859	0.000992731	0.015475101	hsa_circ_0005199	UBE4B	1__10095461__10105744	0.223475931
2.126416279	1.81623449	0.55431486	3.27653942	0.001050876	0.016240268	hsa_circ_0084620	YTHDF3	8__63186147__63187745	0.227181314
13.86518594	1.822939	0.55689571	3.27339388	0.001062643	0.016328266	hsa_circ_0006209	CTDP1	18__79695225__79704917	0.529418341
16.46606023	1.00249337	0.30622053	3.27376277	0.001061257	0.016328266	hsa_circ_0001459	NEIL3	4__177353308__177353728	0.63695296
1.673651922	2.16395474	0.66128683	3.2723391	0.001066616	0.016342618	hsa_circ_0024048	PIWIL4	11__94583448__94608686	0.483549931
2.428942022	-1.3233679	0.40479803	-3.2692054	0.0010785	0.01643108	NA	ZNF66	19__20792512__20793878	0.162601095
6.583462338	1.35397293	0.41407962	3.26983714	0.001076094	0.01643108	hsa_circ_0003553	RCAN3	1__24514314__24514567	0.495108924
0.52702941	-1.8781409	0.57508948	-3.2658239	0.001091461	0.016534867	NA	ARL13B	3__94003659__94003908	0.102832633
1.429557359	-1.8562228	0.56927968	-3.2606518	0.001111565	0.016745083	hsa_circ_0008202	SPATA6	1__48355670__48359770	0.497502916
0.580788957	-1.7718498	0.54330992	-3.2612138	0.001109364	0.016745083	NA	RAPGEF2	4__159304342__159314768	0.168556033
2.584338254	-1.175877	0.36086622	-3.2584846	0.00112009	0.016826373	hsa_circ_0002457	ATXN2	12__111552280__111555919	0.149409177
1.252961872	-1.3367985	0.41069187	-3.2549915	0.001133958	0.016987256	hsa_circ_0007225	RPE	2__210015973__210021064	0.0342399
1.959729977	2.60010449	0.79918756	3.25343462	0.00114019	0.017033166	hsa_circ_0004296	ETS1	11__128484823__128490576	0.504875856
2.154628094	-1.5711496	0.48335263	-3.2505246	0.001151923	0.01710197	NA	PLXNC1	12__94220016__94227235	0.491916399
0.690325356	-1.6662081	0.51269049	-3.2499297	0.001154335	0.01710197	NA	RC3H2	9__122865349__122883379	0.087060062
0.705680575	-1.7729708	0.54623775	-3.245786	0.001171268	0.017257757	NA	GOLM2	15__44331988__44380976	0.03731499
2.116906774	-1.0093366	0.31121487	-3.2432147	0.001181891	0.017319375	hsa_circ_0002466	TTBK2	15__42810614__42840433	0.214085994

1.300059531	-1.4351246	0.44247526	-3.2434008	0.001181119	0.017319375	hsa_circ_0007936	CD2AP	6__47503280__47504758	0.27958974
2.543506206	-1.0503241	0.32403295	-3.2414116	0.001189393	0.017381949	hsa_circ_0073517	FBXL17	5__108186117__108348530	0.200548303
6.585660028	2.73915472	0.845276	3.24054478	0.001193015	0.017387633	hsa_circ_0003692	FNDC3B	3__172251260__172310881	0.429268888
4.349383496	1.4254566	0.44061564	3.23514754	0.001215799	0.017576791	hsa_circ_0001146	CPNE1	20__35653528__35659014	0.092623046
2.138887919	2.1626242	0.67159479	3.22013248	0.001281314	0.018379363	NA	DMXL2	15__51535663__51547408	0.581669009
3.904879202	1.50898532	0.46861791	3.22007607	0.001281566	0.018379363	hsa_circ_0000105	CD53	1__110891392__110892533	0.272748735
6.551681788	-1.7077769	0.53031835	-3.2202863	0.001280626	0.018379363	hsa_circ_0054597	RTN4	2__55025086__55027485	0.398950154
1.717980996	2.03713129	0.63411306	3.21256795	0.00131554	0.018766513	hsa_circ_0003060	SUCLG2	3__67495798__67498295	0.219022909
5.151426107	1.41730689	0.44171425	3.20865103	0.001333592	0.018973704	hsa_circ_0002360	RUNX1	21__34834410__34859578	0.50783643
3.641779029	3.13051333	0.97736856	3.20300186	0.001360031	0.019248018	hsa_circ_0017641	SFMBT2	10__7276892__7381949	0.621975997
2.283235327	2.80791692	0.87691761	3.20203048	0.001364626	0.019262353	hsa_circ_0006648	CSF2RA	X__1282678__1290509	0.453886823
2.427521209	-1.0396563	0.32522164	-3.1967622	0.001389794	0.019566267	hsa_circ_0082580	TRIM24	7__138519189__138551180	0.397320783
4.560902571	1.33401676	0.4182193	3.18975418	0.001423939	0.019994626	hsa_circ_0006050	TMEM71	8__132727798__132757294	0.490737412
4.469864423	-2.3731902	0.74687387	-3.177498	0.001485517	0.020750938	hsa_circ_0002711	NRIP1	21__14991201__15043574	0.372875422
1.68671563	1.82643519	0.57625705	3.16948	0.00152712	0.021197552	hsa_circ_0078768	FAM120B	6__170317370__170323259	0.014994126
1.573961592	-1.1183064	0.35288238	-3.1690627	0.001529314	0.021197552	hsa_circ_0006764	MTMR1	X__150736595__150737448	0.131610793
1.735852869	1.83940639	0.58066188	3.16777533	0.001536102	0.021236903	hsa_circ_0088041	SUSD1	9__112080074__112098662	0.290281766
3.852815528	1.16664092	0.36943389	3.15791525	0.001589017	0.021744875	hsa_circ_0072546	GPBP1	5__57246300__57247215	0.226581904
1.00905482	-1.7617815	0.55803055	-3.1571417	0.001593239	0.021747309	hsa_circ_0006260	SLC41A2	12__104927973__104928694	0.403952218
3.465097981	3.41578541	1.08232079	3.15598245	0.001599585	0.021778651	hsa_circ_0021350	SOX6	11__16183886__16186955	0.61777728
1.34081241	1.71721838	0.54604529	3.14482774	0.001661847	0.022399525	hsa_circ_0006952	SMAD4	18__51054781__51067187	0.122630573
0.63768936	-1.9404623	0.61783178	-3.1407616	0.001685091	0.022656052	hsa_circ_0000350	AMOTL1	11__94795011__94800311	0.436221704
1.321043856	1.73013506	0.55107338	3.13957289	0.001691943	0.022689724	NA	GOLGA1	9__124923095__124938866	0.076792424
1.751910289	1.69241117	0.53975562	3.13551375	0.001715534	0.022858838	hsa_circ_0001555	STK10	5__172055588__172057473	0.195806529
5.209752093	1.56695017	0.5008142	3.12880536	0.001755185	0.023307128	hsa_circ_0000303	SPI1	11__47358844__47375729	0.474901318
3.387717214	3.39234593	1.08505196	3.12643639	0.001769388	0.023380264	NA	KLRC4-KLRK1	12__10386903__10393867	0.471251882
1.863410278	2.54294841	0.81380344	3.12476978	0.001779443	0.023455497	NA	LYST	1__235787200__235801097	0.354367703
1.537595803	1.72778265	0.55324052	3.12302261	0.00179004	0.023480085	hsa_circ_0004973	MTO1	6__73480675__73482244	0.246357794
6.144141948	1.55839513	0.4994563	3.12018311	0.001807386	0.023649938	hsa_circ_0000397	SLC38A1	12__46229153__46254936	0.745071511
3.871493514	1.38707413	0.44512092	3.11617375	0.001832143	0.02391569	NA	ZGRF1	4__112562371__112589874	0.23253692
2.884545678	-1.4364698	0.46183369	-3.1103616	0.001868584	0.024273543	hsa_circ_0018401	SGMS1	10__50433476__50590247	0.101444572

0.781574271	-1.7646578	0.56729247	-3.1106667	0.001866655	0.024273543	hsa_circ_0007064	INTS2	17__61919400__61925099	0.217327412
1.655478077	-1.2355418	0.39752812	-3.1080613	0.00188319	0.024404326	NA	UBAP2L	1__154234591__154235291	0.14727092
2.778831568	-1.1052278	0.35590824	-3.1053729	0.001900393	0.024552806	hsa_circ_0005092	IPO7	11__9429674__9431003	0.239303769
0.872372548	-1.9095411	0.61530331	-3.1034144	0.001913016	0.024612917	NA	DNAH14	1__225079207__225097239	0.522870866
1.553163742	-1.5205817	0.49086152	-3.0977814	0.001949752	0.024998902	hsa_circ_0009117	RERE	1__8495063__8614686	0.205489374
0.772014804	-1.6108039	0.52005163	-3.0973923	0.001952313	0.024998902	NA	ST8SIA4	5__100886343__100895785	0.215105818
3.302746853	1.47945897	0.47797423	3.09526935	0.001966342	0.02511874	hsa_circ_0006804	GATAD2A	19__19492306__19492712	0.05358952
1.603551301	-1.2414798	0.40138499	-3.09299	0.001981508	0.025252489	hsa_circ_0004083	PRKACB	1__84179177__84214317	0.188827959
2.691587274	1.95073206	0.63111628	3.09092338	0.001995351	0.02536879	hsa_circ_0000205	WDR37	10__1072116__1096246	0.251135686
3.905197029	1.21194091	0.39273584	3.08589335	0.002029416	0.025741036	hsa_circ_0008450	CMTM3	16__66608309__66610003	0.2913532
2.1046525	-1.3032689	0.42275189	-3.0828222	0.002050476	0.025946969	NA	ATP8B4	15__50010845__50047464	0.452806724
3.727517424	1.30363214	0.42365334	3.07711991	0.002090112	0.026324643	hsa_circ_0007895	EYA3	1__28035544__28058094	0.019295448
1.215650464	-1.1927759	0.38857352	-3.0696273	0.002143261	0.02693097	hsa_circ_0028190	ANAPC7	12__110382843__110396452	0.181234321
2.272164667	-1.4013046	0.45704445	-3.0660138	0.002169333	0.026943814	NA	PLA2G4A	1__186893011__186911389	0.315688405
2.236371916	-1.6886637	0.55073125	-3.066221	0.00216783	0.026943814	NA	CEP70	3__138525490__138537347	0.603814582
1.214538671	-1.5349848	0.50045434	-3.0671824	0.002160869	0.026943814	hsa_circ_0001383	DLG1	3__197115927__197119530	0.109469081
0.662640142	-1.8876501	0.61535995	-3.0675543	0.002158182	0.026943814	hsa_circ_0087421	DAPK1	9__87698656__87700237	0.292518098
1.816244745	2.31194469	0.75436798	3.06474395	0.002178564	0.026996126	NA	DUSP1	5__172769575__172770306	0.323831989
3.174800841	-2.0824758	0.6799952	-3.062486	0.002195068	0.0271381	hsa_circ_0006491	SETBP1	18__44701175__44701832	0.676281525
0.911556783	-1.4242501	0.46522706	-3.0614086	0.002202982	0.027173486	NA	CCDC146	7__77274482__77280653	0.213025472
0.948642817	-1.3569512	0.44374948	-3.0579217	0.002228778	0.027428756	hsa_circ_0008031	CHD9	16__53267294__53268126	0.125914771
1.770581198	2.27398891	0.74484339	3.0529759	0.002265841	0.027821221	hsa_circ_0009126	ASAP1	8__130214556__130358143	0.535407029
2.797612734	-1.1116218	0.36422802	-3.0519942	0.002273265	0.027834189	hsa_circ_0002041	IBTK	6__82210814__82212793	0.329825031
2.970858951	2.83294151	0.9296909	3.04718645	0.002309944	0.028042614	hsa_circ_0067990	FNDC3B	3__172251260__172298787	0.35890433
1.097217858	-1.1706665	0.38447922	-3.0448109	0.002328267	0.028201391	hsa_circ_0000079	USP1	1__62443159__62448666	0.182191671
1.237017275	-1.3007729	0.42786256	-3.0401653	0.002364484	0.028511644	NA	KAT6B	10__74969660__74989112	0.201791658
0.802860527	-1.5499087	0.5100114	-3.0389688	0.002373895	0.028553075	hsa_circ_0023812	NARS2	11__78469247__78493195	0.398222164
0.700248529	-2.0100317	0.66154716	-3.03838	0.002378538	0.028553075	hsa_circ_0002532	ZC3H12B	X__65075474__65075912	0.460287423
3.693862901	-1.0601259	0.34963157	-3.0321228	0.002428404	0.029022124	NA	TET1	10__68644698__68647005	0.582188498
1.30691621	-1.5372936	0.50779239	-3.0274058	0.002466626	0.029413558	NA	APLF	2__68490190__68578003	0.378505757
0.657936905	-1.6812535	0.55553364	-3.0263757	0.002475046	0.029448664	NA	ZFAND4	10__45625951__45639963	0.249642838

2.790046236	3.1170393	1.0304145	3.0250344	0.002486048	0.029513423	NA	SLFN12L	17__35478075__35480195	0.303997538
1.579467943	-1.2609914	0.41694265	-3.0243762	0.002491464	0.029513423	hsa_circ_0067808	RSRC1	3__158122103__158203245	0.075378101
1.482836018	-1.0422591	0.34522652	-3.0190586	0.002535615	0.029839251	hsa_circ_0011450	S100PBP	1__32825313__32830067	0.134133168
2.56573478	-1.0659013	0.35359923	-3.0144333	0.002574598	0.030231855	hsa_circ_0031632	BAZ1A	14__34792775__34862322	-0.009417127
3.917566803	1.49460327	0.49631809	3.01138181	0.002600616	0.030470836	hsa_circ_0008210	ATG7	3__11358418__11426926	0.422081677
0.836114888	-1.6229034	0.53926249	-3.0094869	0.002616893	0.030594896	NA	EXOC6B	2__72465160__72480750	0.379637736
3.285368074	1.57032349	0.52227099	3.00672161	0.002640815	0.030773616	hsa_circ_0000682	PRKCB	16__24032136__24035547	0.440511901
1.35925762	-1.2315474	0.40964213	-3.0063984	0.002643624	0.030773616	hsa_circ_0003885	EXTL3	8__28713457__28718207	0.320907067
1.929662883	-1.2120949	0.40390205	-3.0009626	0.002691276	0.031193286	hsa_circ_0001330	TIMMDC1	3__119503532__119517315	0.219281671
1.321163358	1.72848378	0.57666588	2.99737478	0.002723157	0.031418694	hsa_circ_0003127	VMP1	17__59737453__59773885	0.037585008
0.908084038	-2.4477322	0.81643332	-2.9980797	0.002716866	0.031418694	hsa_circ_0046760	L3MBTL4	18__6237964__6312056	0.616451526
1.159908354	-1.3855185	0.46317776	-2.9913321	0.002777632	0.031919032	NA	ATP2C1	3__130967145__130969396	0.307514141
1.462156554	2.17937043	0.7295508	2.98727716	0.002814744	0.032217167	NA	CEP57L1	6__109140421__109146937	0.060238748
1.247357989	-1.3904881	0.46558512	-2.986539	0.002821548	0.032217167	hsa_circ_0079385	ZDHHC4	7__6585016__6585260	0.128084931
1.987637987	-1.2064817	0.40445942	-2.9829487	0.002854858	0.032391194	hsa_circ_0060611	ZMYND8	20__47262288__47283648	0.224250556
2.194924494	2.77009302	0.92918326	2.98121278	0.002871092	0.032450353	NA	SAMD3	6__130175841__130184623	0.49670285
1.584293489	-1.3272004	0.44552274	-2.9789734	0.002892159	0.032608034	hsa_circ_0001403	RFC1	4__39326563__39327756	0.053075578
0.91839451	-1.3712441	0.46042236	-2.9782308	0.002899176	0.032618763	hsa_circ_0005521	KCTD3	1__215586495__215595471	0.223173804
3.623313876	1.99838999	0.67203854	2.97362408	0.002943054	0.033017549	hsa_circ_0000390	FGD4	12__32607957__32611283	0.664365329
0.74628245	-1.5704596	0.53093373	-2.9579202	0.003097223	0.034448	NA	NDC1	1__53818971__53832591	0.158205097
1.943741439	1.82292167	0.61634649	2.95762483	0.003100192	0.034448	hsa_circ_0087385	AGTPBP1	9__85585463__85596449	0.500517572
4.67832083	-1.3224611	0.44756204	-2.9548108	0.003128609	0.034692079	hsa_circ_0001073	ACVR2A	2__147896301__147899898	0.694958462
0.975001415	-1.7058129	0.57761708	-2.9531899	0.003145084	0.034803008	NA	CEP290	12__88106675__88107098	0.089172308
7.655807894	-1.3407332	0.45439477	-2.9505913	0.003171663	0.035025057	hsa_circ_0004435	FANCL	2__58198594__58232112	0.27361922
2.509094043	1.83764003	0.62332793	2.9481112	0.00319722	0.035234941	hsa_circ_0000450	RHOF	12__121780872__121781192	0.328882942
1.283645233	-1.7008071	0.57736039	-2.9458326	0.003220866	0.035422942	hsa_circ_0004649	AKT3	1__243695591__243843282	0.521743966
3.901286576	1.28419956	0.43637564	2.94287638	0.003251782	0.035689971	hsa_circ_0003863	RAB27A	15__55223889__55234956	0.471292434
3.702995348	1.15742866	0.39370161	2.93986266	0.003283578	0.03596554	hsa_circ_0002102	RPL14	3__40461407__40462248	0.061040156
2.129278458	-1.1579692	0.39417415	-2.9377095	0.003306467	0.036142639	hsa_circ_0001028	ZNF638	2__71363153__71370005	0.066596055
0.613359608	-1.8960162	0.64611813	-2.9344729	0.003341147	0.036415929	hsa_circ_0057123	CDCA7	2__173358712__173359491	0.30375948
0.554267738	-2.5470176	0.86807037	-2.9341142	0.003345011	0.036415929	hsa_circ_0002494	SLC37A3	7__140334634__140337349	0.418483857

1.232288581	1.62701895	0.55476304	2.93281788	0.003359009	0.036420864	NA	APBB1IP	10__26500819__26511906	0.148943431
1.286175303	1.65795777	0.56528498	2.93295916	0.003357481	0.036420864	NA	METTL25	12__82386803__82403130	0.221270835
0.732876943	-1.560487	0.53283039	-2.9286749	0.003404103	0.036761579	hsa_circ_0017239	SDCCAG8	1__243316755__243378863	0.185897932
1.395319409	-1.6802122	0.57392607	-2.927576	0.003416156	0.036817808	hsa_circ_0000371	ARHGAP32	11__129123446__129124894	0.429318361
0.816220702	-1.4739463	0.50412973	-2.923744	0.00345849	0.03719952	NA	TPM4	19__16081913__16101409	0.325281906
0.903782845	-1.5539793	0.53352133	-2.9126845	0.003583364	0.03831279	hsa_circ_0060734	CSE1L	20__49066192__49070297	0.117408877
0.689084084	-1.5591022	0.53686175	-2.9041037	0.003683061	0.039068052	hsa_circ_0007612	ORC5	7__104136781__104165282	0.044621099
1.832463543	-1.0885332	0.37473128	-2.9048369	0.003674446	0.039068052	hsa_circ_0007209	ZFAT	8__134600436__134610655	0.198107356
4.222598951	1.01871949	0.35182101	2.89556188	0.003784804	0.03998954	hsa_circ_0003549	SEC31A	4__82871944__82875822	0.243035659
2.028983417	1.88122788	0.6521215	2.88478128	0.003916857	0.04098221	hsa_circ_0017077	LYST	1__235677091__235697272	0.492083227
0.557723222	-1.7844185	0.61895569	-2.8829503	0.003939696	0.041141135	NA	HLTF	3__149055303__149076047	0.280279109
3.638334534	1.08589421	0.37698849	2.8804439	0.003971156	0.041308732	hsa_circ_0006374	ARCN1	11__118581246__118584644	0.050361457
1.910526423	2.18225146	0.75778116	2.87979113	0.003979387	0.041308732	hsa_circ_0023903	PICALM	11__85981129__86031611	-0.00206419
2.834932401	1.38030539	0.48082892	2.87067883	0.004095914	0.042361208	hsa_circ_0003148	LIN54	4__82970327__82979006	0.039883461
1.861515168	-1.8399291	0.64217434	-2.8651552	0.004168049	0.043024506	hsa_circ_0000646	HDGFL3	15__83151215__83164075	0.66730873
0.603266662	-1.6750962	0.58607843	-2.8581434	0.004261277	0.043902585	hsa_circ_0061721	BRWD1	21__39218152__39218660	0.064459439
1.448515654	1.78915059	0.62974355	2.84107806	0.004496131	0.046057505	hsa_circ_0057105	PDK1	2__172568741__172596023	0.353219039
0.677760754	-1.7662026	0.62220493	-2.8386189	0.004530924	0.046062968	hsa_circ_0003321	IFTAP	11__36610081__36636117	0.094300154
1.841565527	2.53440498	0.8925082	2.83964336	0.0045164	0.046062968	NA	DCAF5	14__69122217__69149503	-0.028862337
1.391745511	1.71251133	0.60323272	2.83888999	0.004527076	0.046062968	NA	DIAPH1	5__141581915__141583615	0.200040165
0.539108434	-2.114878	0.74479119	-2.8395582	0.004517605	0.046062968	NA	PPP2R3B	X__345516__361590	0.058221623
2.498219224	1.68022936	0.59325407	2.8322256	0.004622522	0.046817184	hsa_circ_0039053	ITGAL	16__30499074__30499489	0.440626303
0.843061994	-1.2979333	0.4591129	-2.827046	0.004697958	0.047402663	NA	PANK3	5__168559032__168566266	0.197092513
0.944626935	-1.6907059	0.59897602	-2.8226604	0.004762699	0.047786928	hsa_circ_0067582	RASA2	3__141512163__141540609	0.255684414
1.233813001	-1.5751048	0.55798874	-2.8228254	0.004760249	0.047786928	hsa_circ_0073222	SSBP2	5__81615473__81650339	0.401001667
1.927658271	-1.1336426	0.40181429	-2.8213098	0.004782799	0.047899243	hsa_circ_0001369	KLHL24	3__183650296__183651276	0.401238105
1.130974041	-1.3902021	0.49328047	-2.8182792	0.00482818	0.048263855	NA	RAB1A	2__65090983__65098066	0.091821507
1.461562961	-1.2977165	0.46094769	-2.8153227	0.004872828	0.048350677	hsa_circ_0019171	EXOC6	10__92893349__92909631	0.219420163
0.856391779	-1.2788837	0.45416475	-2.8159025	0.004864042	0.048350677	NA	ZBTB11	3__101664538__101672213	0.046328296
2.485963766	-1.0003283	0.35573936	-2.8119696	0.004923916	0.048767626	hsa_circ_0000679	ABCC1	16__16124789__16125911	0.268819027
2.143742386	1.63156541	0.58047609	2.81073664	0.004942823	0.048864889	hsa_circ_0000681	PRKCB	16__23988508__24035547	0.36621205

1.20447071	-1.2730682	0.45360421	-2.8065617	0.005007332	0.049411803	hsa_circ_0002229	LPIN1	2__11765533__11767858	0.217259315
0.853026711	-3.2540129	1.15977021	-2.8057393	0.005020129	0.04944735	NA	ZNF208	19__21974729__21988909	0.379166649
1.562635863	-1.0362476	0.36957809	-2.8038663	0.005049384	0.049463724	hsa_circ_0047719	POLI	18__54271360__54277855	0.316763217
1.76864029	1.70942292	0.60954922	2.80440508	0.005040953	0.049463724	hsa_circ_0001770	KMT2C	7__152330601__152358675	0.147202641

Supplementary Table S4. The list of DE circRNAs between ClinSeq-AML samples and healthy MHP samples.

baseMean	log2FoldChange	lfcSE	stat	pvalue	padj	circbaseID	geneSymbol	circID
10.3642299	-1.489341125	0.178988208	-8.320889615	8.73E-17	3.42E-13	hsa_circ_0008952	ZDHHC21	9__14639896__14680162
13.27473106	6.416492015	0.956020715	6.711666298	1.92E-11	2.51E-08	hsa_circ_0001777	ESYT2	7__158788004__158799072
10.33147254	-1.264268061	0.186963585	-6.762108559	1.36E-11	2.51E-08	hsa_circ_0085439	TBC1D31	8__123077111__123105464
9.236284126	5.897639284	0.949250375	6.212943854	5.20E-10	5.10E-07	hsa_circ_0000778	EFCAB13	17__47402132__47414919
13.56671744	-1.593274952	0.264389844	-6.026233564	1.68E-09	1.26E-06	hsa_circ_0001524	FNIP1	5__131677703__131709272
41.863496	5.123533872	0.873745418	5.863874956	4.52E-09	2.53E-06	hsa_circ_0012152	RNF220	1__44411981__44412722
8.329470159	-1.070468636	0.187220454	-5.717690629	1.08E-08	4.70E-06	hsa_circ_0003441	TDRD3	13__60439688__60467379
9.720276734	5.487086462	0.973618179	5.635768291	1.74E-08	6.83E-06	NA	FLT3	13__28024861__28028288
8.481767591	5.2958466	0.962140229	5.504235702	3.71E-08	1.21E-05	NA	FLT3	13__28023350__28028288
3.246996537	-1.426550613	0.259144346	-5.504849465	3.69E-08	1.21E-05	NA	METTL6	3__15411245__15415942
37.32634368	1.678776124	0.309800715	5.418890407	6.00E-08	1.68E-05	hsa_circ_0006156	FNDC3B	3__172247533__172251541
6.968843693	5.49070352	1.010874628	5.431636493	5.58E-08	1.68E-05	hsa_circ_0001869	TUT7	9__86305192__86310017
6.93014161	5.481203196	1.014808379	5.401219886	6.62E-08	1.73E-05	hsa_circ_0000386	CCDC91	12__28225795__28259442
26.67117281	1.202109234	0.232070037	5.179941586	2.22E-07	4.58E-05	hsa_circ_0000175	ELK4	1__205616478__205623891
3.065842308	-1.911091889	0.384643727	-4.96847279	6.75E-07	0.000125967	hsa_circ_0005406	PHACTR4	1__28459085__28466535
3.361127864	4.434101385	0.896506865	4.945975939	7.58E-07	0.000134996	hsa_circ_0001508	MSH3	5__80775694__80792844
4.655877008	4.923835462	0.999921192	4.924223529	8.47E-07	0.000144351	NA	FLT3	13__28048275__28050222
6.22302019	-1.233184506	0.253626593	-4.862205073	1.16E-06	0.000189606	hsa_circ_0085440	TBC1D31	8__123077111__123109620
6.732353721	5.447082944	1.123694751	4.847475652	1.25E-06	0.000196066	hsa_circ_0004435	FANCL	2__58198594__58232112
25.1302017	1.190022781	0.248039849	4.797708047	1.60E-06	0.000241972	hsa_circ_0000799	BPTF	17__67945409__67975958
10.1431709	6.022487999	1.266821859	4.754013326	1.99E-06	0.000289525	hsa_circ_0002468	CEP70	3__138570318__138571356
8.493788703	-1.48344702	0.313443271	-4.732744824	2.22E-06	0.000299412	hsa_circ_0006677	DNAI4	1__66891154__66905375
3.867986043	4.17112232	0.881273953	4.733059799	2.21E-06	0.000299412	hsa_circ_0072780	CCDC125	5__69294793__69311204
16.10750021	-3.153631636	0.67037197	-4.704301159	2.55E-06	0.000330661	hsa_circ_0004771	NRIP1	21__15014344__15043574
3.777909108	4.616571555	0.984826576	4.687700014	2.76E-06	0.000338458	NA	FLT3	13__28014452__28015701
8.613054103	5.786085286	1.238026296	4.673636826	2.96E-06	0.000351508	hsa_circ_0004524	CEP70	3__138570318__138572932
2.756402118	4.173935	0.907099346	4.601408898	4.20E-06	0.000483823	NA	PARG	10__49915917__49935142
6.215116599	-1.320281843	0.288804497	-4.57154185	4.84E-06	0.000542246	hsa_circ_0002158	RERE	1__8541214__8557523

1.539189877	-2.281981176	0.500019045	-4.563788517	5.02E-06	0.000547043	NA	IQGAP2	5__76697987__76702590
3.474302331	4.483454355	1.003433637	4.468112479	7.89E-06	0.000793175	hsa_circ_0003700	FBXO9	6__53071057__53076543
4.794441601	-1.302170921	0.292656095	-4.449491889	8.61E-06	0.000822948	hsa_circ_0003501	GABPB1	15__50300789__50309798
5.31876194	5.085457269	1.142346329	4.45176488	8.52E-06	0.000822948	hsa_circ_0054597	RTN4	2__55025086__55027485
4.310434999	-1.179393254	0.26999961	-4.368129469	1.25E-05	0.001091635	hsa_circ_0005552	EHBP1	2__62979188__62996766
3.598830516	4.545889169	1.042497914	4.360573874	1.30E-05	0.001105455	NA	FLT3	13__28018467__28028288
5.553865996	4.664964877	1.073974071	4.343647583	1.40E-05	0.001144446	hsa_circ_0052318	ZNF418	19__57925623__57928047
3.465872767	-1.134882855	0.263465801	-4.307514872	1.65E-05	0.001320792	NA	ZNF638	2__71426460__71427414
3.227692479	4.404150252	1.03257259	4.26522096	2.00E-05	0.001565683	hsa_circ_0084606	ASPH	8__61618978__61653660
8.102150107	4.732729142	1.112875638	4.252702621	2.11E-05	0.001623387	hsa_circ_0002805	ZBTB46	20__63775678__63790790
2.548727435	4.04815672	0.954713439	4.240179886	2.23E-05	0.001683646	NA	EMC2	8__108449823__108450492
15.18340619	1.115453062	0.267725555	4.1664049	3.09E-05	0.002166086	hsa_circ_0005600	YPEL2	17__59353215__59353526
10.97601198	1.58936317	0.381299327	4.168282125	3.07E-05	0.002166086	hsa_circ_0001756	HIPK2	7__139715932__139717015
11.58503728	-1.149992431	0.280129239	-4.105220993	4.04E-05	0.002683729	hsa_circ_0001839	KDM4C	9__6880012__6893232
3.51586077	4.50898765	1.103153038	4.087363671	4.36E-05	0.002850512	hsa_circ_0000586	UBE3A	15__25405461__25411971
3.3621157	3.510942173	0.860038841	4.082306528	4.46E-05	0.002865515	hsa_circ_0000068	GPBP1L1	1__45659028__45661225
9.454682287	-1.148069193	0.282372676	-4.06579422	4.79E-05	0.002997043	NA	RERE	1__8655973__8656441
4.664199295	4.422668235	1.088161608	4.064348717	4.82E-05	0.002997043	hsa_circ_0067991	FNDC3B	3__172251260__172307501
2.146389869	3.788980668	0.938592746	4.036874014	5.42E-05	0.003317798	hsa_circ_0087493	IARS1	9__92256680__92285839
2.848383079	4.223173626	1.04724383	4.032655532	5.52E-05	0.003325975	hsa_circ_0055945	UXS1	2__106122970__106166083
6.50003698	1.678463079	0.420540577	3.991203632	6.57E-05	0.003904487	hsa_circ_0000362	CBL	11__119273868__119274953
8.690687842	1.550256757	0.390103453	3.973963176	7.07E-05	0.004015813	hsa_circ_0005986	PRDM2	1__13731000__13742157
4.792229522	4.926188232	1.239467909	3.974437899	7.05E-05	0.004015813	hsa_circ_0068606	TFRC	3__196053418__196077122
2.689709799	3.627599172	0.912084869	3.977260556	6.97E-05	0.004015813	hsa_circ_0004338	CAMSAP1	9__135850137__135883078
9.090852044	1.219200633	0.30874575	3.94888232	7.85E-05	0.004396948	hsa_circ_0001861	GRHPR	9__37424845__37426654
2.404122083	3.484250263	0.885548478	3.934567503	8.33E-05	0.004551612	NA	NCOA1	2__24643966__24673463
50.97426862	2.897881088	0.736655398	3.933835406	8.36E-05	0.004551612	hsa_circ_0001947	AFF2	X__148661908__148662768
1.857866178	3.586947247	0.916240663	3.914852714	9.05E-05	0.004807058	NA	ME2	18__50918110__50940386
3.871401433	-1.320377301	0.337339463	-3.914090832	9.07E-05	0.004807058	hsa_circ_0008501	RERE	1__8541214__8614686
1.056488569	-1.770414623	0.455091411	-3.890239591	0.000100145	0.005032943	hsa_circ_0088369	RC3H2	9__122853952__122855878
9.56605558	1.266215245	0.326671255	3.876114669	0.000106138	0.005200746	hsa_circ_0001789	RAB11FIP1	8__37877109__37877551

1.891755639	3.632816684	0.941341681	3.859190301	0.000113763	0.005505583	hsa_circ_0001028	ZNF638	2__71363153__71370005
4.493163506	-1.161199375	0.303712226	-3.823354067	0.000131649	0.006221791	NA	ZNF525	19__53380722__53384056
29.83959573	1.08155864	0.288308093	3.751398824	0.000175851	0.008015521	hsa_circ_0001868	AGTPBP1	9__85618983__85633374
1.654105205	3.428237104	0.915310475	3.745436329	0.000180081	0.008113973	hsa_circ_0068738	LRCH3	3__197830770__197866219
2.256533811	3.834133716	1.02782481	3.730337776	0.000191223	0.008455797	hsa_circ_0008679	CCDC7	10__32565558__32584304
18.04979694	-1.157758096	0.310445747	-3.729341137	0.000191981	0.008455797	hsa_circ_0000419	RAB3IP	12__69800209__69801721
1.594886177	3.351243349	0.90594139	3.699183396	0.000216294	0.009290093	hsa_circ_0004451	ENSG00000226899:<br>ENSG00000249456	10__124942457__124962408
6.769835609	-3.2990312	0.892317178	-3.697150835	0.000218033	0.009290093	hsa_circ_0067735	MED12L	3__151116338__151127984
4.100243662	4.724258722	1.284154367	3.678886933	0.000234254	0.009768894	NA	VRK2	2__58084089__58089723
2.169918915	3.326280503	0.907089576	3.666981291	0.000245431	0.010127248	hsa_circ_0084620	YTHDF3	8__63186147__63187745
2.865914481	4.203242298	1.152739399	3.646307484	0.000266036	0.010547372	hsa_circ_0054598	RTN4	2__55025086__55028220
3.092535055	4.304500619	1.185200908	3.631874217	0.00028137	0.010920509	hsa_circ_0008638	USP7	16__8920359__8930397
1.669137676	3.446620794	0.953549823	3.614515689	0.00030091	0.011452093	hsa_circ_0004746	VMP1	17__59737453__59817773
8.397913065	-1.583576752	0.438076267	-3.614842601	0.00030053	0.011452093	hsa_circ_0000099	AMY2B	1__103565435__103575540
1.744561153	3.474388486	0.968345371	3.587964159	0.00033327	0.012209521	NA	MGA	15__41668828__41713496
3.005406625	4.282108947	1.192842472	3.589836082	0.000330886	0.012209521	hsa_circ_0015259	SUCO	1__172551512__172570730
3.913715824	3.204810876	0.891418517	3.595180954	0.000324166	0.012209521	hsa_circ_0002617	KANSL1L	2__210104104__210154611
5.002064321	-1.301053113	0.362136121	-3.592718422	0.000327246	0.012209521	hsa_circ_0001470	GOLPH3	5__32135572__32143880
1.042041669	-1.934074214	0.539455306	-3.585235312	0.000336774	0.012223664	NA	RABGAP1	9__122957011__123020459
17.7216478	-2.146344861	0.601459847	-3.568558849	0.00035895	0.012879616	hsa_circ_0031583	ARHGAP5	14__32090502__32094386
1.726139579	3.457538131	0.973432803	3.551902218	0.000382457	0.012886595	NA	ATP8B4	15__50010845__50047464
1.824114732	3.095394675	0.871840424	3.550414261	0.000384625	0.012886595	hsa_circ_0007983	GABPB1	15__50300789__50304133
1.795127678	3.532474028	0.993193978	3.556680876	0.00037557	0.012886595	hsa_circ_0005946	RPA1	17__1842803__1844686
1.510161411	3.253161664	0.912921142	3.563464046	0.000365993	0.012886595	NA	FAM13B	5__137985257__138019146
13.61245722	-1.741254025	0.488859367	-3.561871048	0.000368221	0.012886595	hsa_circ_0001686	PALS2	7__24623666__24668660
2.282773081	3.419345555	0.96074657	3.559050493	0.000372198	0.012886595	NA	DNAAF11	8__132622611__132661627
2.133286452	3.804213805	1.070756092	3.552829474	0.000381111	0.012886595	hsa_circ_0008812	RAD23B	9__107300141__107311737
2.73664134	3.662630788	1.034063813	3.54197753	0.000397139	0.013082236	NA	ITPR2	12__26481131__26655852
6.452231865	4.891447735	1.380763458	3.542567489	0.000396252	0.013082236	hsa_circ_0003692	FNDC3B	3__172251260__172310881
3.952241788	-1.32134642	0.373887222	-3.534077502	0.000409201	0.013367235	hsa_circ_0001641	HBS1L	6__135039573__135042126
1.5850429	3.357624751	0.954232413	3.518665585	0.000433723	0.014051193	hsa_circ_0002634	ATXN7	3__63898399__63952483

1.554642745	3.333491462	0.952740066	3.498846729	0.000467275	0.01489202	hsa_circ_0012856	RAVER2	1__64777623__64789514
2.016575488	-1.404277668	0.401950786	-3.493655736	0.000476455	0.015062126	hsa_circ_0081872	SYPL1	7__106092949__106099282
1.469397145	-1.950718361	0.562988631	-3.464933842	0.000530362	0.016370227	hsa_circ_0054214	MAP4K3	2__39336920__39337581
2.22920406	3.35801631	0.979687472	3.427640351	0.000608851	0.018219067	hsa_circ_0007271	RAD23B	9__107300141__107325004
1.402748779	3.189509102	0.931939132	3.422443582	0.00062061	0.018430229	NA	CDK13	7__39987599__39997664
5.979060098	1.703992617	0.49907217	3.414321052	0.000639412	0.018845822	hsa_circ_0004861	PRDM2	1__13731000__13749487
1.952830805	3.188607213	0.935064899	3.410038401	0.000649537	0.019001391	NA	LRMDA	10__76036008__76058783
4.493103064	-1.068991715	0.314908709	-3.394608289	0.000687269	0.019956242	hsa_circ_0003162	BBS9	7__33146242__33177591
2.357017945	3.429186578	1.012246017	3.387700739	0.000704811	0.020315146	hsa_circ_0006840	TRA2B	3__185921104__185922126
8.160184767	1.470791906	0.434620714	3.384081472	0.000714168	0.020369228	hsa_circ_0001882	ECPAS	9__111386377__111391824
1.697825596	3.465490457	1.025708034	3.37863246	0.000728473	0.020543995	NA	ATP8B4	15__49876278__49934182
1.762122226	3.077275213	0.912584249	3.372045063	0.000746122	0.020743261	hsa_circ_0032821	CEP128	14__80743075__80761613
1.732697242	3.49842857	1.03692725	3.373841867	0.000741269	0.020743261	NA	ATP8B4	15__49916934__49962020
2.495231559	3.06224047	0.917192764	3.338709801	0.000841684	0.023072748	hsa_circ_0003051	ENSG00000140006:<br>ENSG00000258800	14__63598756__63599973
1.81379327	3.042766514	0.913858266	3.329582524	0.000869763	0.023676878	hsa_circ_0042881	NF1	17__31155983__31182665
2.703121568	3.151634091	0.947219819	3.327246778	0.000877087	0.023711583	hsa_circ_0003059	MED13L	12__116096669__116237705
1.790512224	3.037928971	0.920089821	3.301774352	0.000960753	0.025620087	hsa_circ_0001952	MPP1	X__154789954__154792285
2.058163071	3.251644819	0.98610411	3.297466046	0.000975615	0.025667179	hsa_circ_0058010	KANSL1L	2__210098086__210154611
5.265694618	-2.077424414	0.629893975	-3.298054113	0.000973574	0.025667179	NA	PALS2	7__24620052__24668660
6.570222397	-1.16555949	0.353877432	-3.293681326	0.000988845	0.025670688	hsa_circ_0004592	STK3	8__98706467__98707311
10.63328143	1.151340676	0.350960599	3.280541119	0.001036082	0.026719999	hsa_circ_0000754	SSH2	17__29684563__29703062
1.55817415	3.323770375	1.015003418	3.274639589	0.001057969	0.027106123	hsa_circ_0087243	VPS13A	9__77214329__77227485
4.070965625	2.160820601	0.660412314	3.271926576	0.001068173	0.027189861	NA	ZGRF1	4__112562371__112589874
1.350402011	3.129915109	0.959737582	3.2612197	0.001109341	0.028055579	hsa_circ_0081840	PUS7	7__105459168__105495253
1.794755823	3.093972997	0.950852271	3.253894525	0.001138345	0.028604576	NA	NCOA1	2__24643966__24683128
4.908368877	-1.075528148	0.331617366	-3.243280534	0.001181618	0.029257752	hsa_circ_0003788	PRIMPOL	4__184659340__184678394
2.381102346	-1.043367878	0.32194891	-3.240787111	0.001192002	0.029257752	hsa_circ_0005684	DENND4C	9__19286769__19305527
6.113119196	2.604497024	0.807052708	3.227170911	0.001250208	0.030439837	hsa_circ_0001361	FNDC3B	3__172112452__172133546
2.854548704	3.264678187	1.014099065	3.219289218	0.001285088	0.030943587	hsa_circ_0000431	DRAM1	12__101908186__101914232
1.428931286	3.193168269	0.991995838	3.218933131	0.001286685	0.030943587	hsa_circ_0058876	ILKAP	2__238182065__238194870
1.333167891	3.091366831	0.961953148	3.213635547	0.001310659	0.031327951	NA	MYB	6__135192324__135200415

2.105725808	2.855474412	0.890629202	3.206131581	0.001345325	0.03196165	NA	SENP2	3__185598412__185613408
1.248234369	-1.683387731	0.525516037	-3.203304205	0.001358604	0.032082694	hsa_circ_0008790	CLNS1A	11__77622500__77625818
7.632365398	1.38148379	0.432315216	3.195547455	0.001395658	0.032760362	hsa_circ_0005899	LYST	1__235830226__235833667
5.577239192	-1.04508421	0.327575617	-3.190360198	0.001420956	0.033155633	NA	ZZZ3	1__77631850__77641655
1.307102809	3.107241702	0.976663717	3.181485755	0.001465217	0.033847761	hsa_circ_0002999	BARD1	2__214752447__214797117
4.171001657	-1.140631877	0.358581211	-3.180958293	0.001467888	0.033847761	NA	DPY19L1P1	7__32632543__32679130
8.786961223	1.049365316	0.332009104	3.160652235	0.001574163	0.034794189	hsa_circ_0000442	MED13L	12__116230433__116237705
3.393818506	-1.07819324	0.340215832	-3.169144818	0.001528882	0.034794189	hsa_circ_0004815	PPHLN1	12__42351885__42398994
1.419166745	3.214366974	1.014747849	3.167650935	0.001536759	0.034794189	hsa_circ_0005036	ETFA	15__76283757__76292700
1.364572976	3.184007756	1.008151082	3.158264482	0.001587115	0.034794189	hsa_circ_0003101	SLC25A26	3__66236544__66263379
10.36256137	-1.018213467	0.321654832	-3.165546937	0.001547917	0.034794189	hsa_circ_0005954	AMD1	6__110887505__110890356
6.44522086	1.143707221	0.361653173	3.162442102	0.001564518	0.034794189	hsa_circ_0079929	CDK13	7__39987599__39988258
2.801721986	-1.269233855	0.401916746	-3.157952159	0.001588816	0.034794189	hsa_circ_0085441	TBC1D31	8__123077111__123120188
1.632738634	2.937022565	0.940080963	3.124222998	0.001782753	0.038763124	hsa_circ_0002223	ATG2B	14__96341522__96347341
1.674552788	2.954725598	0.946252417	3.122555405	0.001792884	0.038763124	hsa_circ_0000697	PHKB	16__47463899__47515601
1.666736504	-1.378866151	0.442749455	-3.114326026	0.001843656	0.039492532	hsa_circ_0006764	MTMR1	X__150736595__150737448
1.993436028	3.190567363	1.027260095	3.105900227	0.001897007	0.040196043	hsa_circ_0004113	SLC25A26	3__66243203__66263379
1.345335773	3.100640893	0.999705135	3.101555434	0.001925068	0.040354372	hsa_circ_0064772	CLASP2	3__33592395__33644903
5.62427687	-1.017345814	0.328233131	-3.09946108	0.00193873	0.040424593	hsa_circ_0001700	COA1	7__43639449__43640649
1.572072644	2.879620711	0.931467018	3.091489721	0.001991549	0.040818421	hsa_circ_0008710	PTPN2	18__12814203__12836891
8.717543515	-1.875050051	0.606031245	-3.093982474	0.001974892	0.040818421	hsa_circ_0002454	DNAJC6	1__65364635__65366196
2.570461491	2.627181085	0.849776315	3.091614861	0.001990709	0.040818421	hsa_circ_0057051	DCAF17	2__171448681__171476950
0.886692283	-2.751537787	0.890367108	-3.090340785	0.00199927	0.040818421	hsa_circ_0079449	TMEM106B	7__12224226__12229819
15.29617026	-1.140934308	0.370186238	-3.082054902	0.002055769	0.041115383	hsa_circ_0001009	FANCL	2__58221942__58232112
1.074273849	2.824181758	0.915979552	3.083236684	0.002047622	0.041115383	hsa_circ_0007473	CCT4	2__61873002__61883548
1.633014905	-1.843571933	0.599869553	-3.073288059	0.00211714	0.042037619	hsa_circ_0005348	ZC3H12C	11__110136663__110137414
5.829822993	-1.409853365	0.458874353	-3.072417004	0.002123329	0.042037619	hsa_circ_0001685	PALS2	7__24623666__24650712
1.850000416	2.644971813	0.864835761	3.058351576	0.002225583	0.043621426	hsa_circ_0074082	FAM13B	5__138006990__138019146
4.10094905	2.285701559	0.747866064	3.056298003	0.002240884	0.043702815	hsa_circ_0004550	LYST	1__235793503__235801097
1.346229224	3.136601137	1.029043045	3.048075734	0.002303119	0.043826337	NA	FLT3	13__28037185__28052674
1.700085702	3.00105932	0.984104735	3.049532447	0.002291979	0.043826337	hsa_circ_0005039	RERE	1__8614561__8656441

1.542728797	3.329912171	1.092105433	3.049075731	0.002295466	0.043826337	hsa_circ_0001168	CSE1L	20__49074785__49091022
4.767101434	3.020499197	0.989307507	3.053144926	0.002264565	0.043826337	hsa_circ_0008210	ATG7	3__11358418__11426926
1.161237458	2.933431642	0.961739624	3.050130792	0.002287417	0.043826337	hsa_circ_0005388	TCP1	6__159784666__159788143
1.522044294	2.85561392	0.939023108	3.041047547	0.002357566	0.044645694	hsa_circ_0001325	BBX	3__107710452__107733023
1.155237352	2.936713305	0.968013407	3.033752717	0.002415323	0.045519553	hsa_circ_0004050	PKD2	4__88038251__88065879
1.499081285	3.300248004	1.089339118	3.029587343	0.002448881	0.045931162	hsa_circ_0004469	RBX1	22__40953555__40967884
1.657086421	2.963909535	0.980768197	3.022028594	0.002510868	0.046759794	hsa_circ_0008542	PHF12	17__28921688__28927063
1.407447998	2.69573065	0.892634753	3.019970533	0.002527993	0.046759794	hsa_circ_0011450	S100PBP	1__32825313__32830067
1.378410275	3.190744995	1.05775526	3.01652482	0.002556903	0.047056616	NA	CCDC171	9__15563978__15594172
1.802400677	3.520317374	1.173269028	3.00043493	0.002695943	0.048496846	hsa_circ_0023903	PICALM	11__85981129__86031611
1.565657808	3.350893892	1.116312336	3.001752989	0.002684299	0.048496846	hsa_circ_0004463	RPAP2	1__92333391__92345914
8.254672594	1.250902006	0.416923765	3.000313513	0.002697018	0.048496846	hsa_circ_0001112	DGKD	2__233388257__233390483
15.41432819	1.55581961	0.518849225	2.998596773	0.00271226	0.048548217	hsa_circ_0008934	ASAP1	8__130152736__130180880
6.177271054	1.543234687	0.514999497	2.996575137	0.002730309	0.04864915	NA	BAZ1A	14__34800224__34802988
1.319480849	3.130594526	1.048664454	2.985315763	0.002832857	0.049582333	NA	ZMYND11	10__179994__249088
1.635925699	2.889464886	0.968349238	2.983907844	0.002845925	0.049582333	hsa_circ_0036044	PIAS1	15__68141946__68164804
1.533249421	2.791976996	0.935580471	2.984218978	0.002843032	0.049582333	hsa_circ_0043379	PIP4K2B	17__38777687__38786920
2.066701924	2.742018652	0.917958852	2.987082313	0.002816539	0.049582333	hsa_circ_0014584	ASH1L	1__155395459__155459898
2.585753982	3.070300642	1.027229004	2.988915452	0.002799696	0.049582333	hsa_circ_0016601	DNAH14	1__224952670__225007544

Supplementary Table S5. Multivariable Cox regression analysis for circRNA 11__94583448__94608.

	coef	exp(coef)	se(coef)	z	Pr(>|z|)
11__94583448__94608686	-0.2537	0.775926	0.154535	-1.64168	0.100656
age	0.038829	1.039593	0.006151	6.312377	2.75E-10
sex	-0.03465	0.96594	0.143608	-0.24131	0.809318
FLT3	0.63709	1.89097	0.19276	3.305101	0.000949
NPM1	-0.41945	0.657409	0.207056	-2.02578	0.042788
TP53	0.772807	2.165836	0.221946	3.481963	0.000498
CBFB.MYH11	0.663641	1.94185	0.544009	1.219907	0.2225
PML.RARA	-17.1213	3.67E-08	1880.126	-0.00911	0.992734
RUNX1.RUNX1T1	0.450011	1.56833	0.609593	0.738217	0.460383
MLL	0.233048	1.262442	0.732356	0.318217	0.75032
inv.3	-0.12858	0.879343	0.594433	-0.21631	0.828748
ELN2022Favorable	-0.19115	0.826008	0.211598	-0.90336	0.366333
ELN2022Intermediate	-0.02043	0.979775	0.228423	-0.08945	0.928725

Supplementary Table S6. Multivariable Cox regression analysis for circRNA 8__67118248__67137603.

	coef	exp(coef)	se(coef)	z	Pr(>|z|)
8__67118248__67137603	-0.5486	0.577759	0.146503	-3.74462	0.000181
age	0.041135	1.041993	0.006242	6.589654	4.41E-11
sex	0.039504	1.040295	0.145649	0.27123	0.786214
FLT3	0.66912	1.952517	0.188306	3.55336	0.00038
NPM1	-0.48755	0.614131	0.202778	-2.40434	0.016202
TP53	0.883677	2.41978	0.217732	4.058543	4.94E-05
CBFB.MYH11	0.717751	2.049819	0.547153	1.311792	0.18959
PML.RARA	-17.2282	3.30E-08	1903.371	-0.00905	0.992778
RUNX1.RUNX1T1	0.744239	2.104839	0.617149	1.205931	0.227844
MLL	0.466927	1.595085	0.733948	0.636185	0.524656
inv.3	-0.18333	0.832496	0.598464	-0.30633	0.759355
ELN2022Favorable	-0.13596	0.872875	0.22074	-0.61594	0.537934
ELN2022Intermediate	-0.06833	0.933952	0.232293	-0.29416	0.768639

Supplementary Table S7. Survival analysis of splice transcripts of PIWIL4, the host gene of 11__94583448__94608.

CircRNA	Gene	Transcript	coef	exp(coef)	se(coef)	z	Pvalue	FDR
11__94583448__94608686	PIWIL4	ENST00000543336	-0.341395082	0.710778037	0.134725879	-2.533998	0.011277	0.056385
11__94583448__94608686	PIWIL4	ENST00000299001	-0.28806094	0.749715904	0.135594844	-2.124424	0.033635	0.063552
11__94583448__94608686	PIWIL4	ENST00000446230	-0.278985159	0.756551131	0.134551805	-2.07344	0.038131	0.063552
11__94583448__94608686	PIWIL4	ENST00000545603	-0.141336531	0.868197087	0.142857018	-0.989357	0.322489	0.403111
11__94583448__94608686	PIWIL4	ENST00000537419	-0.108704867	0.896995112	0.13385392	-0.812116	0.416725	0.416725

Supplementary Table S8. Survival analysis of splice transcripts of CSPP1, the host gene of 8__67118248__67137603.

CircRNA	Gene	Transcript	coef	exp(coef)	se(coef)	z	Pvalue	FDR
8__67118248__67137603	CSPP1	ENST00000262210	1.03264355	2.808480388	0.289067763	3.572323451	0.000353828	0.026890929
8__67118248__67137603	CSPP1	ENST00000676113	0.598183992	1.818812821	0.194234289	3.079703352	0.002072069	0.060187681
8__67118248__67137603	CSPP1	ENST00000676968	0.947966487	2.580456928	0.311962094	3.03872331	0.00237583	0.060187681
8__67118248__67137603	CSPP1	ENST00000678138	0.395430009	1.485022626	0.193759782	2.040826037	0.041268122	0.646404158
8__67118248__67137603	CSPP1	ENST00000677697	-0.606441283	0.545287946	0.309555864	-1.959068955	0.050104712	0.646404158
8__67118248__67137603	CSPP1	ENST00000677845	-0.425130418	0.653684528	0.220480473	-1.928199864	0.053830271	0.646404158
8__67118248__67137603	CSPP1	ENST00000678204	-0.262327815	0.769258808	0.139224653	-1.884205195	0.059537225	0.646404158
8__67118248__67137603	CSPP1	ENST00000676980	-0.371052344	0.690007823	0.205456094	-1.805993374	0.070919375	0.657813916
8__67118248__67137603	CSPP1	ENST00000676567	-0.270672038	0.762866647	0.161781421	-1.673072447	0.094313035	0.657813916
8__67118248__67137603	CSPP1	ENST00000674647	0.23315925	1.262582529	0.140470074	1.659849982	0.096944636	0.657813916
8__67118248__67137603	CSPP1	ENST00000678362	-0.272956018	0.761126263	0.165360341	-1.650674017	0.098805156	0.657813916
8__67118248__67137603	CSPP1	ENST00000521168	0.302145623	1.352758206	0.186530548	1.619818456	0.105271281	0.657813916
8__67118248__67137603	CSPP1	ENST00000677052	0.208703619	1.23207978	0.134016397	1.557299134	0.119399481	0.657813916
8__67118248__67137603	CSPP1	ENST00000677430	0.263607025	1.301616593	0.188756605	1.39654464	0.162550548	0.657813916
8__67118248__67137603	CSPP1	ENST00000678345	-0.186206996	0.83010175	0.134220794	-1.387318541	0.165344652	0.657813916
8__67118248__67137603	CSPP1	ENST00000679060	-0.183261239	0.832550633	0.13434917	-1.364066774	0.172546551	0.657813916
8__67118248__67137603	CSPP1	ENST00000678685	0.262446672	1.300107134	0.196359588	1.336561529	0.181365804	0.657813916
8__67118248__67137603	CSPP1	ENST00000676471	-1.326088754	0.265513723	1.002515328	-1.322761575	0.185914679	0.657813916
8__67118248__67137603	CSPP1	ENST00000676804	-0.177036788	0.837748965	0.134182747	-1.319370723	0.187045204	0.657813916
8__67118248__67137603	CSPP1	ENST00000679112	-0.29063089	0.747791645	0.22087522	-1.315814831	0.1882362	0.657813916
8__67118248__67137603	CSPP1	ENST00000521919	-1.310797116	0.269605064	1.003733515	-1.305921439	0.191579275	0.657813916
8__67118248__67137603	CSPP1	ENST00000679226	0.312923921	1.367417496	0.2405788	1.300712784	0.193356785	0.657813916
8__67118248__67137603	CSPP1	ENST00000677938	-0.170823799	0.842970093	0.134177528	-1.273117798	0.202976244	0.657813916
8__67118248__67137603	CSPP1	ENST00000521324	-0.240853799	0.785956526	0.191179609	-1.259829957	0.20773071	0.657813916
8__67118248__67137603	CSPP1	ENST00000675820	-0.163632265	0.849054191	0.133970285	-1.22140716	0.221931898	0.674672969
8__67118248__67137603	CSPP1	ENST00000677619	-0.30160311	0.739631559	0.276209087	-1.091937679	0.274860495	0.757071977
8__67118248__67137603	CSPP1	ENST00000678444	0.194455709	1.214649683	0.180214659	1.079022703	0.280577607	0.757071977
8__67118248__67137603	CSPP1	ENST00000678216	-0.189975769	0.826979172	0.176274338	-1.077727884	0.281155211	0.757071977

8__67118248__67137603	CSPP1	ENST00000676656	0.139172736	1.149322612	0.13381679	1.040024467	0.298328534	0.757071977
8__67118248__67137603	CSPP1	ENST00000676573	0.399462601	1.491023208	0.384499738	1.038915146	0.298844202	0.757071977
8__67118248__67137603	CSPP1	ENST00000676695	-0.128477059	0.879433737	0.133986956	-0.958877362	0.33762053	0.795214589
8__67118248__67137603	CSPP1	ENST00000676605	-0.144450697	0.865497583	0.15458996	-0.934411893	0.350091465	0.795214589
8__67118248__67137603	CSPP1	ENST00000678156	-0.121951235	0.885191534	0.134089846	-0.90947405	0.36309995	0.795214589
8__67118248__67137603	CSPP1	ENST00000678553	-0.347873343	0.706188314	0.384252023	-0.905325989	0.365292712	0.795214589
8__67118248__67137603	CSPP1	ENST00000677836	-0.3474686	0.706474197	0.384545855	-0.903581707	0.366217245	0.795214589
8__67118248__67137603	CSPP1	ENST00000678744	-0.137356148	0.871659731	0.158440926	-0.866923414	0.385983974	0.814855055
8__67118248__67137603	CSPP1	ENST00000678747	-0.336995696	0.713911913	0.41398228	-0.814034108	0.415625412	0.853717062
8__67118248__67137603	CSPP1	ENST00000677131	-0.120499757	0.886477303	0.156388882	-0.770513575	0.440995305	0.865039199
8__67118248__67137603	CSPP1	ENST00000679295	-0.098888316	0.905843871	0.134204339	-0.73684887	0.46121426	0.865039199
8__67118248__67137603	CSPP1	ENST00000676858	0.261590652	1.298994693	0.360333526	0.725968119	0.467858315	0.865039199
8__67118248__67137603	CSPP1	ENST00000678017	0.206740056	1.229662886	0.286876814	0.720657946	0.471119993	0.865039199
8__67118248__67137603	CSPP1	ENST00000676534	-0.08982729	0.914089044	0.133822909	-0.671240004	0.502067648	0.865039199
8__67118248__67137603	CSPP1	ENST00000677964	0.084076037	1.087711597	0.133724081	0.628727722	0.529527326	0.865039199
8__67118248__67137603	CSPP1	ENST00000678927	-0.082178025	0.921107964	0.133900587	-0.613724158	0.539397622	0.865039199
8__67118248__67137603	CSPP1	ENST00000675990	-0.080713266	0.922458153	0.133963701	-0.602501019	0.546840685	0.865039199
8__67118248__67137603	CSPP1	ENST00000676882	0.129647458	1.13842697	0.216680252	0.598335369	0.549616181	0.865039199
8__67118248__67137603	CSPP1	ENST00000678728	0.079848854	1.083123345	0.133691884	0.597260293	0.550333611	0.865039199
8__67118248__67137603	CSPP1	ENST00000678318	-0.158893657	0.853087074	0.267946168	-0.593005894	0.553177208	0.865039199
8__67118248__67137603	CSPP1	ENST00000678723	0.104653894	1.110326253	0.178520955	0.586227503	0.557722642	0.865039199
8__67118248__67137603	CSPP1	ENST00000677592	0.083594167	1.087187587	0.148794297	0.561810289	0.574245281	0.872852827
8__67118248__67137603	CSPP1	ENST00000678807	0.081514065	1.084928477	0.152596318	0.53418107	0.593216265	0.884008552
8__67118248__67137603	CSPP1	ENST00000676847	0.166068038	1.180653428	0.323916393	0.512687969	0.608169607	0.888863271
8__67118248__67137603	CSPP1	ENST00000678834	0.117977669	1.125218984	0.276073938	0.427340842	0.669131089	0.926697859
8__67118248__67137603	CSPP1	ENST00000676317	0.050597515	1.051899434	0.133987009	0.377630003	0.705705473	0.926697859
8__67118248__67137603	CSPP1	ENST00000678821	0.050452045	1.051746425	0.133806421	0.377052492	0.706134596	0.926697859
8__67118248__67137603	CSPP1	ENST00000678645	-0.332061752	0.717443018	1.002773555	-0.331143307	0.740536241	0.926697859
8__67118248__67137603	CSPP1	ENST00000519701	-0.043443218	0.95748692	0.133820635	-0.324637662	0.74545532	0.926697859
8__67118248__67137603	CSPP1	ENST00000677855	0.046006395	1.047081107	0.14667282	0.313666807	0.753774114	0.926697859
8__67118248__67137603	CSPP1	ENST00000678635	0.041714851	1.042597141	0.134728472	0.309621643	0.756848696	0.926697859

8__67118248__67137603	CSPP1	ENST00000678895	-0.084346472	0.919112754	0.309497587	-0.272527074	0.785216777	0.926697859
8__67118248__67137603	CSPP1	ENST00000677276	0.041528135	1.042402489	0.15377752	0.27005335	0.787119211	0.926697859
8__67118248__67137603	CSPP1	ENST00000676697	-0.033408842	0.96714307	0.133738881	-0.249806505	0.802736989	0.926697859
8__67118248__67137603	CSPP1	ENST00000677538	-0.028129701	0.972262255	0.133680618	-0.210424676	0.833336235	0.926697859
8__67118248__67137603	CSPP1	ENST00000677256	-0.026120783	0.974217413	0.133743068	-0.195305697	0.845153637	0.926697859
8__67118248__67137603	CSPP1	ENST00000519668	0.024817749	1.025128273	0.134102898	0.185064974	0.853178074	0.926697859
8__67118248__67137603	CSPP1	ENST00000677473	-0.024114681	0.976173755	0.134122814	-0.179795515	0.857313105	0.926697859
8__67118248__67137603	CSPP1	ENST00000677070	-0.025126852	0.9751862	0.146737314	-0.171236965	0.864037443	0.926697859
8__67118248__67137603	CSPP1	ENST00000679322	-0.022224967	0.978020188	0.133919496	-0.165957668	0.868190264	0.926697859
8__67118248__67137603	CSPP1	ENST00000677009	-0.023009572	0.97725313	0.141692762	-0.162390595	0.870998277	0.926697859
8__67118248__67137603	CSPP1	ENST00000674993	0.071600539	1.074226147	0.452778127	0.158136039	0.874349603	0.926697859
8__67118248__67137603	CSPP1	ENST00000519163	0.020958119	1.021179283	0.133693808	0.156762076	0.875432362	0.926697859
8__67118248__67137603	CSPP1	ENST00000675306	-0.154036783	0.857240489	1.002836605	-0.153601078	0.877924287	0.926697859
8__67118248__67137603	CSPP1	ENST00000677071	-0.016515357	0.983620274	0.133708976	-0.123517188	0.901697565	0.938753629
8__67118248__67137603	CSPP1	ENST00000677455	-0.013406522	0.986682945	0.133732528	-0.100248778	0.920146822	0.945015655
8__67118248__67137603	CSPP1	ENST00000679274	0.011019179	1.011080113	0.140391084	0.078489162	0.937438951	0.949938137
8__67118248__67137603	CSPP1	ENST00000679042	0.005590722	1.005606379	0.133736768	0.041803927	0.966655004	0.966655004

Supplementary Table S9. Statistically significant drugs associated with the patient groups by 11__94583448__94608 and 8__67118248__67137603.

circRNA_ID	DrugName	InhibitorGenes	InhibitorPathways	DrugTypes	pval	diff
11__94583448__94608686	AZD7762	Chk1 inhibitor	B. Kinase inhibitor	Investigational (Ph 1)	0.010302	-3.12798
11__94583448__94608686	Duvelisib	PI3K inhibitor	B. Kinase inhibitor	Investigational (Ph 3)	0.014713	-1.97649
11__94583448__94608686	Romidepsin	HDAC inhibitor	E. Differentiating/ epigenetic modifier	Approved	0.020973	-2.4381
11__94583448__94608686	Bosutinib	Abl, Src inhibitor	B. Kinase inhibitor	Approved	0.022302	-2.16964
11__94583448__94608686	Dabrafenib	B-Raf(V600E) inhibitor	B. Kinase inhibitor	Approved	0.034508	-1.2625
11__94583448__94608686	Decitabine	Nucleoside analog DNA methyl transferase inhibitor	E. Differentiating/ epigenetic modifier	Approved	0.036825	3.404762
11__94583448__94608686	Plicamycin	RNA synthesis inhibitor	A. Conv. Chemo	Approved	0.042927	-3.65685
11__94583448__94608686	Rucaparib	PARP inhibitor	E. Differentiating/ epigenetic modifier	Approved (US)	0.047715	1.183333
8__67118248__67137603	Chloroquine	Antimalaria agent; chemo/radio sensitizer	A. Conv. Chemo	Approved	0.015718	1.58006
8__67118248__67137603	Bleomycin	Glycopeptide antibiotic; causes DNA breaks	A. Conv. Chemo	Approved	0.020614	1.54881
8__67118248__67137603	Sirolimus	binds FKBP12, causes inhibition of mTORC1	C. Rapalog	Approved	0.02484	2.665179
8__67118248__67137603	Nelarabine	Nucleoside analog, DNA, RNA synth inhibitor	A. Conv. Chemo	Approved	0.030347	4.85119
8__67118248__67137603	Temsirolimus	binds FKBP12, causes inhibition of mTORC1	C. Rapalog	Approved	0.031333	2.752679
8__67118248__67137603	Decitabine	Nucleoside analog DNA methyl transferase inhibitor	E. Differentiating/ epigenetic modifier	Approved	0.031653	3.502976
8__67118248__67137603	Everolimus	binds FKBP12, causes inhibition of mTORC1	C. Rapalog	Approved	0.040357	3.371429
8__67118248__67137603	Lenalidomide	Immunomodulatory	D. Immunomodulatory	Approved	0.041286	2.464881

Supplementary Table S10. Drug sensitivity analysis based on high versus low expression of two circRNA host genes PIWIL4 and CSPP1.

hostGene	circID	DrugName	InhibitorGenes	InhibitorPathways	DrugTypes	pval	diff
PIWIL4	11__94583448__94608686	Bleomycin	Glycopeptide antibiotic; causes DNA breaks	A. Conv. Chemo	Approved	0.000770603	1.855760369
PIWIL4	11__94583448__94608686	Rucaparib	PARP inhibitor	E. Differentiating/ epigenetic modifier	Approved (US)	0.000942961	1.590092166
PIWIL4	11__94583448__94608686	Olaparib	PARP inhibitor	E. Differentiating/ epigenetic modifier	Approved	0.003527704	3.037788018
PIWIL4	11__94583448__94608686	Canertinib	pan-HER inhibitor	B. Kinase inhibitor	Investigational (Ph 3)	0.014719526	1.380645161
PIWIL4	11__94583448__94608686	Nelarabine	Nucleoside analog, DNA, RNA synth inhibitor	A. Conv. Chemo	Approved	0.031797098	4.806451613
PIWIL4	11__94583448__94608686	Tofacitinib	JAK3, JAK2(V617F) inhibitor	B. Kinase inhibitor	Approved	0.033180814	2.50921659
PIWIL4	11__94583448__94608686	Cobimetinib	MEK1/2 inhibitor	B. Kinase inhibitor	Approved (US)	0.034736384	-3.291474654
PIWIL4	11__94583448__94608686	Trametinib	MEK1/2 inhibitor	B. Kinase inhibitor	Approved	0.047270952	-3.66359447
CSPP1	8__67118248__67137603	Venetoclax	Bcl-2-selective inhibitor	G. Apoptotic modulator	Approved (US)	0.002765988	7.792813765
CSPP1	8__67118248__67137603	Navitoclax	Bcl-2/Bcl-xL inhibitor	G. Apoptotic modulator	Investigational (Ph 2)	0.007787246	6.301315789
CSPP1	8__67118248__67137603	Canertinib	pan-HER inhibitor	B. Kinase inhibitor	Investigational (Ph 3)	0.010329186	1.37742915
CSPP1	8__67118248__67137603	Cobimetinib	MEK1/2 inhibitor	B. Kinase inhibitor	Approved (US)	0.031536046	-2.936740891
CSPP1	8__67118248__67137603	Cabozantinib	VEGFR2, Met, FLT3, Tie2, Kit and Ret inhibitor	B. Kinase inhibitor	Approved	0.031724322	2.828441296
CSPP1	8__67118248__67137603	Dasatinib	Abl, Src, Kit, EphR... Inhibitor	B. Kinase inhibitor	Approved	0.032909298	-4.041902834
CSPP1	8__67118248__67137603	Nelarabine	Nucleoside analog, DNA, RNA synth inhibitor	A. Conv. Chemo	Approved	0.034465874	4.792510121
CSPP1	8__67118248__67137603	Carfilzomib	Proteasome inhibitor (20S subunit)	L. Protease/proteasome inhibitor	Approved	0.042060746	2.579554656
CSPP1	8__67118248__67137603	Trametinib	MEK1/2 inhibitor	B. Kinase inhibitor	Approved	0.049852178	-3.326417004

Supplementary Table S11. The list of all ELN2022 subgroup-specific circRNAs.

maxgroup	tstat	chisqstat	Adverse	Favorable	Intermediate	tpval	chpval	group	geneSymbol	geneID	tFDR
1	7.61259943	4.4547081	0.04131986	-0.005628212	-0.03569165	3.38E-13	0.069610328	Adverse	CREBBP	ENSG00000005339	4.47E-11
1	7.55740722	0.38178058	0.0155162	-0.006149792	-0.009366404	4.84E-13	0.926696324	Adverse	ZCCHC17	ENSG00000121766	6.10E-11
1	7.52605217	1.45410865	0.01779177	-0.005181422	-0.012610343	5.93E-13	0.455739655	Adverse	PPFIA1	ENSG00000131626	6.85E-11
1	7.33159801	0.26733614	0.01470529	-0.006064391	-0.008640898	2.06E-12	0.789751322	Adverse	CTBP2	ENSG00000175029	1.96E-10
1	7.26444531	2.93148672	0.02860838	-0.005344831	-0.023263548	3.15E-12	0.173734927	Adverse	TMEM181	ENSG00000146433	2.73E-10
1	7.1429982	1.8784302	0.00990283	-0.00287574	-0.007027087	6.74E-12	0.341025345	Adverse	RSRC1	ENSG00000174891	4.92E-10
1	7.13094517	1.15607228	0.05023631	-0.015387822	-0.034848487	7.27E-12	0.564563215	Adverse	UBAC2	ENSG00000134882	5.16E-10
1	6.71697193	2.20489837	0.01029418	-0.002636156	-0.007658029	9.09E-11	0.275145921	Adverse	FOXN3	ENSG00000053254	4.50E-09
1	6.70735068	2.24553599	0.02496236	-0.006275954	-0.018686402	9.62E-11	0.26800094	Adverse	DEF6	ENSG00000023892	4.68E-09
1	6.53132187	0.02751908	0.00778573	-0.004109874	-0.003675852	2.73E-10	0.263510998	Adverse	EFR3A	ENSG00000132294	1.13E-08
1	6.44195969	0.20517876	0.04469439	-0.02631529	-0.018379103	4.59E-10	0.698854443	Adverse	SLC45A4	ENSG00000022567	1.84E-08
1	6.33194505	0.86496882	0.00953922	-0.003110904	-0.006428314	8.65E-10	0.704703857	Adverse	PKN1	ENSG00000123143	3.24E-08
1	6.31352865	2.26626488	0.0112265	-0.002603697	-0.008622805	9.61E-10	0.264436442	Adverse	PRKACB	ENSG00000142875	3.46E-08
1	6.26676794	2.55488492	0.01340447	-0.010718713	-0.002685757	1.25E-09	0.219907641	Adverse	ITGAL	ENSG00000005844	4.35E-08
1	6.25966267	0.76687263	0.02259436	-0.00732749	-0.015266869	1.31E-09	0.762372545	Adverse	FUT8	ENSG00000033170	4.47E-08
1	6.22862962	1.19754302	0.02585163	-0.007162134	-0.018689498	1.56E-09	0.547626608	Adverse	UTRN	ENSG00000152818	5.14E-08
1	6.22862098	3.85144812	0.00774583	-0.001167299	-0.006578531	1.56E-09	0.09940612	Adverse	RAPGEF1	ENSG00000107263	5.14E-08
1	6.2177045	0.01707716	0.00835693	-0.003951872	-0.004405057	1.66E-09	0.207942459	Adverse	MTO1	ENSG00000135297	5.34E-08
1	6.18638887	4.00524054	0.03318737	-0.003752942	-0.029434426	1.98E-09	0.090718049	Adverse	MCTP2	ENSG00000140563	6.30E-08
1	6.17556767	4.26405142	0.0114874	-0.001313545	-0.010173853	2.10E-09	0.077853954	Adverse	VPS13A	ENSG00000197969	6.62E-08
1	6.16207895	3.30517444	0.01191579	-0.001538209	-0.010377579	2.27E-09	0.138124025	Adverse	TRAF5	ENSG00000082512	6.98E-08
1	6.15042776	0.28852277	0.01487657	-0.00896977	-0.005906799	2.42E-09	0.81766254	Adverse	SLC9A7	ENSG00000065923	7.32E-08
1	6.14547147	3.30067865	0.01189437	-0.001592125	-0.010302241	2.49E-09	0.138502533	Adverse	SFMBT2	ENSG00000198879	7.42E-08
1	6.13241224	0.28494395	0.01760247	-0.006865285	-0.010737189	2.68E-09	0.813042124	Adverse	TTC13	ENSG00000143643	7.90E-08
1	6.07519599	0.80154852	0.08068042	-0.025470704	-0.055209721	3.68E-09	0.741261584	Adverse	CORO1C	ENSG00000110880	1.05E-07
1	6.03305671	0.83870267	0.02885447	-0.009110351	-0.019744117	4.65E-09	0.719536142	Adverse	BANP	ENSG00000172530	1.30E-07
1	5.98676707	0.37793317	0.0118409	-0.004391846	-0.00744905	6.01E-09	0.922577106	Adverse	ITGAL	ENSG00000005844	1.62E-07
1	5.95973102	1.96548236	0.01966219	-0.00391273	-0.015749456	6.97E-09	0.321856402	Adverse	TBC1D14	ENSG00000132405	1.84E-07

1	5.84232044	2.31007299	0.06672959	-0.01038713	-0.056342458	1.32E-08	0.257076029	Adverse	RSRC1	ENSG00000174891	3.27E-07
1	5.83409443	0.55719275	0.01201541	-0.003884935	-0.008130471	1.38E-08	0.910787082	Adverse	USP3	ENSG00000140455	3.38E-07
1	5.78431813	1.49043718	0.00737904	-0.001754199	-0.005624841	1.80E-08	0.444297282	Adverse	DDHD1	ENSG00000100523	4.30E-07
1	5.75518292	0.01746668	0.01808394	-0.009506881	-0.008577059	2.11E-08	0.210286999	Adverse	TCEA1	ENSG00000187735	4.95E-07
1	5.73690664	1.8163521	0.0258615	-0.005266525	-0.02059498	2.32E-08	0.355496221	Adverse	HTT	ENSG00000197386	5.41E-07
1	5.73265366	2.77565003	0.10490586	-0.010865542	-0.094040315	2.38E-08	0.191415587	Adverse	CAMSAP1	ENSG00000130559	5.44E-07
1	5.72214167	2.92483823	0.02883926	-0.002767186	-0.026072076	2.51E-08	0.174451934	Adverse	PSME3IP1	ENSG00000172775	5.58E-07
1	5.67376721	3.76831505	0.00872295	-0.000556245	-0.008166706	3.25E-08	0.104464448	Adverse	ERC1	ENSG00000082805	6.92E-07
1	5.65585734	4.08400905	0.02600467	-0.000355828	-0.025648844	3.57E-08	0.086581658	Adverse	ETFA	ENSG00000140374	7.55E-07
1	5.63094826	0.87759783	0.02498659	-0.007450488	-0.017536097	4.07E-08	0.697720917	Adverse	TMEM181	ENSG00000146433	8.37E-07
1	5.63070217	0.02172915	0.0128842	-0.006802021	-0.006082182	4.07E-08	0.234380163	Adverse	SLC15A4	ENSG00000139370	8.37E-07
1	5.60397097	1.34638907	0.01422282	-0.003282642	-0.010940177	4.69E-08	0.491820754	Adverse	HPRT1	ENSG00000165704	9.35E-07
1	5.5574056	2.5305442	0.00736023	-0.001128147	-0.006232088	5.98E-08	0.2233234	Adverse	EPHB4	ENSG00000196411	1.16E-06
1	5.50410366	0.16965683	0.01540242	-0.006147464	-0.009254952	7.88E-08	0.639166052	Adverse	PDE4D	ENSG00000113448	1.46E-06
1	5.49545911	2.1448413	0.00961419	-0.002250753	-0.007363436	8.23E-08	0.286099656	Adverse	ZNF585B	ENSG00000245680	1.51E-06
1	5.49049624	3.85870734	0.04065956	-0.001734018	-0.038925544	8.45E-08	0.098976885	Adverse	PLPBP	ENSG00000147471	1.54E-06
1	5.45294648	3.58463527	0.00728323	-0.00061873	-0.006664499	1.02E-07	0.116632434	Adverse	XPO7	ENSG00000130227	1.81E-06
1	5.44777838	0.81356787	0.02609981	-0.007259897	-0.018839913	1.05E-07	0.734135014	Adverse	MIER1	ENSG00000198160	1.84E-06
1	5.42828395	3.39916696	0.01154499	-0.00071717	-0.010827825	1.16E-07	0.130458707	Adverse	LINC01881	ENSG00000220804	2.01E-06
1	5.41279601	0.09319705	0.04247079	-0.018060014	-0.02441078	1.26E-07	0.479697117	Adverse	ITGAL	ENSG00000005844	2.17E-06
1	5.37691052	4.03176828	0.01607323	0.000396449	-0.016469681	1.51E-07	0.089302235	Adverse	GCN1	ENSG00000089154	2.52E-06
1	5.36714617	0.90104556	0.03595592	-0.009655372	-0.026300544	1.59E-07	0.685003028	Adverse	EAF2	ENSG00000145088	2.62E-06
1	5.36076708	4.63335176	0.01062982	-0.000506459	-0.010123358	1.64E-07	0.0627125	Adverse	MCTP2	ENSG00000140563	2.69E-06
1	5.3399804	0.83809078	0.02883084	-0.007717568	-0.021113267	1.82E-07	0.71988676	Adverse	PHKB	ENSG00000102893	2.95E-06
1	5.33584036	0.529186	0.08435936	-0.026792935	-0.057566422	1.86E-07	0.933898566	Adverse	TBC1D1	ENSG00000065882	2.99E-06
1	5.33374742	0.76107995	0.00706035	-0.002084184	-0.004976163	1.88E-07	0.765981535	Adverse	ACSL3	ENSG00000123983	3.00E-06
1	5.32244803	1.90821761	0.00821455	-0.001550823	-0.006663726	1.99E-07	0.334322768	Adverse	UBP1	ENSG00000153560	3.15E-06
1	5.31375633	0.1651329	0.01559699	-0.006207792	-0.009389195	2.08E-07	0.63105184	Adverse	CTBP1	ENSG00000159692	3.25E-06
1	5.28137311	3.29433471	0.00924147	-0.000501239	-0.008740234	2.44E-07	0.13903853	Adverse	ASH2L	ENSG00000129691	3.74E-06
1	5.27597104	1.50507045	0.0144024	-0.003044838	-0.011357565	2.51E-07	0.439785671	Adverse	CEP72	ENSG00000112877	3.82E-06
1	5.26433734	0.55013095	0.00803058	-0.00250609	-0.005524495	2.66E-07	0.91652839	Adverse	NSUN2	ENSG00000037474	4.03E-06

1	5.24984612	3.34568743	0.01590008	-0.000163839	-0.015736242	2.86E-07	0.134762656	Adverse	R3HDM1	ENSG00000048991	4.28E-06
1	5.24099768	0.0744027	0.01404935	-0.006050249	-0.007999102	2.99E-07	0.429937787	Adverse	CARNMT1	ENSG00000156017	4.45E-06
1	5.230327	2.56223942	0.01935148	-0.001977566	-0.017373914	3.15E-07	0.218886911	Adverse	PICALM	ENSG00000073921	4.67E-06
1	5.22906666	3.93123403	0.0404285	0.000372306	-0.040800806	3.17E-07	0.094794304	Adverse	NAB1	ENSG00000138386	4.67E-06
1	5.19187416	2.55365937	0.01624663	-0.00208498	-0.014161646	3.81E-07	0.220078243	Adverse	ATM	ENSG00000149311	5.47E-06
1	5.15359105	2.43809904	0.01875205	-0.001549018	-0.017203035	4.60E-07	0.236839311	Adverse	PSMA7	ENSG00000101182	6.47E-06
1	5.14787869	1.97272455	0.01548624	-0.002620714	-0.012865526	4.73E-07	0.320317926	Adverse	WWC3	ENSG00000047644	6.62E-06
1	5.14217177	2.83795244	0.01143761	-0.000686621	-0.010750989	4.86E-07	0.184122915	Adverse	PCMTD1	ENSG00000168300	6.78E-06
1	5.13532248	1.62393396	0.01719125	-0.003282005	-0.013909245	5.03E-07	0.405088248	Adverse	RNF138	ENSG00000134758	6.97E-06
1	5.10216944	0.64207865	0.04732424	-0.015183407	-0.032140837	5.91E-07	0.845918175	Adverse	ZFY	ENSG00000067646	8.07E-06
1	5.10144083	0.78263893	0.01027089	-0.002772587	-0.007498302	5.94E-07	0.752670552	Adverse	PLEKHM1P1	ENSG00000214176	8.07E-06
1	5.08394204	2.08211585	0.01471056	-0.001894939	-0.012815622	6.46E-07	0.298066936	Adverse	STK39	ENSG00000198648	8.70E-06
1	5.06500764	0.00695943	0.00714809	-0.003725655	-0.003422436	7.08E-07	0.132969935	Adverse	VCL	ENSG00000035403	9.40E-06
1	5.02590184	0.17788994	0.01038331	-0.006346443	-0.004036872	8.56E-07	0.65361421	Adverse	AMBRA1	ENSG00000110497	1.12E-05
1	5.016723	1.68835492	0.00629888	-0.001187909	-0.005110971	8.94E-07	0.387635938	Adverse	RPS5	ENSG00000083845	1.16E-05
1	4.98324674	1.5799613	0.00752056	-0.001416607	-0.006103955	1.05E-06	0.417532535	Adverse	CRIM1	ENSG00000150938	1.32E-05
1	4.96566021	0.04785922	0.00510835	-0.002267309	-0.002841041	1.14E-06	0.346337665	Adverse	TNPO2	ENSG00000105576	1.39E-05
1	4.96510097	2.77538073	0.01205531	-0.00083766	-0.011217648	1.14E-06	0.191447784	Adverse	HPS4	ENSG00000100099	1.39E-05
1	4.95258761	0.49770701	0.03300761	-0.009988208	-0.023019406	1.21E-06	0.961018759	Adverse	MINDY3	ENSG00000148481	1.46E-05
1	4.91300864	0.58195294	0.01944411	-0.005875959	-0.013568152	1.47E-06	0.891095695	Adverse	DNAJC5	ENSG00000101152	1.73E-05
1	4.90854661	0.34012569	0.00827383	-0.0028194	-0.00545443	1.50E-06	0.880486639	Adverse	AMFR	ENSG00000159461	1.74E-05
1	4.90759799	3.91848913	0.02966361	0.001446523	-0.031110129	1.50E-06	0.095515566	Adverse	CAPZA1	ENSG00000116489	1.74E-05
1	4.90339603	1.91875821	0.00613652	-0.000927875	-0.005208648	1.53E-06	0.331987239	Adverse	SIAE	ENSG00000110013	1.77E-05
1	4.90215496	0.57118899	0.0086202	-0.005972975	-0.002647222	1.54E-06	0.899573657	Adverse	FLI1	ENSG00000151702	1.77E-05
1	4.90060198	1.22126331	0.0171282	-0.01374645	-0.003381754	1.55E-06	0.538225739	Adverse	FKBP5	ENSG00000096060	1.78E-05
1	4.89926661	0.4135932	0.0054972	-0.001895702	-0.003601496	1.56E-06	0.959697303	Adverse	HROB	ENSG00000125319	1.78E-05
1	4.89895548	3.24701452	0.01855535	-7.48E-05	-0.018480502	1.57E-06	0.143107356	Adverse	IKBKB	ENSG00000104365	1.78E-05
1	4.89831703	1.31609805	0.0081646	-0.001557735	-0.006606867	1.57E-06	0.502587061	Adverse	NCOR2	ENSG00000196498	1.78E-05
1	4.88273911	0.0371957	0.1161828	-0.052261991	-0.063920812	1.69E-06	0.30586572	Adverse	RNF13	ENSG00000082996	1.87E-05
1	4.87791479	1.13358093	0.01666982	-0.003645129	-0.013024688	1.73E-06	0.574025614	Adverse	SLC15A4	ENSG00000139370	1.90E-05
1	4.87764168	2.24544818	0.00713863	-0.000955836	-0.006182797	1.73E-06	0.268016153	Adverse	NCOA1	ENSG00000084676	1.90E-05

1	4.82978613	3.19457462	0.00683966	-5.99E-05	-0.006779739	2.17E-06	0.147765978	Adverse	EIF3B	ENSG00000106263	2.35E-05
1	4.82623197	0.63407045	0.00885312	-0.00277198	-0.006081137	2.20E-06	0.851732334	Adverse	SFMBT2	ENSG00000198879	2.38E-05
1	4.82078542	3.06970677	0.00778995	-0.000230552	-0.007559402	2.26E-06	0.159527716	Adverse	RMDN3	ENSG00000137824	2.43E-05
1	4.8131643	2.15489612	0.02643846	-0.002389592	-0.024048864	2.34E-06	0.284232109	Adverse	RNF138	ENSG00000134758	2.50E-05
1	4.80735939	0.07718307	0.00794503	-0.003390161	-0.004554867	2.41E-06	0.437696492	Adverse	FGD6	ENSG00000180263	2.55E-05
1	4.79744871	4.67218334	0.00722079	0.000911964	-0.00813275	2.52E-06	0.061309865	Adverse	KATNAL1	ENSG00000102781	2.66E-05
1	4.7802808	3.13453804	0.01210749	-0.000472863	-0.011634631	2.73E-06	0.153300198	Adverse	ATG7	ENSG00000197548	2.84E-05
1	4.77271492	0.38757144	0.02308371	-0.00748252	-0.01560119	2.82E-06	0.932842546	Adverse	ZCCHC2	ENSG00000141664	2.92E-05
1	4.77080255	0.1054523	0.00694386	-0.002912869	-0.004030987	2.85E-06	0.509235511	Adverse	FOCAD	ENSG00000188352	2.94E-05
1	4.74147663	2.33372318	0.00825802	-0.000649267	-0.007608756	3.26E-06	0.253197513	Adverse	SIRT5	ENSG00000124523	3.34E-05
1	4.70945101	0.03239498	0.00814857	-0.004505539	-0.003643029	3.78E-06	0.285672949	Adverse	METTL9	ENSG00000197006	3.75E-05
1	4.67810332	0.09937202	0.00706111	-0.004162066	-0.002899044	4.36E-06	0.494830922	Adverse	CPT1A	ENSG00000110090	4.25E-05
1	4.67804198	2.34549279	0.00854693	-0.000559096	-0.007987833	4.36E-06	0.251291645	Adverse	SHOC2	ENSG00000108061	4.25E-05
1	4.67590952	0.25684374	0.00673757	-0.004222171	-0.002515397	4.40E-06	0.775406233	Adverse	TENT4A	ENSG00000112941	4.27E-05
1	4.66357382	1.92274788	0.06797764	-0.007696253	-0.060281387	4.65E-06	0.331108104	Adverse	USP25	ENSG00000155313	4.48E-05
1	4.6580245	0.5339052	0.01166896	-0.003441405	-0.008227555	4.77E-06	0.929939285	Adverse	CCSER2	ENSG00000107771	4.57E-05
1	4.64625126	1.48635407	0.01403498	-0.002133712	-0.01190127	5.03E-06	0.445566018	Adverse	TMEM181	ENSG00000146433	4.75E-05
1	4.6431387	1.10774385	0.02153513	-0.004259008	-0.017276126	5.11E-06	0.585145822	Adverse	MINDY3	ENSG00000148481	4.80E-05
1	4.61470688	3.95459106	0.02109705	0.001557337	-0.022654383	5.81E-06	0.093487349	Adverse	PICALM	ENSG00000073921	5.38E-05
1	4.59535846	1.34345907	0.01147374	-0.001769641	-0.009704098	6.33E-06	0.492849747	Adverse	DOCK11	ENSG00000147251	5.75E-05
1	4.57314455	0.06835703	0.0079277	-0.003377579	-0.00455012	6.99E-06	0.412511721	Adverse	EIF3E	ENSG00000104408	6.27E-05
1	4.52038829	3.12451845	0.02483956	0.000386351	-0.025225913	8.84E-06	0.15424531	Adverse	NNT	ENSG00000112992	7.66E-05
1	4.51264088	0.35583511	0.00954339	-0.003037539	-0.00650585	9.15E-06	0.898343087	Adverse	PRKDC	ENSG00000253729	7.88E-05
1	4.5095037	2.32627825	0.01227414	-0.000575791	-0.011698349	9.28E-06	0.254411377	Adverse	RNF149	ENSG00000163162	7.96E-05
1	4.50289004	3.55005357	0.01317086	0.001123734	-0.014294596	9.55E-06	0.119087055	Adverse	EHBP1	ENSG00000115504	8.12E-05
1	4.49673453	0.04773163	0.01054363	-0.005931669	-0.004611964	9.82E-06	0.345883015	Adverse	ITGAM	ENSG00000169896	8.30E-05
1	4.48842773	0.10092324	0.017979	-0.007359266	-0.010619739	1.02E-05	0.498550985	Adverse	UBQLN1	ENSG00000135018	8.55E-05
1	4.48721013	2.85755202	0.00922565	1.63E-05	-0.009241954	1.02E-05	0.181891616	Adverse	FLVCR1	ENSG00000162769	8.57E-05
1	4.47797111	0.39050924	0.10790292	-0.074098223	-0.033804693	1.07E-05	0.935936307	Adverse	SLC8A1	ENSG00000183023	8.88E-05
1	4.4735657	1.01693605	0.01637595	-0.003100655	-0.013275293	1.09E-05	0.62649378	Adverse	USP3	ENSG00000140455	8.92E-05
1	4.46435616	0.00117445	0.00603968	-0.002960007	-0.003079673	1.13E-05	0.054676652	Adverse	LARP4B	ENSG00000107929	9.20E-05

1	4.44247642	3.34378665	0.01724388	0.000895412	-0.018139289	1.25E-05	0.134918393	Adverse	DOCK11	ENSG00000147251	0.000100659
1	4.43514595	1.44462499	0.00655283	-0.00090676	-0.005646065	1.29E-05	0.458784762	Adverse	STK10	ENSG00000072786	0.000103029
1	4.43426529	1.76757119	0.01479696	-0.001438326	-0.013358631	1.29E-05	0.367365899	Adverse	ELP3	ENSG00000134014	0.000103128
1	4.42705089	0.00121076	0.02577658	-0.012607403	-0.013169177	1.33E-05	0.055515078	Adverse	TBC1D14	ENSG00000132405	0.000106114
1	4.41641644	2.46260067	0.00754344	-0.000165985	-0.007377454	1.40E-05	0.233171216	Adverse	SF3B3	ENSG00000189091	0.000109875
1	4.41553477	0.97407569	0.01467043	-0.002792019	-0.011878409	1.40E-05	0.647331625	Adverse	PCNT	ENSG00000160299	0.000109984
1	4.40465048	0.23579592	0.00596146	-0.002149834	-0.00381163	1.47E-05	0.745481703	Adverse	FUT8	ENSG00000033170	0.000114654
1	4.39845583	2.55577912	0.01074877	-0.001013918	-0.009734848	1.51E-05	0.219783256	Adverse	SUCO	ENSG00000094975	0.000117443
1	4.38775177	1.07299712	0.00569995	-0.001103172	-0.00459678	1.58E-05	0.600538896	Adverse	LRRK1	ENSG00000154237	0.000122323
1	4.35983425	0.00899744	0.00424184	-0.002005779	-0.002236056	1.78E-05	0.151139726	Adverse	TRAPPC6B	ENSG00000182400	0.000137203
1	4.35666823	0.01151789	0.00614226	-0.00326811	-0.002874151	1.81E-05	0.170932073	Adverse	TRAF3	ENSG00000131323	0.000138315
1	4.33287884	0.00406213	0.00560713	-0.002700368	-0.002906757	2.00E-05	0.101637286	Adverse	WDR37	ENSG00000047056	0.000150393
1	4.3326806	2.94776054	0.01924234	0.00101673	-0.02025907	2.00E-05	0.171993301	Adverse	GAPVD1	ENSG00000165219	0.000150393
1	4.32398299	3.69955617	0.01520813	0.001393679	-0.016601807	2.08E-05	0.108853887	Adverse	YY1AP1	ENSG00000163374	0.000155249
1	4.31937927	0.25941329	0.01302678	-0.008403151	-0.004623626	2.12E-05	0.778952957	Adverse	ZNF81	ENSG00000197779	0.000155813
1	4.31702648	2.64357589	0.02298672	-0.000121246	-0.022865471	2.14E-05	0.207938578	Adverse	SATB1	ENSG00000182568	0.000156969
1	4.30938967	0.84129728	0.00626015	-0.001418434	-0.004841714	2.21E-05	0.718052024	Adverse	ZMYND8	ENSG00000101040	0.000160887
1	4.30557423	1.79558625	0.01274835	-0.000967845	-0.011780508	2.25E-05	0.360494027	Adverse	IKZF1	ENSG00000185811	0.000162669
1	4.30350789	0.15961065	0.00472927	-0.001777645	-0.002951621	2.27E-05	0.620969524	Adverse	KDM5A	ENSG00000073614	0.000163679
1	4.29371034	1.97671824	0.00557274	-0.000460057	-0.005112685	2.36E-05	0.319473122	Adverse	EZH2	ENSG00000106462	0.000168883
1	4.28938613	1.52474576	0.01093209	-0.001350141	-0.009581945	2.41E-05	0.433805458	Adverse	DDI2	ENSG00000197312	0.000171131
1	4.28543086	1.86789646	0.0075133	-0.000497165	-0.007016134	2.45E-05	0.343432391	Adverse	UBP1	ENSG00000153560	0.00017314
1	4.28412219	3.81377101	0.01335154	0.001833331	-0.015184872	2.46E-05	0.101665768	Adverse	FOXK2	ENSG00000141568	0.00017366
1	4.27612349	4.6549669	0.03006877	0.004416683	-0.034485453	2.55E-05	0.061927659	Adverse	ERC1	ENSG00000082805	0.000177838
1	4.24734919	0.74102976	0.00611233	-0.00148846	-0.004623874	2.88E-05	0.778662417	Adverse	PIP4K2A	ENSG00000150867	0.000197336
1	4.24110912	0.05880672	0.01824378	-0.007699604	-0.010544172	2.95E-05	0.383215973	Adverse	TMED2	ENSG00000086598	0.000200668
1	4.22612352	3.13227476	0.00510343	0.000219818	-0.005323248	3.14E-05	0.153513139	Adverse	TAB2	ENSG00000055208	0.000212077
1	4.22066963	0.00948529	0.00804681	-0.0042712	-0.003775608	3.22E-05	0.155170523	Adverse	EAF2	ENSG00000145088	0.000215409
1	4.21277752	3.69732381	0.03361079	0.003419892	-0.037030687	3.33E-05	0.108999631	Adverse	PICALM	ENSG00000073921	0.000221577
1	4.20866033	0.41106855	0.00486416	-0.001499659	-0.003364499	3.38E-05	0.957144706	Adverse	ATF2	ENSG00000115966	0.000224885
1	4.20071358	2.1356744	0.00636216	-0.00035275	-0.006009412	3.50E-05	0.287814322	Adverse	EDC3	ENSG00000179151	0.000231367

1	4.17943841	0.01079293	0.00635722	-0.002969497	-0.003387721	3.82E-05	0.165485138	Adverse	XPO6	ENSG00000169180	0.000251013
1	4.17869853	1.29099598	0.01464684	-0.002115906	-0.012530935	3.83E-05	0.51172897	Adverse	SPIDR	ENSG00000164808	0.000251191
1	4.17184877	0.00600637	0.00665886	-0.003177617	-0.003481243	3.94E-05	0.1235497	Adverse	VPS13A	ENSG00000197969	0.000257225
1	4.16503649	2.38041236	0.00791862	-0.000381847	-0.007536775	4.06E-05	0.245730223	Adverse	PCMT1	ENSG00000120265	0.000262745
1	4.14322609	0.09856804	0.00718592	-0.004256359	-0.002929563	4.44E-05	0.492890291	Adverse	UBASH3B	ENSG00000154127	0.000282281
1	4.14118406	0.36806781	0.00845819	-0.002427792	-0.006030398	4.48E-05	0.911881269	Adverse	RBL1	ENSG00000080839	0.000283371
1	4.1355275	0.98630701	0.00659499	-0.005457168	-0.001137822	4.58E-05	0.641293301	Adverse	ZSWIM6	ENSG00000130449	0.000288087
1	4.12437211	0.84382313	0.00715184	-0.001543709	-0.005608126	4.80E-05	0.716611276	Adverse	NR2C2	ENSG00000177463	0.000300948
1	4.09196276	4.31585282	0.00957205	0.002072924	-0.011644975	5.48E-05	0.07551768	Adverse	METTL6	ENSG00000206562	0.000338366
1	4.09088959	1.13872936	0.00728452	-0.001032258	-0.006252265	5.50E-05	0.57184196	Adverse	VMP1	ENSG00000062716	0.000339098
1	4.06800209	2.90475489	0.00666783	0.000251374	-0.006919204	6.04E-05	0.176637357	Adverse	UMPS	ENSG00000114491	0.000366568
1	4.06727269	2.19755993	0.0080104	0.000133321	-0.008143725	6.06E-05	0.276458789	Adverse	TRA2B	ENSG00000136527	0.000366854
1	4.05787672	0.00464641	0.00858707	-0.004495783	-0.004091288	6.30E-05	0.108690723	Adverse	API5	ENSG00000166181	0.000377835
1	4.05360961	0.12564451	0.01234518	-0.004626019	-0.007719163	6.41E-05	0.554017181	Adverse	APBB1IP	ENSG00000077420	0.000381716
1	4.0532342	2.87616426	0.01309938	0.001174747	-0.014274126	6.42E-05	0.179799873	Adverse	UBXN4	ENSG00000144224	0.000381716
1	4.03377189	0.8925875	0.05077827	-0.010091228	-0.040687047	6.94E-05	0.689554163	Adverse	PTP4A2	ENSG00000184007	0.000407762
1	4.0295761	0.2715785	0.02026982	-0.006633671	-0.013636144	7.06E-05	0.795450271	Adverse	SAFB2	ENSG00000130254	0.000413855
1	4.02524553	4.17370681	0.01429917	0.002993067	-0.017292239	7.19E-05	0.082111766	Adverse	MAPKAP1	ENSG00000119487	0.000419379
1	4.02211335	0.40503927	0.00579874	-0.001689914	-0.004108824	7.28E-05	0.951003644	Adverse	MTDH	ENSG00000147649	0.000423818
1	4.00386723	2.02355858	0.01417921	0.000138048	-0.014317256	7.83E-05	0.309751631	Adverse	CCSER2	ENSG00000107771	0.000452308
1	3.99931331	0.17490065	0.00953182	-0.003450166	-0.006081656	7.98E-05	0.648414666	Adverse	ATP6V0A1	ENSG00000033627	0.000457002
1	3.99623802	2.87029715	0.03195514	0.002979427	-0.034934564	8.08E-05	0.180456419	Adverse	VAMP3	ENSG00000049245	0.000461566
1	3.96435405	2.6439071	0.01561774	0.001064762	-0.016682502	9.17E-05	0.207895243	Adverse	RCAN3	ENSG00000117602	0.000512689
1	3.96069687	2.00814229	0.01353956	-0.000675613	-0.012863946	9.31E-05	0.312913599	Adverse	BBX	ENSG00000114439	0.000516307
1	3.9456411	3.79152345	0.0165457	0.003128433	-0.019674135	9.88E-05	0.103025486	Adverse	OMA1	ENSG00000162600	0.000537144
1	3.93738996	3.40903627	0.00878779	0.001517047	-0.010304837	0.000102113	0.129680608	Adverse	NAA25	ENSG00000111300	0.000550696
1	3.93394937	0.02870754	0.0053712	-0.002375523	-0.002995675	0.000103515	0.269087853	Adverse	TGFB1	ENSG00000105329	0.000556134
1	3.93346728	1.54292616	0.01006246	-0.000778492	-0.009283965	0.000103712	0.428365553	Adverse	CNST	ENSG00000162852	0.000556134
1	3.93004591	2.03434628	0.00674013	-7.95E-05	-0.006660667	0.000105127	0.307560577	Adverse	ZFYVE26	ENSG00000072121	0.00056257
1	3.92085482	0.37133292	0.00457552	-0.001348368	-0.00322715	0.000109017	0.915442781	Adverse	PAPOLA	ENSG00000090060	0.000577988
1	3.92011567	0.28212884	0.01809946	-0.005603344	-0.012496111	0.000109336	0.809381437	Adverse	DYNC1H1	ENSG00000197102	0.000578242

1	3.91916077	3.38914614	0.01037434	0.001192185	-0.011566527	0.000109749	0.131253853	Adverse	CEPT1	ENSG00000134255	0.000578372
1	3.91451544	4.08336028	0.00556556	0.000847681	-0.006413244	0.00011178	0.086614904	Adverse	HERC1	ENSG00000103657	0.000585484
1	3.91412291	2.91304794	0.01615524	0.001640699	-0.017795937	0.000111953	0.175731353	Adverse	DCP2	ENSG00000172795	0.000585484
1	3.91366687	2.01602954	0.00894083	-0.000342815	-0.00859802	0.000112154	0.311291325	Adverse	PRKCB	ENSG00000166501	0.000585484
1	3.90479207	0.51689821	0.00481968	-0.003571556	-0.001248125	0.000116146	0.944335649	Adverse	UBP1	ENSG00000153560	0.000602045
1	3.87258846	0.35850444	0.00704117	-0.002093069	-0.004948104	0.00013179	0.90132399	Adverse	MYH9	ENSG00000100345	0.000673611
1	3.87227448	3.79593544	0.01688434	0.002387993	-0.019272335	0.000131952	0.102754315	Adverse	DGKD	ENSG00000077044	0.000673611
1	3.8708319	1.39946345	0.01104681	-0.001163785	-0.009883023	0.000132698	0.473626854	Adverse	LCLAT1	ENSG00000172954	0.000676176
1	3.86816822	0.0014351	0.0052234	-0.002677078	-0.002546323	0.000134087	0.06043759	Adverse	MTO1	ENSG00000135297	0.000680749
1	3.85939087	1.13713498	0.00897529	-0.001215056	-0.007760229	0.000138761	0.57251707	Adverse	ZEB1	ENSG00000148516	0.000700629
1	3.85070834	1.80056679	0.01530993	-6.76E-05	-0.015242351	0.000143536	0.359287975	Adverse	FBXL5	ENSG00000118564	0.000719496
1	3.84466545	0.6575885	0.01079661	-0.002458053	-0.008338554	0.00014695	0.834825176	Adverse	ZBTB10	ENSG00000205189	0.000731322
1	3.84377203	0.12207625	0.00562579	-0.003459805	-0.002165984	0.000147462	0.546413103	Adverse	FLI1	ENSG00000151702	0.000732551
1	3.84069666	4.15114063	0.03707676	0.009196369	-0.046273129	0.000149234	0.083212976	Adverse	MORC3	ENSG00000159256	0.000740032
1	3.83156867	1.19594803	0.0090588	-0.001073265	-0.00798553	0.000154616	0.548266086	Adverse	RNF13	ENSG00000082996	0.00076278
1	3.82559778	1.00685534	0.01162148	-0.001391223	-0.01023026	0.000158235	0.631314765	Adverse	ATXN1	ENSG00000124788	0.000779091
1	3.82269712	3.16558495	0.01427248	0.002158027	-0.016430505	0.000160022	0.150410981	Adverse	GALK2	ENSG00000156958	0.000786493
1	3.82071482	1.6387708	0.00794584	-0.000574931	-0.00737091	0.000161254	0.400988405	Adverse	BAZ1A	ENSG00000198604	0.000791146
1	3.81621831	1.02271108	0.03911844	-0.006765128	-0.032353312	0.000164083	0.623753593	Adverse	TXLNGY	ENSG00000131002	0.000802182
1	3.79674875	4.97008338	0.01897667	0.005173396	-0.024150061	0.000176879	0.051578808	Adverse	UPF2	ENSG00000151461	0.000855452
1	3.79558783	2.62938552	0.00793379	0.000806015	-0.008739809	0.000177671	0.20980461	Adverse	VIRMA	ENSG00000164944	0.000855452
1	3.79546305	3.65298748	0.0098483	0.001884177	-0.011732472	0.000177756	0.111937468	Adverse	MTR	ENSG00000116984	0.000855452
1	3.79441823	4.51429595	0.00655981	0.00188303	-0.008442835	0.000178472	0.067225434	Adverse	MTDH	ENSG00000147649	0.000857409
1	3.78840074	1.42573347	0.01228962	-0.001008648	-0.011280968	0.00018265	0.464924006	Adverse	MGAT5	ENSG00000152127	0.000875959
1	3.78188704	1.25235743	0.0247766	-0.002490906	-0.022285692	0.000187275	0.526205395	Adverse	CCDC91	ENSG00000123106	0.000896592
1	3.77904956	0.29487602	0.01641838	-0.00503989	-0.011378487	0.000189325	0.82577465	Adverse	UXS1	ENSG00000115652	0.000901732
1	3.77260515	1.08842485	0.00871001	-0.001366149	-0.007343859	0.000194058	0.593640948	Adverse	IGF1R	ENSG00000140443	0.000921112
1	3.76615915	4.46022831	0.01288601	0.002734147	-0.01562016	0.000198905	0.06938572	Adverse	MAPK1	ENSG00000100030	0.000940894
1	3.7593063	4.27613225	0.03289598	0.009156346	-0.042052328	0.000204182	0.07730241	Adverse	MCTP2	ENSG00000140563	0.000962574
1	3.75711643	0.09121506	0.0099729	-0.006159531	-0.003813366	0.000205896	0.474723797	Adverse	NFX1	ENSG00000086102	0.000966885
1	3.73917301	1.5407185	0.00698576	-0.000412997	-0.006572763	0.000220462	0.429021778	Adverse	RNF13	ENSG00000082996	0.001025345

1	3.73885553	0.0820128	0.01883747	-0.011402049	-0.007435416	0.000220729	0.45082394	Adverse	MYO9B	ENSG00000099331	0.001025345
1	3.73592056	2.53230967	0.00757376	0.000381497	-0.007955254	0.000223205	0.223073697	Adverse	WDR37	ENSG00000047056	0.001034654
1	3.72992371	1.59393402	0.00552364	-0.000470469	-0.005053173	0.000228345	0.413529944	Adverse	TNK2	ENSG00000061938	0.001055424
1	3.72980654	0.90417047	0.00616978	-0.000982528	-0.005187252	0.000228447	0.683331823	Adverse	PHC3	ENSG00000173889	0.001055424
1	3.72313818	1.37250662	0.0060855	-0.000524029	-0.005561475	0.000234297	0.482763357	Adverse	PRKCB	ENSG00000166501	0.001078854
1	3.72199301	2.25359957	0.01313975	0.000489981	-0.013629733	0.000235315	0.266608002	Adverse	FOCAD	ENSG00000188352	0.001081748
1	3.69934662	4.18144669	0.02138929	0.005542087	-0.026931378	0.000256339	0.081737601	Adverse	UBAP2	ENSG00000137073	0.001164873
1	3.65860655	3.51147732	0.00650792	0.0011785	-0.007686423	0.000298685	0.121890177	Adverse	ATAD1	ENSG00000138138	0.001329573
1	3.64787638	0.02951041	0.00809098	-0.00456541	-0.00352557	0.000310888	0.272788376	Adverse	UBR1	ENSG00000159459	0.00137445
1	3.63990372	0.0103885	0.00649248	-0.003495364	-0.002997116	0.000320256	0.162366008	Adverse	SBDS	ENSG00000126524	0.001409127
1	3.63877327	3.75822637	0.01813254	0.003875654	-0.02200819	0.000321606	0.105096581	Adverse	CNOT6L	ENSG00000138767	0.001412823
1	3.63453072	2.75935164	0.01232465	0.0014971	-0.013821753	0.000326719	0.19337483	Adverse	FAM216A	ENSG00000204856	0.001430751
1	3.6333343	0.05821975	0.00609051	-0.002504527	-0.003585982	0.000328175	0.381335711	Adverse	VWA8	ENSG00000102763	0.001434772
1	3.62984448	4.6570287	0.01217317	0.002751884	-0.014925056	0.000332456	0.061853333	Adverse	TFRC	ENSG00000072274	0.001446167
1	3.62923875	4.4620094	0.00983478	0.003141521	-0.012976299	0.000333204	0.069313413	Adverse	HECTD1	ENSG00000092148	0.001446167
1	3.6224329	0.24969374	0.01026439	-0.003216566	-0.007047822	0.000341723	0.765418384	Adverse	TTBK2	ENSG00000128881	0.001475476
1	3.61752393	0.05889028	0.01256923	-0.005157083	-0.007412149	0.000347994	0.383482843	Adverse	PHKB	ENSG00000102893	0.001495563
1	3.61029917	0.08029665	0.00479707	-0.001926811	-0.002870256	0.00035742	0.446208385	Adverse	SNX6	ENSG00000129515	0.001533696
1	3.60905834	0.40586195	0.00665074	-0.00174106	-0.004909681	0.000359063	0.951845352	Adverse	TIMMDC1	ENSG00000113845	0.001534121
1	3.59767631	0.07652622	0.00481696	-0.002891149	-0.001925811	0.000374467	0.435877261	Adverse	SPI1	ENSG00000066336	0.001584767
1	3.58827564	0.02972593	0.00549	-0.002395291	-0.003094706	0.000387656	0.273772852	Adverse	TOLLIP	ENSG00000078902	0.001628154
1	3.57553649	2.9275416	0.00639279	0.000969929	-0.007362721	0.000406225	0.174160003	Adverse	ETFA	ENSG00000140374	0.001698427
1	3.56130109	1.51058559	0.00415838	-0.000158074	-0.004000304	0.000427963	0.438099505	Adverse	UBAP2L	ENSG00000143569	0.001762997
1	3.56110121	0.07794233	0.0034742	-0.00138628	-0.002087923	0.000428276	0.439788972	Adverse	SNX27	ENSG00000143376	0.001762997
1	3.56108357	4.18106931	0.0061234	0.001759852	-0.007883252	0.000428303	0.081755803	Adverse	GNB1	ENSG00000078369	0.001762997
1	3.56085247	1.66003665	0.01679485	-0.000121034	-0.016673814	0.000428665	0.395196752	Adverse	SMAD2	ENSG00000175387	0.001762997
1	3.55770059	0.02476238	0.00522354	-0.002915519	-0.002308024	0.000433631	0.250078789	Adverse	DLG1	ENSG00000075711	0.001778588
1	3.5457176	0.54970555	0.01781359	-0.003578957	-0.014234631	0.000453008	0.916876064	Adverse	KIF20B	ENSG00000138182	0.001846672
1	3.54420522	4.41078612	0.01057233	0.003709532	-0.014281859	0.00045551	0.07142476	Adverse	UBAP2	ENSG00000137073	0.001847489
1	3.54390963	0.21661477	0.01458713	-0.004644379	-0.009942752	0.000456001	0.716737018	Adverse	DENND1A	ENSG00000119522	0.001847489
1	3.5392992	0.36431782	0.01476126	-0.003751883	-0.011009375	0.000463717	0.907764094	Adverse	PPP6R2	ENSG00000100239	0.001862933

1	3.53456777	0.02799529	0.00534977	-0.002335849	-0.003013917	0.000471763	0.265760185	Adverse	FAM120A	ENSG00000048828	0.001881623
1	3.53205897	0.30348924	0.00894998	-0.002640339	-0.006309641	0.000476083	0.836593593	Adverse	TRIM35	ENSG00000104228	0.001896123
1	3.52630412	1.89460935	0.00781451	0.000506044	-0.008320554	0.000486131	0.337365895	Adverse	NUP98	ENSG00000110713	0.001925906
1	3.52397949	2.00754807	0.00725826	0.000109187	-0.007367451	0.000490246	0.313036208	Adverse	ARHGAP26	ENSG00000145819	0.001938606
1	3.51551624	0.53639673	0.00605021	-0.00142028	-0.004629925	0.000505506	0.927859776	Adverse	ENSG0000011209	ENSG00000112096	0.00199326
1	3.514902	0.30779276	0.0071646	-0.002068993	-0.005095604	0.00050663	0.841924389	Adverse	GLG1	ENSG00000090863	0.001994857
1	3.48227878	2.23028779	0.00491088	0.000254705	-0.005165588	0.000569843	0.270657278	Adverse	MTMR1	ENSG00000063601	0.002203075
1	3.47707078	1.94595795	0.04085882	0.000853493	-0.041712314	0.000580593	0.326046174	Adverse	ELK4	ENSG00000158711	0.002229091
1	3.46883766	2.212645	0.01481021	0.001490592	-0.016300798	0.000597975	0.273767602	Adverse	CCDC7	ENSG00000216937	0.002282629
1	3.46490814	4.94394167	0.00457078	0.001513014	-0.00608379	0.000606442	0.052364527	Adverse	BBX	ENSG00000114439	0.002309143
1	3.45225427	0.70789944	0.00452959	-0.000832172	-0.003697419	0.000634474	0.800284057	Adverse	PREX1	ENSG00000124126	0.002391549
1	3.45202812	0.75099934	0.01608009	-0.002270793	-0.013809297	0.000634986	0.772320039	Adverse	RNF19B	ENSG00000116514	0.002391549
1	3.4454713	2.49979217	0.01488136	0.002249146	-0.017130505	0.000649994	0.227722645	Adverse	PICALM	ENSG00000073921	0.002434843
1	3.44313864	0.36728133	0.0123969	-0.00304789	-0.009349005	0.000655413	0.911020165	Adverse	NSD2	ENSG00000109685	0.002448525
1	3.43954553	0.40406438	0.00756654	-0.001901711	-0.005664827	0.000663843	0.950004658	Adverse	NCOA1	ENSG00000084676	0.002470031
1	3.42456101	0.16200231	0.00321069	-0.002087514	-0.001123176	0.000700102	0.625360621	Adverse	HSDL2	ENSG00000119471	0.002577269
1	3.42401696	1.59912538	0.01910938	-0.000712475	-0.018396904	0.000701453	0.412054401	Adverse	KAT6A	ENSG00000083168	0.002578816
1	3.42245103	0.03074068	0.00630885	-0.002725534	-0.003583315	0.000705354	0.278359637	Adverse	TCF25	ENSG00000141002	0.002589724
1	3.42064392	1.89519986	0.00625441	0.000378174	-0.006632587	0.000709881	0.337233186	Adverse	STK39	ENSG00000198648	0.002602898
1	3.41988487	0.11256332	0.00598327	-0.002216542	-0.003766727	0.000711791	0.525511004	Adverse	AZIN1	ENSG00000155096	0.002606452
1	3.41859054	0.08261216	0.00680199	-0.004220563	-0.002581427	0.000715058	0.452423581	Adverse	PCCA	ENSG00000175198	0.002614962
1	3.41738698	1.61666281	0.00461458	-0.000124275	-0.004490305	0.000718109	0.40711548	Adverse	IQGAP2	ENSG00000145703	0.002622659
1	3.40041178	0.01600605	0.00446576	-0.002003177	-0.002462579	0.000762453	0.201351509	Adverse	LYN	ENSG00000254087	0.002762771
1	3.39766839	4.0555088	0.00579509	0.001603165	-0.00739826	0.000769855	0.088054899	Adverse	MCTP2	ENSG00000140563	0.00278595
1	3.3859647	3.93726362	0.00637046	0.001948952	-0.008319413	0.000802192	0.09445508	Adverse	CAMSAP1	ENSG00000130559	0.002878404
1	3.37274961	2.14148154	0.04633772	0.003711077	-0.050048801	0.000840226	0.286726761	Adverse	FAM120A	ENSG00000048828	0.003001425
1	3.36030697	0.99483784	0.00650057	-0.000649495	-0.005851076	0.000877566	0.637125662	Adverse	RASA1	ENSG00000145715	0.00311076
1	3.35286105	2.40936377	0.00418041	0.000414834	-0.004595247	0.000900645	0.241222753	Adverse	DCAF17	ENSG00000115827	0.003184425
1	3.35094703	2.61561502	0.01165756	0.002435922	-0.014093484	0.000906669	0.211632984	Adverse	KDM4B	ENSG00000127663	0.003201638
1	3.34661863	4.06578199	0.00794345	0.002368651	-0.010312104	0.000920428	0.087520837	Adverse	INTS2	ENSG00000108506	0.003233748
1	3.34541293	3.29157608	0.01396712	0.003780895	-0.017748017	0.000924296	0.139272298	Adverse	R3HDM1	ENSG00000048991	0.003243225

1	3.34343029	3.45132513	0.00441575	0.000752432	-0.005168186	0.000930688	0.126402162	Adverse	DEK	ENSG00000124795	0.003261386
1	3.34307911	1.21544327	0.00429216	-0.000253412	-0.004038753	0.000931825	0.540513538	Adverse	ILKAP	ENSG00000132323	0.003261386
1	3.34202949	0.07425218	0.00445422	-0.001730688	-0.002723535	0.000935229	0.429513348	Adverse	ADAM10	ENSG00000137845	0.003268755
1	3.3393132	0.43394291	0.00439869	-0.001026885	-0.003371803	0.000944094	0.979878922	Adverse	USP3	ENSG00000140455	0.003287724
1	3.33003648	3.0532748	0.00437296	-0.00527889	0.000905933	0.000974962	0.161149046	Adverse	EPB41L2	ENSG00000079819	0.003378864
1	3.31638305	1.62815071	0.00418603	-5.50E-06	-0.004180533	0.001022104	0.403918046	Adverse	CEP192	ENSG00000101639	0.003528361
1	3.31566837	0.0063362	0.00378399	-0.00201333	-0.001770656	0.00102463	0.126889586	Adverse	PCMTD1	ENSG00000168300	0.003532062
1	3.31002162	0.95668547	0.00523904	-0.000505748	-0.004733295	0.001044786	0.656046775	Adverse	DNTTIP2	ENSG00000067334	0.003575489
1	3.30965666	1.27834927	0.00761639	-0.000447761	-0.007168625	0.001046101	0.516412372	Adverse	CPT1A	ENSG00000110090	0.003575576
1	3.30561211	0.71596038	0.00550858	-0.00081656	-0.004692025	0.001060781	0.794944368	Adverse	UPF2	ENSG00000151461	0.003599126
1	3.30515013	0.57295248	0.01246273	-0.01016735	-0.002295378	0.00106247	0.898176118	Adverse	SLC8A1	ENSG00000183023	0.003600219
1	3.3048139	2.59237883	0.00503078	-0.005790003	0.000759227	0.001063701	0.214757908	Adverse	SEL1L3	ENSG00000091490	0.003600219
1	3.30008507	4.05165379	0.00761874	0.002394312	-0.010013049	0.001081152	0.088256188	Adverse	UBE3D	ENSG00000118420	0.003650371
1	3.28886938	3.25503376	0.01085944	0.003070056	-0.013929499	0.001123612	0.142408942	Adverse	FGGY	ENSG00000172456	0.003775337
1	3.28841409	4.5659894	0.01145003	0.004392638	-0.015842669	0.001125368	0.065225662	Adverse	SHOC2	ENSG00000108061	0.003776659
1	3.27229691	0.96786453	0.0041499	-0.000591546	-0.003558353	0.001189194	0.650426676	Adverse	SLC6A6	ENSG00000131389	0.003966843
1	3.26554815	2.19106872	0.00731792	0.000901924	-0.008219849	0.001216906	0.27762594	Adverse	UTRN	ENSG00000152818	0.004048787
1	3.26197891	1.92489016	0.0209092	0.002359563	-0.023268762	0.001231803	0.330637147	Adverse	HERC1	ENSG00000103657	0.004079519
1	3.25971971	1.4145323	0.01042409	0.000470114	-0.0108942	0.001241319	0.468610921	Adverse	PRMT5	ENSG00000100462	0.00410613
1	3.25730045	0.44440836	0.00500548	-0.001105256	-0.003900222	0.001251586	0.989995266	Adverse	BTAF1	ENSG00000095564	0.004126417
1	3.25480964	1.67268424	0.00631309	0.0001863	-0.00649939	0.001262238	0.391798776	Adverse	CEP350	ENSG00000135837	0.00415369
1	3.25301629	1.69682432	0.00423182	7.65E-05	-0.004308271	0.001269959	0.385407623	Adverse	S100PBP	ENSG00000116497	0.004171004
1	3.23811968	2.36741258	0.00852816	0.001447316	-0.009975473	0.001335813	0.24778449	Adverse	BRIP1	ENSG00000136492	0.004341001
1	3.22993407	3.50842105	0.005958	0.001580587	-0.007538584	0.001373334	0.12211524	Adverse	NMRAL1	ENSG00000153406	0.004435123
1	3.22853648	4.2627745	0.00492139	0.001630922	-0.006552317	0.001379837	0.077912492	Adverse	LRCH3	ENSG00000186001	0.004442401
1	3.2276712	0.37973153	0.01198531	-0.002956793	-0.00902852	0.001383877	0.924506095	Adverse	ZEB1	ENSG00000148516	0.00445024
1	3.2215002	1.2153214	0.00463686	-9.57E-05	-0.0045412	0.001413012	0.540561573	Adverse	PRKAA1	ENSG00000132356	0.004512587
1	3.22115463	3.23763921	0.00548369	0.001272177	-0.006755869	0.00141466	0.143928531	Adverse	TPM4	ENSG00000167460	0.004512587
1	3.21665212	1.23132763	0.02502766	-0.001181813	-0.023845852	0.001436299	0.534298004	Adverse	CCDC126	ENSG00000169193	0.004568353
1	3.2070455	1.95971952	0.0035301	0.000153515	-0.003683611	0.001483495	0.323086637	Adverse	ASCC3	ENSG00000112249	0.00467301
1	3.20618858	0.48507546	0.00594966	-0.004709274	-0.001240387	0.001487774	0.972264586	Adverse	NCOA1	ENSG00000084676	0.004676351

1	3.20141149	0.24995086	0.00536559	-0.003855971	-0.001509622	0.001511839	0.765780637	Adverse	MARCHF7	ENSG00000136536	0.004740743
1	3.19903979	0.00411066	0.04043634	-0.02130165	-0.019134686	0.001523919	0.102241716	Adverse	FGD4	ENSG00000139132	0.004773226
1	3.17950813	3.92807751	0.00476632	0.001575185	-0.00634151	0.001626859	0.094972402	Adverse	ATP11B	ENSG00000058063	0.005023061
1	3.16180582	1.58607473	0.00750793	0.000234081	-0.007742012	0.001725676	0.415775685	Adverse	RCL1	ENSG00000120158	0.005274062
1	3.15630396	1.2404899	0.01528887	-0.000403016	-0.014885856	0.0017575	0.530753317	Adverse	ORC4	ENSG00000115947	0.005365408
1	3.1527099	0.70375567	0.00845486	-0.001118684	-0.007336174	0.001778581	0.803049207	Adverse	ANKRD12	ENSG00000101745	0.005423791
1	3.15205805	0.76128667	0.00613571	-0.000686957	-0.005448749	0.001782429	0.765852327	Adverse	FOCAD	ENSG00000188352	0.005429554
1	3.15042932	0.19057174	0.0053248	-0.00162682	-0.003697982	0.001792078	0.675117614	Adverse	IRAK3	ENSG00000090376	0.005452955
1	3.14762955	0.07425416	0.00561557	-0.002171258	-0.003444316	0.001808778	0.429518924	Adverse	OXNAD1	ENSG00000154814	0.005485704
1	3.13995878	3.37778995	0.0066447	0.001938454	-0.008583153	0.001855271	0.132161217	Adverse	DTL	ENSG00000143476	0.005590012
1	3.1350033	0.05923819	0.00524874	-0.002106615	-0.003142122	0.00188589	0.384591826	Adverse	TMEM71	ENSG00000165071	0.005676099
1	3.13232864	3.58040374	0.00488556	0.001422307	-0.006307864	0.00190261	0.11692988	Adverse	UBA2	ENSG00000126261	0.005714013
1	3.11659639	0.25963377	0.00555098	-0.001508553	-0.004042432	0.002003754	0.779256263	Adverse	PIP5K1C	ENSG00000186111	0.00598535
1	3.11388497	0.01318063	0.00710999	-0.003913047	-0.003196943	0.002021679	0.182803711	Adverse	PATL1	ENSG00000166889	0.006019434
1	3.10382626	0.6680966	0.00709344	-0.000941935	-0.006151504	0.002089475	0.827432016	Adverse	STK39	ENSG00000198648	0.00619468
1	3.09678521	1.80350598	0.013456	0.000778908	-0.014234907	0.002138173	0.358578432	Adverse	GALK2	ENSG00000156958	0.006332282
1	3.08397421	0.04977543	0.00578948	-0.002311165	-0.003478314	0.002229469	0.353090981	Adverse	ABR	ENSG00000159842	0.006561784
1	3.08372243	0.37078904	0.00757832	-0.00170674	-0.005871583	0.002231298	0.91485102	Adverse	CNIH4	ENSG00000143771	0.006561784
1	3.0790626	2.46870112	0.00648469	0.001098359	-0.007583047	0.002265412	0.232267718	Adverse	LRCH3	ENSG00000186001	0.006631176
1	3.07063457	2.87797126	0.00392226	0.000958418	-0.004880682	0.002328335	0.179598186	Adverse	EIF5	ENSG00000100664	0.006786694
1	3.05826043	4.93617751	0.00661923	0.003189005	-0.009808237	0.002423638	0.052600276	Adverse	LMTK2	ENSG00000164715	0.007019087
1	3.05758518	4.86481319	0.01145573	0.005582136	-0.017037861	0.00242894	0.054819532	Adverse	BAZ1A	ENSG00000198604	0.007019087
1	3.05623706	0.06199754	0.00479936	-0.002886664	-0.001912697	0.002439559	0.393267683	Adverse	FAM135A	ENSG00000082269	0.007036895
1	3.05440565	0.32767574	0.00373041	-0.000932708	-0.002797699	0.002454052	0.865938172	Adverse	ANP32E	ENSG00000143401	0.007071342
1	3.0260425	0.00291182	0.00449536	-0.002353845	-0.002141518	0.002688851	0.086067922	Adverse	HDAC9	ENSG00000048052	0.007621161
1	3.02566943	0.97874392	0.00537388	-0.000302987	-0.005070894	0.002692073	0.645018217	Adverse	STAG1	ENSG00000118007	0.007622498
1	3.02434729	4.20041109	0.00425882	0.001685346	-0.00594417	0.002703518	0.080828362	Adverse	FGGY	ENSG00000172456	0.00763808
1	3.0098386	0.07771866	0.0046678	-0.001754139	-0.002913658	0.002832082	0.439173707	Adverse	SP140L	ENSG00000185404	0.007928546
1	3.00957335	1.9412273	0.00803445	0.001110775	-0.009145221	0.002834484	0.327070676	Adverse	EMSY	ENSG00000158636	0.007928546
1	3.00835325	3.0560077	0.00548357	0.001700584	-0.007184151	0.002845556	0.160878166	Adverse	AK9	ENSG00000155085	0.007951493
1	3.00418826	3.54582636	0.01305598	0.004738029	-0.01779401	0.002883651	0.11939085	Adverse	MCTP2	ENSG00000140563	0.008049829

1	2.9986195	4.22776699	0.0068302	0.002780902	-0.009611102	0.002935317	0.079535456	Adverse	NSD2	ENSG00000109685	0.008177586
1	2.99532855	3.58783928	0.00347953	0.001255828	-0.004735355	0.002966247	0.116407749	Adverse	EXOC1	ENSG00000090989	0.008252503
1	2.99020397	2.94261599	0.0054148	0.001292895	-0.006707691	0.003015007	0.17254182	Adverse	DYRK1A	ENSG00000157540	0.008362463
1	2.98956457	2.95607561	0.0225154	0.006889131	-0.029404534	0.003021142	0.171110722	Adverse	SENP6	ENSG00000112701	0.008366239
1	2.98029105	4.94997639	0.00544326	0.002707743	-0.008151004	0.003111418	0.052182049	Adverse	SEC22A	ENSG00000121542	0.008573409
1	2.9791392	0.82489779	0.00306959	-0.000352035	-0.002717553	0.003122802	0.727504098	Adverse	MRE11	ENSG00000020922	0.008579193
1	2.97632661	2.59479184	0.00488492	0.001167594	-0.006052516	0.003150759	0.21443105	Adverse	ABCC1	ENSG00000103222	0.008638878
1	2.97539656	0.23781483	0.00309028	-0.000875273	-0.002215003	0.003160055	0.748422329	Adverse	VPS54	ENSG00000143952	0.008655802
1	2.97484571	0.38364748	0.05881654	-0.012120396	-0.046696147	0.003165572	0.928684792	Adverse	IRAK3	ENSG00000090376	0.008662355
1	2.97358369	2.95162679	0.00330077	0.000972235	-0.004273004	0.003178245	0.171582318	Adverse	CEP350	ENSG00000135837	0.008679898
1	2.97130118	0.35660038	0.00508257	-0.001131289	-0.003951278	0.003201285	0.899199232	Adverse	KPNB1	ENSG00000108424	0.008734214
1	2.94870181	0.19812312	0.00708039	-0.002140694	-0.004939695	0.003437798	0.687519809	Adverse	NNT	ENSG00000112992	0.00931803
1	2.94714578	0.14343961	0.01042062	-0.007035629	-0.003384991	0.003454656	0.59022952	Adverse	RUNX1	ENSG00000159216	0.009351862
1	2.94027687	1.59755279	0.00651468	0.000637018	-0.007151702	0.003529984	0.412500723	Adverse	UBR2	ENSG00000024048	0.009500112
1	2.93285525	0.06599619	0.01183632	-0.004639275	-0.007197042	0.003613058	0.40548378	Adverse	CCDC134	ENSG00000100147	0.009695447
1	2.92920494	0.08024045	0.0033583	-0.001269719	-0.002088578	0.00365457	0.446056346	Adverse	SENP1	ENSG00000079387	0.009785731
1	2.92653594	4.53338794	0.00460725	0.002112482	-0.006719731	0.003685198	0.066479501	Adverse	XRN1	ENSG00000114127	0.009841396
1	2.92329666	1.86933037	0.01425703	0.00163736	-0.015894386	0.003722685	0.343103585	Adverse	TMEM71	ENSG00000165071	0.009931937
1	2.92219835	1.68366225	0.00467324	0.000652574	-0.00532581	0.003735474	0.38887707	Adverse	LARP4	ENSG00000161813	0.009937365
1	2.92144221	0.01573519	0.00406071	-0.002268259	-0.001792455	0.003744302	0.199649532	Adverse	PIK3CA	ENSG00000121879	0.0099513
2	5.70079554	0.56192497	-0.004914	0.01414833	-0.009234307	2.81E-08	0.906971364	Favorable	VPS13D	ENSG00000048707	6.10E-07
2	5.46235871	4.52194706	-0.0007634	0.010018654	-0.009255253	9.76E-08	0.066925457	Favorable	PHF3	ENSG00000118482	1.75E-06
2	5.16283906	0.24551765	-0.0039286	0.00979964	-0.005871014	4.40E-07	0.759501856	Favorable	HOOK3	ENSG00000168172	6.22E-06
2	5.0929678	1.00788133	-0.002255	0.007575168	-0.005320166	6.19E-07	0.630821883	Favorable	ZC3HC1	ENSG00000091732	8.36E-06
2	4.96701896	0.02502624	-0.020061	0.037709857	-0.017648843	1.13E-06	0.251396611	Favorable	OGA	ENSG00000198408	1.39E-05
2	4.92302705	0.52215962	-0.002916	0.008192573	-0.005276585	1.40E-06	0.939843801	Favorable	ITPRID2	ENSG00000138434	1.66E-05
2	4.81654462	1.27134981	-0.0073954	0.009570797	-0.002175392	2.30E-06	0.519027219	Favorable	BAZ1A	ENSG00000198604	2.47E-05
2	4.65641104	4.28846051	-0.0001507	0.009576957	-0.009426276	4.81E-06	0.076743791	Favorable	DOP1B	ENSG00000142197	4.58E-05
2	4.61864247	3.33065126	-0.000685	0.01265652	-0.011971569	5.70E-06	0.135999887	Favorable	FYB1	ENSG00000082074	5.32E-05
2	4.42261041	0.22819637	-0.0084749	0.021796541	-0.013321641	1.36E-05	0.73427114	Favorable	TM7SF3	ENSG00000064115	0.000107875
2	4.3529538	0.37185842	-0.0098739	0.028209915	-0.018335994	1.84E-05	0.916013985	Favorable	PAIP2	ENSG00000120727	0.000140153

2	4.32335453	0.05298739	-0.00425	0.00761372	-0.003363713	2.08E-05	0.364111572	Favorable	ZNF250	ENSG00000196150	0.000155249
2	4.28314074	0.17744109	-0.0036591	0.009431272	-0.005772199	2.47E-05	0.652836781	Favorable	ARFGEF1	ENSG00000066777	0.000173942
2	4.13841577	2.25156665	-0.0028491	0.020199211	-0.017350088	4.53E-05	0.266958413	Favorable	SEPTIN6	ENSG00000125354	0.000285973
2	4.10018072	2.24642128	-0.0006789	0.005705878	-0.005026998	5.30E-05	0.267847614	Favorable	MAP3K4	ENSG00000085511	0.000328647
2	4.0956746	1.46453099	-0.0136184	0.017165416	-0.003547031	5.40E-05	0.452421134	Favorable	FBXL13	ENSG00000161040	0.000334013
2	4.08616864	2.59172629	-0.0003931	0.006428364	-0.006035268	5.61E-05	0.214846393	Favorable	FAM210A	ENSG00000177150	0.000344935
2	4.07276896	4.46771335	0.00071116	0.007250992	-0.007962157	5.93E-05	0.069082377	Favorable	SYPL1	ENSG00000008282	0.000360312
2	3.99400382	0.00152263	-0.0024187	0.004742955	-0.002324227	8.15E-05	0.062252509	Favorable	EEA1	ENSG00000102189	0.000464761
2	3.99187101	2.53981187	-0.0002124	0.005888901	-0.005676454	8.22E-05	0.222016025	Favorable	ITM2B	ENSG00000136156	0.000467789
2	3.96057208	3.16926118	-4.38E-05	0.006472199	-0.006428415	9.31E-05	0.150072765	Favorable	SYNRG	ENSG00000275066	0.000516307
2	3.94170196	0.92000587	-0.0024697	0.010871778	-0.00840207	0.000100382	0.674946863	Favorable	G3BP1	ENSG00000145907	0.000544539
2	3.93853805	0.53800964	-0.0015527	0.005460799	-0.00390814	0.000101649	0.926517535	Favorable	TAF2	ENSG00000064313	0.000550337
2	3.93787331	1.2619448	-0.0009886	0.004878521	-0.003889947	0.000101918	0.522566573	Favorable	VIRMA	ENSG00000164944	0.000550696
2	3.92908841	0.48307721	-0.006385	0.00876453	-0.002379515	0.000105526	0.974063523	Favorable	UBAP2	ENSG00000137073	0.000563617
2	3.92338869	1.30151476	-0.0010014	0.005342748	-0.004341376	0.000107931	0.507873443	Favorable	HSPA9	ENSG00000113013	0.000574252
2	3.91969984	1.71961349	-0.0008073	0.005545397	-0.004738133	0.000109516	0.37948561	Favorable	WAPL	ENSG00000062650	0.000578242
2	3.9043767	0.08291934	-0.0028601	0.006790149	-0.003930079	0.000116336	0.453240973	Favorable	PARN	ENSG00000140694	0.000602045
2	3.8912312	0.6830892	-0.0017663	0.00645032	-0.004684055	0.000122506	0.81704999	Favorable	HSPA9	ENSG00000113013	0.000631202
2	3.88962675	0.67838941	-0.0051321	0.019031711	-0.013899617	0.00012328	0.820283754	Favorable	CLIP1	ENSG00000130779	0.000634012
2	3.84865707	1.33249738	-0.0018865	0.014641582	-0.012755049	0.000144686	0.496722834	Favorable	GON4L	ENSG00000116580	0.000723954
2	3.80063101	0.29771706	-0.0161636	0.04471317	-0.028549595	0.000174254	0.82936566	Favorable	FLT3	ENSG00000122025	0.000845942
2	3.75571284	1.59079031	-0.0009917	0.00564134	-0.004649614	0.000207002	0.414426512	Favorable	KDM1A	ENSG00000004487	0.000968966
2	3.69267577	2.14882285	-0.0003821	0.005932973	-0.00555092	0.000262862	0.285358491	Favorable	EPRS1	ENSG00000136628	0.00119061
2	3.66419298	0.0010631	-0.0025932	0.005089035	-0.002495794	0.000292512	0.05202113	Favorable	IBTK	ENSG00000005700	0.001305706
2	3.62167572	2.57002507	-0.0001518	0.036397233	-0.036245391	0.000342683	0.217812012	Favorable	PTPN22	ENSG00000134242	0.001477321
2	3.61763776	0.76013378	-0.0104281	0.01411093	-0.003682826	0.000347847	0.766573322	Favorable	ASPH	ENSG00000198363	0.001495563
2	3.60147091	1.22331259	-0.0036994	0.004576024	-0.00087666	0.000369264	0.537423067	Favorable	CAPN15	ENSG00000103326	0.001567533
2	3.59428342	0.07503928	-0.0036635	0.006233425	-0.002569953	0.000379178	0.43172776	Favorable	VMP1	ENSG00000062716	0.001602257
2	3.58921726	2.37224469	-0.0001659	0.00555345	-0.005387563	0.000386315	0.247018688	Favorable	RIC8B	ENSG00000111785	0.001627407
2	3.56628918	3.87386344	0.00130024	0.007935949	-0.009236184	0.000420225	0.09808701	Favorable	KNTC1	ENSG00000184445	0.0017412
2	3.5547204	0.19052301	-0.0020551	0.00572648	-0.003671409	0.000438376	0.675036641	Favorable	VPS13B	ENSG00000132549	0.001794946

2	3.55311236	0.45025729	-0.0027305	0.009575511	-0.006845041	0.000440957	0.995574405	Favorable	TM7SF3	ENSG00000064115	0.001802849
2	3.55212828	4.25065982	0.00243944	0.013740478	-0.01617992	0.000442543	0.078470168	Favorable	ATF7IP	ENSG00000171681	0.001806669
2	3.54358479	0.63072898	-0.0027194	0.01103054	-0.008311097	0.00045654	0.854176091	Favorable	USP7	ENSG00000187555	0.001847489
2	3.54215312	0.49068572	-0.0129986	0.017438768	-0.004440165	0.000458926	0.967243158	Favorable	MRPS5	ENSG00000144029	0.001851737
2	3.51358488	3.63544286	0.00104385	0.009369101	-0.01041295	0.00050905	0.113123109	Favorable	KDM1A	ENSG00000004487	0.00200154
2	3.45921833	0.81122362	-0.0012884	0.005513214	-0.004224833	0.0006189	0.735517471	Favorable	CCDC18	ENSG00000122483	0.002346909
2	3.43360754	1.66093386	-0.0005904	0.006793879	-0.006203453	0.000677997	0.394954567	Favorable	MAP2K4	ENSG00000065559	0.002515939
2	3.40610033	0.98118785	-0.0009168	0.004323354	-0.003406528	0.000747316	0.643811441	Favorable	TM7SF3	ENSG00000064115	0.002711466
2	3.3874541	0.96599556	-0.0035608	0.020256769	-0.01669597	0.000798008	0.651361817	Favorable	UBAP2	ENSG00000137073	0.002872827
2	3.37653079	0.00155908	-0.0038857	0.007563986	-0.00367825	0.000829175	0.062992908	Favorable	TRAPPC8	ENSG00000153339	0.002965772
2	3.36125715	3.45381189	-0.0094086	0.008180596	0.00122804	0.000874661	0.126212147	Favorable	USO1	ENSG00000138768	0.003104432
2	3.35312261	0.51079115	-0.0018894	0.007194886	-0.005305533	0.000899825	0.949593144	Favorable	CARF	ENSG00000138380	0.003184425
2	3.32321702	2.34649527	0.00046626	0.009878182	-0.010344446	0.000998249	0.251130051	Favorable	NCOA4	ENSG00000266412	0.003454615
2	3.31696249	0.02256125	-0.0051464	0.011429809	-0.006283359	0.001020061	0.238792694	Favorable	ZNF525	ENSG00000203326	0.003525699
2	3.31530503	4.74186394	0.00270508	0.012348818	-0.015053893	0.001025916	0.058874322	Favorable	PHC3	ENSG00000173889	0.003532062
2	3.31191434	1.33573976	-0.0011057	0.009546836	-0.008441127	0.001037989	0.495573339	Favorable	NSD1	ENSG00000165671	0.003561022
2	3.30869417	2.02789748	-0.0001129	0.007222529	-0.007109635	0.001049577	0.30886825	Favorable	CEP295	ENSG00000166004	0.00358304
2	3.29009545	4.17912067	0.0015433	0.008921634	-0.010464937	0.001118896	0.081849858	Favorable	ICE2	ENSG00000128915	0.003764054
2	3.27796803	0.30883798	-0.003737	0.012223825	-0.008486831	0.001166362	0.843211755	Favorable	KLHL24	ENSG00000114796	0.003904778
2	3.24068699	3.24282176	0.00060652	0.005506091	-0.006112611	0.001324243	0.143473974	Favorable	CDK5RAP2	ENSG00000136861	0.004310856
2	3.22970965	3.97760137	0.00202925	0.014247962	-0.016277215	0.001374376	0.092218359	Favorable	UBAP2	ENSG00000137073	0.004435123
2	3.22289928	2.68767102	0.00014772	0.00471633	-0.004864053	0.001406357	0.202255056	Favorable	ZDHHC20	ENSG00000180776	0.004507367
2	3.22252266	1.69721239	-0.0003291	0.006476876	-0.006147771	0.001408146	0.385305879	Favorable	GOLM2	ENSG00000166734	0.004507367
2	3.21755155	0.73396813	-0.0017614	0.008969609	-0.007208254	0.001431952	0.783199961	Favorable	ATP23	ENSG00000166896	0.004562494
2	3.2096059	0.54508224	-0.0012509	0.004812759	-0.00356185	0.001470778	0.920668157	Favorable	MCCC2	ENSG00000131844	0.004654105
2	3.18096499	1.61972914	-0.0002257	0.013266201	-0.013040542	0.001618965	0.406259118	Favorable	USP33	ENSG00000077254	0.005016757
2	3.18081637	0.20605653	-0.007036	0.010829131	-0.003793094	0.001619768	0.70024806	Favorable	DPY19L1P1	ENSG00000229358	0.005016757
2	3.177897	3.68450749	0.00157357	0.00883343	-0.010407001	0.001635632	0.109840379	Favorable	CLK1	ENSG00000013441	0.005043349
2	3.17517445	2.75328295	0.00062913	0.007115279	-0.007744409	0.001650555	0.194109927	Favorable	ME2	ENSG00000082212	0.005078066
2	3.17476882	3.4348939	0.0024049	0.014795996	-0.017200897	0.001652789	0.12766537	Favorable	MRPS35	ENSG00000061794	0.005079302
2	3.16508106	1.08585146	-0.0006532	0.003863897	-0.003210695	0.001706985	0.594784446	Favorable	MASTL	ENSG00000120539	0.005226808

2	3.14257471	0.83095611	-0.0007799	0.003798009	-0.003018156	0.001839293	0.723992413	Favorable	UBAP2	ENSG00000137073	0.005553944
2	3.13110631	1.59624197	-0.0002148	0.004506717	-0.004291917	0.001910296	0.412873184	Favorable	ZC3HAV1	ENSG00000105939	0.005730888
2	3.12434839	0.02156823	-0.0120516	0.021829031	-0.009777433	0.001953311	0.233516935	Favorable	ANKRD36C	ENSG00000174501	0.005847277
2	3.12078136	1.77038106	-0.0001122	0.005206595	-0.005094407	0.001976375	0.366669853	Favorable	SUSD1	ENSG00000106868	0.005909936
2	3.11022573	0.03936672	-0.0073858	0.017337112	-0.009951301	0.002046104	0.314552172	Favorable	TFDP2	ENSG00000114126	0.006085622
2	3.06451965	3.70680738	0.00179165	0.007827915	-0.00961957	0.002374991	0.108381903	Favorable	NUP98	ENSG00000110713	0.006900918
2	3.05333083	0.10978943	-0.0050597	0.008266517	-0.003206798	0.002462594	0.519232011	Favorable	ZNF737	ENSG00000237440	0.007088589
2	3.05124004	2.43942182	0.00058142	0.008498271	-0.00907969	0.00247929	0.23663966	Favorable	PARG	ENSG00000227345	0.007121856
2	3.05059345	3.24106895	0.00189997	0.012951304	-0.014851275	0.002484474	0.143627538	Favorable	SLF2	ENSG00000119906	0.00712936
2	3.04576204	4.46085951	0.0057143	0.015209655	-0.020923957	0.002523528	0.069360086	Favorable	ANKRD12	ENSG00000101745	0.007229433
2	3.03908953	0.00315321	-0.006933	0.013318814	-0.006385859	0.002578393	0.089560904	Favorable	ADGRE2	ENSG00000127507	0.007353194
2	3.03834003	1.79095423	-0.00012	0.004000402	-0.003880426	0.002584624	0.361619886	Favorable	TM7SF3	ENSG00000064115	0.007363388
2	3.01727084	4.97440852	0.0019983	0.004791334	-0.006789636	0.002765539	0.051449997	Favorable	WAPL	ENSG00000062650	0.007782816
2	3.01605324	1.22983278	-0.0037227	0.042138938	-0.038416281	0.002776341	0.534879124	Favorable	NEIL3	ENSG00000109674	0.007805292
2	2.98627816	0.04679832	-0.0076859	0.017891349	-0.010205434	0.003052856	0.342537699	Favorable	KLHL24	ENSG00000114796	0.008437205
2	2.96861235	1.23969057	-0.0006819	0.005522459	-0.004840542	0.003228622	0.531061394	Favorable	ATRX	ENSG00000085224	0.008782866
2	2.95117553	4.64238276	0.00787738	0.023874389	-0.031751764	0.003411151	0.062383332	Favorable	ANKRD17	ENSG00000132466	0.009261226
2	2.94637045	2.85109392	0.00075836	0.007059436	-0.007817792	0.003463085	0.182623573	Favorable	N4BP1	ENSG00000102921	0.009365532
2	2.92896682	3.11891532	0.00108836	0.005308366	-0.006396721	0.003657293	0.154776563	Favorable	IPO7	ENSG00000205339	0.009785731
2	2.92241773	0.89951008	-0.0021223	0.015962089	-0.013839762	0.003732916	0.685826221	Favorable	ZNF292	ENSG00000188994	0.009937365

Supplementary Table S12. Subgroup-specific analysis of splice transcripts from the host genes for the two top subgroup-specific circRNAs.

circID	GENEID	TXNAME	gene Symbol	max group	tstat	chisqstat	Adverse	Favorable	Intermediate	tpval	chpval	group
16__3850297__3851009	ENSG00000005339	ENST00000262367	CREBBP	1	0.8273	0.2377	0.151467143	0.056555427	-0.20802257	0.408732991	0.748207607	Adverse
16__3850297__3851009	ENSG00000005339	ENST00000382070	CREBBP	1	1.0492	0.0239	0.235232297	-0.065172204	-0.170060093	0.294922803	0.245550421	Adverse
16__3850297__3851009	ENSG00000005339	ENST00000571763	CREBBP	2	1.0505	1.1372	0.067748405	0.101096525	-0.16884493	0.294328262	0.572505108	Favorable
16__3850297__3851009	ENSG00000005339	ENST00000576720	CREBBP	2	1.2512	0.0516	-0.015387331	0.052962289	-0.037574958	0.211822535	0.359272805	Favorable
16__3850297__3851009	ENSG00000005339	ENST00000570939	CREBBP	3	0.7264	0.3538	-0.089931438	0.022116342	0.067815096	0.468160471	0.896112589	Intermediate
16__3850297__3851009	ENSG00000005339	ENST00000574740	CREBBP	2	1.1293	0.6337	0.032677184	0.105633161	-0.138310345	0.259656616	0.851983641	Favorable
16__3850297__3851009	ENSG00000005339	ENST00000573517	CREBBP	2	1.6096	2.7753	0.107952586	0.152450875	-0.260403462	0.108511009	0.191459848	Favorable
16__3850297__3851009	ENSG00000005339	ENST00000572569	CREBBP	2	0.7069	0.0732	-0.061134527	0.064808492	-0.003673966	0.480198975	0.426612949	Favorable
16__3850297__3851009	ENSG00000005339	ENST00000573672	CREBBP	1	0.7082	0.2183	0.035457796	-0.051698618	0.016240822	0.479374568	0.719324725	Adverse
16__3850297__3851009	ENSG00000005339	ENST00000572134	CREBBP	2	1.5515	0.7857	-0.266490236	0.221459537	0.045030699	0.121816776	0.75080373	Favorable
16__3850297__3851009	ENSG00000005339	ENST00000575237	CREBBP	1	0.6580	0.2204	0.059377321	-0.090402294	0.031024973	0.511061829	0.722524181	Adverse
16__3850297__3851009	ENSG00000005339	ENST00000634839	CREBBP	2	1.1975	0.2068	-0.171348596	0.183321299	-0.011972703	0.232054949	0.701366038	Favorable
1__12275825__12278038	ENSG00000048707	ENST00000613099	VPS13D	3	0.8252	0.9249	-0.341103969	0.14727544	0.193828528	0.409930626	0.672399892	Intermediate
1__12275825__12278038	ENSG00000048707	ENST00000476169	VPS13D	3	0.9020	1.5636	-0.089560939	0.040345828	0.04921511	0.367754857	0.422279312	Intermediate
1__12275825__12278038	ENSG00000048707	ENST00000489961	VPS13D	2	3.9556	2.1890	-0.270317525	0.290700277	-0.020382752	9.50E-05	0.277993222	Favorable
1__12275825__12278038	ENSG00000048707	ENST00000646917	VPS13D	1	1.6987	0.3025	0.034248659	-0.033377907	-0.000870752	0.090387529	0.835383422	Adverse
1__12275825__12278038	ENSG00000048707	ENST00000460333	VPS13D	3	0.7173	0.0994	-0.023115652	-0.000415038	0.02353069	0.473708813	0.494956205	Intermediate
1__12275825__12278038	ENSG00000048707	ENST00000487188	VPS13D	2	0.8011	0.8082	-0.043154014	0.024053977	0.019100037	0.423704547	0.737281602	Favorable
1__12275825__12278038	ENSG00000048707	ENST00000643711	VPS13D	1	0.1940	0.0298	0.009054355	-0.015981846	0.006927491	0.846327528	0.274147389	Adverse
1__12275825__12278038	ENSG00000048707	ENST00000469054	VPS13D	3	0.9512	0.1733	-0.045098151	-0.003113688	0.048211839	0.342254206	0.645592741	Intermediate
1__12275825__12278038	ENSG00000048707	ENST00000466732	VPS13D	3	2.0091	1.9124	0.0025391	-0.047327593	0.044788493	0.045412487	0.333383819	Intermediate
1__12275825__12278038	ENSG00000048707	ENST00000543766	VPS13D	1	1.8303	0.0241	0.004434643	-0.001699003	-0.00273564	0.068178872	0.24665214	Adverse
1__12275825__12278038	ENSG00000048707	ENST00000645148	VPS13D	1	1.1660	0.4549	0.025068777	0.007642678	-0.032711455	0.244539496	0.999980513	Adverse
1__12275825__12278038	ENSG00000048707	ENST00000473099	VPS13D	2	0.3401	0.0368	0.001086024	0.00798698	-0.009073004	0.73399629	0.304388321	Favorable

Supplementary Table S13. Multivariable Cox regression analysis for circRNA 7__77607235__77632425.

	**coef**	**exp(coef)**	**se(coef)**	**z**	**Pr(>|z|)**
7__77607235__77632425	0.868715284	2.38384632	0.352797819	2.462360132	0.0138026
age	0.054593539	1.056111259	0.013423265	4.0670835	4.76E-05
sex	-0.014791724	0.985317136	0.291217608	-0.050792685	0.95949072
FLT3	0.297554597	1.346561891	0.349060988	0.852443001	0.393968262
NPM1	-0.376055245	0.686564403	0.345945844	-1.087035014	0.277021336
TP53	NA	NA	0	NA	NA
CBFB.MYH11	0.947613146	2.579545307	0.611296826	1.550168602	0.121101054
PML.RARA	-18.37041642	1.05E-08	4225.755219	-0.00434725	0.996531407
RUNX1.RUNX1T1	0.822811298	2.27689187	0.671229044	1.225827913	0.220263441
MLL	NA	NA	0	NA	NA
inv.3	NA	NA	0	NA	NA

Supplementary Table S14. Survival analysis of splice transcripts of PTPN12, the host gene of 7__77607235__77632425.

CircRNA	Gene	Transcript	coef	exp(coef)	se(coef)	z	Pvalue	FDR
7__77607235__77632425	PTPN12	ENST00000447995	0.505231467	1.657369103	0.276462967	1.827483345	0.067627128	0.673999815
7__77607235__77632425	PTPN12	ENST00000433369	-1.00259741	0.366925147	0.720946536	-1.390668185	0.16432607	0.673999815
7__77607235__77632425	PTPN12	ENST00000523952	0.3809486	1.463672371	0.274159466	1.389514672	0.164676302	0.673999815
7__77607235__77632425	PTPN12	ENST00000440186	-0.994109163	0.370052954	0.721917542	-1.377039766	0.168499954	0.673999815
7__77607235__77632425	PTPN12	ENST00000418110	-0.34296446	0.709663433	0.293176731	-1.169821555	0.242072787	0.770421197
7__77607235__77632425	PTPN12	ENST00000481154	0.250479444	1.284641183	0.27372645	0.915072123	0.360153768	0.770421197
7__77607235__77632425	PTPN12	ENST00000407343	-0.246060064	0.781875261	0.274041258	-0.89789423	0.369241941	0.770421197
7__77607235__77632425	PTPN12	ENST00000460731	0.196979946	1.21771962	0.273162696	0.721108513	0.470842753	0.770421197
7__77607235__77632425	PTPN12	ENST00000522115	-0.188753574	0.82799052	0.3039649	-0.620971612	0.534618302	0.770421197
7__77607235__77632425	PTPN12	ENST00000519553	0.16768747	1.182566965	0.272597567	0.615146613	0.538457901	0.770421197
7__77607235__77632425	PTPN12	ENST00000494248	-0.158164889	0.853709003	0.273401054	-0.578508701	0.562920724	0.770421197
7__77607235__77632425	PTPN12	ENST00000415482	-0.148392505	0.862092673	0.277321644	-0.535091683	0.592586461	0.770421197
7__77607235__77632425	PTPN12	ENST00000435495	-0.13325117	0.875245229	0.273385742	-0.487410826	0.625967223	0.770421197
7__77607235__77632425	PTPN12	ENST00000520947	0.060198418	1.062047255	0.272547129	0.220873426	0.82519099	0.943075417
7__77607235__77632425	PTPN12	ENST00000464313	0.034037305	1.034623203	0.272727189	0.124803492	0.900679123	0.960724398
7__77607235__77632425	PTPN12	ENST00000248594	0.003973148	1.003981052	0.273041394	0.014551451	0.988390032	0.988390032
